# Agronomic efficiency and genome mining analysis of the wheat-biostimulant rhizospheric bacterium *Pseudomonas pergaminensis* sp. nov. strain 1008^T^

**DOI:** 10.3389/fpls.2022.894985

**Published:** 2022-07-28

**Authors:** Marisa Díaz, Teresa Bach, Gustavo González Anta, Betina Agaras, Daniel Wibberg, Fabián Noguera, Wilter Canciani, Claudio Valverde

**Affiliations:** ^1^Rizobacter Argentina S.A., Buenos Aires, Argentina; ^2^Escuela de Ciencias Agrarias, Exactas y Naturales, Universidad Nacional del Noroeste de la Provincia de Buenos Aires (UNNOBA), Buenos Aires, Argentina; ^3^Departamento de Ciencias Naturales y Exactas, Universidad Nacional de San Antonio de Areco (UNSAdA), Buenos Aires, Argentina; ^4^Indrasa Biotecnología S.A., Córdoba, Argentina; ^5^Laboratorio de Fisiología y Genética de Bacterias Beneficiosas para Plantas, Centro de Bioquímica y Microbiología del Suelo, Universidad Nacional de Quilmes-CONICET, Buenos Aires, Argentina; ^6^Center for Biotechnology (CeBiTec), Bielefeld University, Bielefeld, Germany

**Keywords:** PGPR, *Pseudomonas pergaminensis* sp. nov., wheat, biostimulant, agronomic efficiency, genome mining, phylogenomics

## Abstract

*Pseudomonas* sp. strain 1008 was isolated from the rhizosphere of field grown wheat plants at the tillering stage in an agricultural plot near Pergamino city, Argentina. Based on its *in vitro* phosphate solubilizing capacity and the production of IAA, strain 1008 was formulated as an inoculant for bacterization of wheat seeds and subjected to multiple field assays within the period 2010–2017. *Pseudomonas* sp. strain 1008 showed a robust positive impact on the grain yield (+8% on average) across a number of campaigns, soil properties, seed genotypes, and with no significant influence of the simultaneous seed treatment with a fungicide, strongly supporting the use of this biostimulant bacterium as an agricultural input for promoting the yield of wheat. Full genome sequencing revealed that strain 1008 has the capacity to access a number of sources of inorganic and organic phosphorus, to compete for iron scavenging, to produce auxin, 2,3-butanediol and acetoin, and to metabolize GABA. Additionally, the genome of strain 1008 harbors several loci related to rhizosphere competitiveness, but it is devoid of biosynthetic gene clusters for production of typical secondary metabolites of biocontrol representatives of the *Pseudomonas* genus. Finally, the phylogenomic, phenotypic, and chemotaxonomic comparative analysis of strain 1008 with related taxa strongly suggests that this wheat rhizospheric biostimulant isolate is a representative of a novel species within the genus *Pseudomonas*, for which the name *Pseudomonas pergaminensis* sp. nov. (type strain 1008^T^ = DSM 113453^T^ = ATCC TSD-287^T^) is proposed.

## Introduction

The rhizosphere of terrestrial plants harbors complex assemblages of bacteria that are fundamental for the nutrition, health, and tolerance to biotic and abiotic stresses of the plant host (Mendes et al., 2013), being this interkingdom association a consequence of a long co-evolutionary history that shaped the current plant holobionts (Rosenberg and Zilber-Rosenberg, 2016; Lyu et al., 2021). Those plant-beneficial bacteria are collectively referred to as plant growth-promoting rhizobacteria (PGPR), a concept introduced in the late 70’s (Kloepper et al., 1980). Although the taxonomic composition of the rhizosphere microbiome is certainly influenced by the plant genotype, soil features, agronomical practices, and environmental factors (Philippot et al., 2013), there are ubiquitous bacterial groups that are hallmark components of the rhizosphere niche, such as members of the *Streptomyces, Flavobacterium, Bacillus, Burkholderia*, and *Pseudomonas* genera (Mendes et al., 2011; Rey and Dumas, 2017; Imade and Babalola, 2021; Kavamura et al., 2021).

In particular, *Pseudomonas* spp. strains display a strong preference for the rhizosphere habitat in comparison to bulk soil, a feature that has been revealed by both culture-dependent and culture-independent approaches (Molina et al., 2000; Agaras et al., 2012; Tao et al., 2020; Chiniquy et al., 2021). The relative ease to isolate and culture *Pseudomonas* strains in the lab, together with the identification and characterization of a number of genetic and biochemical traits that may significantly contribute to the growth and health of the host plant, has fostered the exploitation of a number of *Pseudomonas* isolates for the development of agricultural inputs to increase the yield of different crops. The latter represents a promising conceptual solution to the worldwide demand of eco-friendly alternatives to the questioned utilization of chemical inputs in agriculture (Backer et al., 2018; Höfte, 2021; Müller and Behrendt, 2021).

A group of isolates of the genus *Pseudomonas* that have become plant biocontrol models, like *Pseudomonas protegens* CHA0, *Pseudomonas synxantha* 2–79. *Pseudomonas chlororaphis* PA23, or *Pseudomonas simiae* WCS417, have the ability to protect the roots of colonized plants from diverse phytopathogens, either through the production of antimicrobial compounds and/or by inducing systemic resistance in the plant (Ramette et al., 2011; Duke et al., 2017; Zhang et al., 2020; Höfte, 2021; Pieterse et al., 2021). On the other hand, rhizospheric isolates like *P. fluorescens* SS101 or *P. fluorescens* SBW25, do not show biocontrol capacity at all, but display plant growth-promoting traits like strong root competitiveness, stimulation of root growth and/or modulation of hormone homeostasis (Park et al., 2015; Shinde et al., 2019), and fall within the definition of plant biostimulants (Du Jardin, 2015). Usually, the *in vitro* characterization of such indirect (biocontrol) or direct (biostimulant) traits has been further mined at the genetic and/or genomic level, to unravel the underlying molecular determinants (Paterson et al., 2017; Agaras et al., 2018; Biessy et al., 2019; Pieterse et al., 2021), which may ultimately lead to biotechnological improvements of the identified plant-beneficial traits (Haskett et al., 2021).

Nevertheless, only a few thoroughly characterized *Pseudomonas* isolates with plant-beneficial traits have been registered worldwide as active ingredients for the production of commercial agricultural inputs (Basu et al., 2021; Höfte, 2021), a fact that most likely reflects the hurdles existing in the transition from the lab to the field (Backer et al., 2018). Here, we report the isolation of the *Pseudomonas* sp. isolate 1008 from the rhizosphere of field-grown wheat, which proved to be a highly efficient biostimulant of wheat crops in field assays under agronomical conditions. Further, we have determined its full genome sequence, explored its plant-beneficial genetic traits, and carried out phylogenomic, phenotypic and chemotaxonomic comparative analyses with related taxa, which strongly support that strain 1008 is a plant-biostimulant representative of a novel *Pseudomonas* species.

## Materials and methods

### Strain isolation and characterization

**FIGURE 1:**
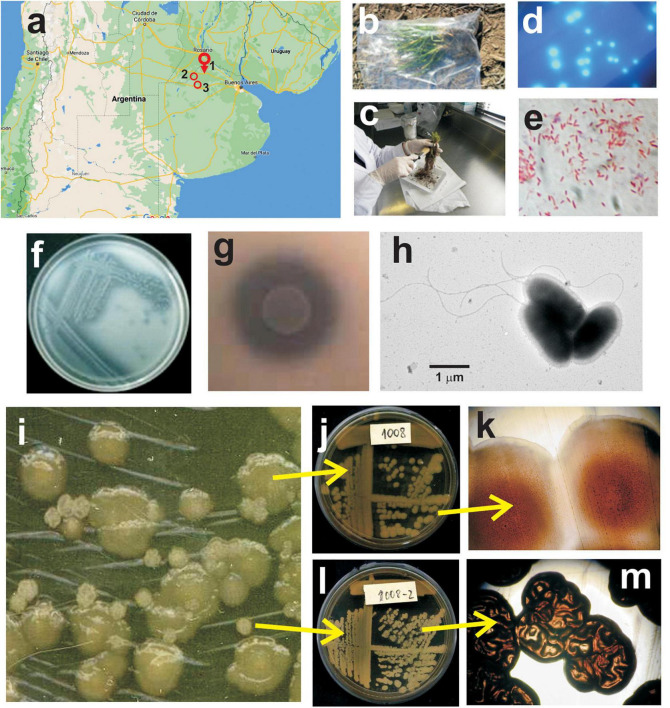
Isolation and microbiological characterization of *Pseudomonas* sp. strain 1008. **(a)** Geographic location of the wheat field plot sampled for isolation of *Pseudomonas* strain 1008 (1, Pergamino), and of the experimental field assays for evaluation of the performance of the inoculant based on strain 1008 (1, Pergamino; 2, Ferré; 3, Junín). **(b)** Wheat plants sampled at tillering stage. **(c)** Loosely adhered soil was gently brushed away from the root system. **(d)** Fluorescent colonies developed on *Pseudomonas* agar F upon plating of dilutions of rhizospheric suspensions. **(e)** Gram staining of cells from a pure culture of strain 1008. **(f)** Streaks of strain 1008 on Pikovskaya agar with calcium triphosphate as P source. **(g)** Close up of a macrocolony of strain 1008 and the surrounding calcium triphosphate solubilization halo on Pikovskaya agar. **(h)** Transmission electron micrograph showing cells of strain 1008 grown for 24 h in nutrient yeast broth at 28°C and 200 rpm. Note the flagella with a typical polar location. **(i)** Colony phase variants of strain 1008 on nutrient agar plates. The larger colonies correspond to phase variant 1 **(j,k)** whereas the smaller colonies correspond to phase variant 2 **(l,m)**. The colony morphotype of each variant is clearly distinct under magnifying lens **(k,m)**.

Root systems of wheat plants (stage Z.20; tillering) with their surrounding soil were sampled in a productive plot in the vicinity of Pergamino city, Province of Buenos Aires, Argentina (33° 56′ 41′′ S, 60° 34′ 04′′ W) (Figure 1a). Care was taken to avoid damaging the root systems. The plants were placed in clean plastic bags, leaving them open to avoid anoxic conditions, and were transported to the laboratory for processing without delay (Figure 1b). The aerial parts were cut, and the root systems were gently brushed to remove loosely adhered soil (Figure 1c). Each root system with its tightly associated rhizospheric soil was placed in an Erlenmeyer flask containing sterile demineralized water (pH 6.8–7) and agitated for 30 min at 200 rpm. Thereafter, aliquots of the suspensions were serially diluted with sterilized water, plated on *Pseudomonas* agar F (Merck Millipore) and incubated at 28°C for 48 h. The colonies that showed fluorescence upon exposure of plates to UV light (Figure 1d) were re-streaked for ensuring purity and preserved for subsequent characterization and preliminary identification. Colonies of interest were first screened on the basis of the conventional physiological tests according to Bergey’s Manual of Determinative Bacteriology (Palleroni, 2015) to select candidate isolates of the species *Pseudomonas fluorescens* or closely related species (i.e., Gram-negative rods that grow in cetrimide agar: positive; oxidase test: positive; glucose fermentation: negative; motility: positive; fluorescent pigments in *Pseudomonas* F medium: positive; fluorescent pigments in *Pseudomonas* P medium: negative; growth at 41°C: negative; gelatin hydrolysis: positive). Phosphate solubilization, a feature sought for the selection of candidates for the formulation of biofertilizer inoculants, was applied as the second screening criterion in agarized Pikovskaya’s medium (Pikovskaya, 1948). The phosphate solubilizing isolates preliminary identified as *P. fluorescens* were further tested with the bioMérieux API 20 NE Gallery System. The isolate 1008, which is the subject of this study and that was originally designated as a *P. fluorescens* representative on the basis of the aforementioned biochemical and physiological tests, was selected for further characterization and development of a commercial formulation for agronomical purposes.

### Phenotypic assays

Strain 1008 was routinely grown on *Pseudomonas* agar F (Merck Millipore) or nutrient agar (NA; 4% w/v blood agar base, 0.5% w/v yeast extract; Biokar). Liquid cultures were grown in tryptone soybean broth (TSB; Laboratorios Britania) or nutrient yeast broth (NYB; 2.5% w/v nutrient broth, 0.5% w/v yeast extract; Biokar), with shaking (200 rpm). In all cases, incubation temperature was 28°C. The strain was stored at −80°C as a suspension of an overnight NYB culture containing 20% w/v of glycerol. Solubilization of mineral phosphate was qualitatively assessed in agarized medium containing inorganic phosphate (Pikovskaya, 1948). The presence of a clear halo around the bacterial colonies was determined after 5 days of growth. Phosphate solubilization was quantified in liquid Pikovskaya medium with 5 g/L of Ca_3_(PO_4_)_2_ (Pikovskaya, 1948) modified by buffering with 100 mM Tris and adjusting the pH to 8.0 with HCl. Samples from triplicate cultures were withdrawn to assess soluble phosphate concentration. In total, 1 mL of the cell suspension was centrifuged at 12,000 × *g* for 20 min at 4°C, and the P content in the supernatant was determined by the molybdenum blue method (Murphy and Riley, 1962). Zinc solubilization was qualitatively assessed in agarized Pikovskaya’s base medium containing 1% w/v ZnO (Tagele et al., 2019). The presence of a clear halo around the bacterial colonies was determined after 48 h of growth. Extracellular protease and phospholipase C (lecithinase) activity were qualitatively assessed in skimmed milk agar or in egg yolk agar, respectively, as reported previously (Sacherer et al., 1994). Intracellular and extracellular phosphatase activities were determined enzymatically by following the hydrolysis of *p*-nitrophenyl phosphate in reactions buffered at pH 5.5 or pH 9.0 (for acid and alkaline phosphatase, respectively), as reported (Tabatabai and Bremner, 1969). Cellular siderophore production was qualitatively studied in CAS agar plates (Schwyn and Neilands, 1987). Hydrogen cyanide production was detected using the picrate filter paper method (Egan et al., 1998). Indoleacetic acid (IAA) production was analyzed in the supernatant of cultures grown for 24 h in tryptophan-amended medium (20 mg/100 ml) by colorimetry with the Salkowski reagent, as described elsewhere (Bric et al., 1991). The capacity of strain 1008 to inhibit the growth of phytopathogenic fungi was assessed with the dual culture assay on PDA plates (Muller et al., 2018). Analysis of cellular fatty acids and metabolic profiling in Biolog GenIII plates were carried out by DSMZ Services, Leibniz-Institut DSMZ - Deustsche Sammlung von Mikroorganismen und Zellkulturen GmbH, Brunswick, Germany.

### Transmission electron microscopy

Cells of strain 1008 from early stationary-phase cultures in NYB were negatively stained with a solution of 2% w/v uranyl acetate for 5 min and directly observed at a magnification of 15,000–40,000× with a JEOL/JEM 1200 EX II transmission electron microscope at the Microscopy Service of the School of Veterinary Sciences (National University of La Plata, La Plata, Argentina). The dimensions of cells were analyzed in digitalized images with ImageJ^1^.

### Field assays

Field assays were carried out in three different locations in the Argentine Pampas, close to the cities of Pergamino, Junín, and Ferré (Figure 1a). The properties of the soil in each location are: typic Argiudoll – silt loam, for Pergamino; typic Hapludoll – loam/silt loam, for Junín and Ferré (Agaras et al., 2020). In the period 2010–2016, the annual average temperature ranged between 15.7 and 19°C, with an average monthly temperature range of 10 to 23 °C; the annual average rainfall ranged between 656 and 1,681 mm, with an average monthly rainfall range of 26 to 182 mm (Supplementary Table 1). The chemical analyses of soil samples (contents of organic carbon, nitrate, organic nitrogen, total nitrogen, and extractable phosphorus, and pH) were determined by Laboratorio SueloFértil (Pergamino, Buenos Aires^2^) using standardized agronomical protocols.

For evaluating the impact of seed inoculation with a commercial formulation based on strain 1008, seeds of different cultivars of wheat (*Triticum aestivum*) (Supplementary Table 2) were treated with the formulated inoculant Rizofos^®^ following the instructions of the manufacturer (Rizobacter Argentina S.A.). Briefly, seeds were treated at a ratio of 0.8 ml of inoculant plus 0.2 ml of Premax R^®^ bacterial protectant per 100 g of seeds in a rotary mixer at room temperature during 2 min. Seeds were immediately sown or stored at temperatures below 25°C in the dark for a period of up to 15 days before sowing. Non-treated seeds served as control. When indicated (Supplementary Table 2), seeds had been previously treated with the commercial fungicide Compinche^®^ (Rizobacter Argentina S.A.), containing difenoconazole and metalaxyl-M as active principles. Genotypes, seed treatments and fertilizer applications were those recommended by regional agronomical advisers (Supplementary Table 2).

Each treatment had three replicates per location in a completely randomized block design, with a plot size of 9 m^2^ (6 m × 1.5 m, with 7 furrows). Sowing dates, plant densities and fungicide seed treatments were adjusted to the selected genotypes and the regional recommendations [Supplementary Table 2; (Agaras et al., 2020)]. At harvest, the number of plants and of spikes per m^2^, and grain yield (kg/ha) were recorded. When indicated (Supplementary Table 2), the number of tillers per m^2^, the tiller and spike dry weight, and the NDVI (Normalized Difference Vegetation Index, shown as the average value per m^2^, during tillering), were determined during early crop stages (Benedetti and Rossini, 1993).

### Statistical analysis of field assays

The data set comprises twenty-five field assays under no-till management across seven campaigns, heterogeneously distributed in three geographical locations. In addition to the main effect of seed bacterization with the Rizofos^®^ formulation containing live cells of strain 1008, the experimental plots included: the effect of seed pre-treatment with (or without) the fungicide Compinche^®^; five wheat genotypes (Baguette 601, Klein Tigre, Klein Rayo, Klein Yarará, and Buck SY 300); and two different forecrops (soybean or cereal/soybean). Thus, we considered that there were five additional effects that could be influencing the impact of wheat seed bacterization. First, we applied ANOVA to evaluate if those effects interacted with seed bacterization. Upon confirming that the interactions between bacterization and every additional effect were not significant, we applied Generalized Linear Mixed Models (GLMM; Di Rienzo et al., 2011) to evaluate the global effect of seed bacterization with strain 1008 on crop yield using Infostat v. 2020 software (Di Rienzo et al., 2020). GLMM were fitted to analyze the data (with *n* = 150 for grain yield, plant number and spike number per m^2^; *n* = 114 for tiller number; *n* = 34 for tiller dry weight; *n* = 102 for tiller fresh weight; *n* = 48 for spike fresh weight), considering locations, seasons, crop genotypes, forecrops and seed pre-treatment with fungicide as random effects, and amending the variance structure to achieve homoscedasticity when corresponded (Casanoves et al., 2007; Harrison et al., 2018). The fitted models were evaluated with Akaike’s (AIC) and Schwarz’s (BIC) Information Criteria, looking for the lowest values for selecting the best model. Likelihood ratio between models was applied when AIC ad BIC criteria were not sufficient to select the best model (Di Rienzo et al., 2011). Finally, ANOVA tests were performed on each of the 25 field trials to specifically evaluate the impact of seed bacterization (Di Rienzo et al., 2020). If appropriate, Fisher’s LSD multiple comparison method was applied to evaluate significant differences among average values. In all cases, statistics were done at *p* < 0.05. Multivariate analyses were conducted using Principal Component Analysis (PCA) of grain yield, plant number and tiller number per m^2^ (*n* = 150, Infostat v. 2020).

### DNA isolation and genome sequencing

Genomic DNA of strain 1008 was extracted with the Zymo ZR Soil Microbe DNA MiniPrep kit (Zymo Research), according to the manufacturer’s guidelines. gDNA concentration and quality were assessed by UV spectrophotometry with Nanodrop ND-1000. A sample of 15 μg of gDNA in 10 mM Tris.HCl (pH 8.0) was submitted to Macrogen Inc. (Korea) for whole genome *de novo* sequencing with the PacBio RS System. Upon quality control analysis (fluorescence-based quantification, agarose gel and microfluidic electrophoresis), the gDNA was processed according to a guide for preparing SMRTbell template for sequencing on the PacBio RS System. The templates were sequenced using SMRT^®^ sequencing. Sequencing resulted in a total of 134.530 subreads, with an average length of 9,422 bp, totalizing around 1,27 Gb, which represents 192× coverage of the genome.

### Genome assembly and annotation

The raw data generated from PacBio RS II sequencing was utilized for whole-genome sequence assembly. The assembly and annotation approach was performed as described recently with smaller modifications (Wibberg et al., 2020, 2021). In brief, the assembly was performed using canu v1.6 (Koren et al., 2017) resulting in a single, circular contig. This contig was then polished based on PacBio reads using quiver 2.1 (Chin et al., 2013) and adjusted to *dnaA* as the first gene. The finished genome sequence was annotated with Prokka (Seemann, 2014) and imported into the annotation platform GenDB (Meyer et al., 2003). The annotated circular and gapless chromosome of 6,609,162 bp and a GC content of 60.7% was deposited into the NCBI database under the accession number CP078013.

### Phylogenomic analysis

The phylogenetic position of strain 1008 within the genus *Pseudomonas* was inferred by a set of complementary studies based on comparative sequence analysis of single or multiple genes, and of genome properties: comparative analysis of whole genome average nucleotide identity (ANI) and tetranucleotide usage pattern (Tetra) in the JSpeciesWS server (Richter et al., 2015); average amino acid identity (AAI) at the EDGAR server (Dieckmann et al., 2021); 16S rRNA based gene tree, genome-based phylogenetic tree, and digital DDH at the Type (Strain) Genome Server (TYGS) (Meier-Kolthoff and Göker, 2019).

### Genome mining

The presence of sequences related to integrated plasmids and prophages was studied with NCBI VecScreen^3^, PlasmidFinder 2.1 and PHAST tools (Zhou et al., 2011; Carattoli et al., 2014). MGEfinder and ICEfinder were used to identify mobile genetic elements (Liu et al., 2018; Durrant et al., 2020). oriTfinder was used to identify DNA transfer-related modules (Li et al., 2018). Genomic islands were searched with IslandViewer 4 (Bertelli et al., 2017). Acquired genes and/or chromosomal mutations mediating antimicrobial resistance were inspected using the Resistance Gene Identifier tool^4^ at the Comprehensive Antibiotic Resistance Database (Alcock et al., 2020). The presence of CRISPR/Cas elements was studied with CRISPRCasFinder and CRISPRMiner2 (Couvin et al., 2018; Zhang et al., 2018). antiSMASH v6.0 was used for predicting secondary metabolite biosynthetic gene clusters (Blin et al., 2021). Bacteriocin gene clusters were mined with BaGel4 (Van Heel et al., 2018). A list of genes related to direct and indirect plant growth promotion mechanisms, as well as for rhizosphere competence and interactions with plant cells and other microbial species, was compiled from recent works (Berendsen et al., 2015; Biessy et al., 2019; Anderson and Kim, 2020; De Vrieze et al., 2020; Keswani et al., 2020; Singh et al., 2021) and surveyed in the genome of strain 1008 by using BlastN and BlastP webservices at the NCBI webpage^5^. Type IV secretion system components were identified with OriTfinder (Li et al., 2018). Type VI secretion system components were identified with SecRet6 (Li et al., 2015).

### Availability of biological material

The type strain, originally designated as 1008, was deposited in the Leibniz Institute DSMZ - German Collection of Microorganisms and Cell Cultures GmbH (DSMZ, Braunschweig, Germany) and in the American Type Culture Collection (ATCC, Manassas, VA, United States), under the accession numbers DSM 113453 and ATCC TSD-287, respectively.

## Results

### Isolation and characterization of a phosphate-solubilizing *Pseudomonas* isolate with plant growth-promoting features from field-grown wheat roots

Field-grown wheat plants at the tillering stage were sampled in an agricultural plot nearby the city of Pergamino (Buenos Aires province, Argentina; Figure 1a) to generate a collection of rhizobacterial isolates with potential to be formulated as an agricultural input. Rhizospheric suspensions were plated on *Pseudomonas* agar F, and fluorescent colonies with different morphological features were subjected to an initial set of phenotypical tests to retain those *P. fluorescens* candidates on the basis of Bergey’s determinative manual. Upon further screening for phosphate-solubilizing potential, one isolate designated as 1008 was retained for further characterization of plant-growth promoting features (Figure 1 and Table 1). The results of the bioMérieux API 20 NE Gallery System for strain 1008 were consistent with an isolate representative of the *P. fluorescens* lineage (Supplementary Table 3), which was also supported by the sequence of its 16S rDNA gene (Supplementary Figure 1).

**TABLE 1:** *In vitro* plant growth-promoting traits of *Pseudomonas* sp. strain 1008.

PGP trait	Detected (+)/Not detected (−)
Phosphate solubilization	+
Zinc solubilization	+
Alkaline phosphatase activity	+
Acid phosphatase activity	+
Tryptophan-induced IAA production	+
Lecithinase activity	+
Exoprotease activity	+
Iron sequestration in the presence of CAS	+
ACC deaminase activity	–
Hydrogen cyanide production	–
Antagonism of phytopathogenic fungi	–

Strain 1008 was selected by its phosphate-solubilizing activity in a first screening on agar plates (Figures 1f,g and Table 1). In liquid Pikovskaya medium after 24 h of growth at 28°C and 200 rpm, strain 1008 was able to solubilize an average of 22% of the insoluble calcium triphosphate (final soluble P content of 45 mg per 100 ml). It was also able to mineralize P from two types of phosphate rocks (of Chinese and Chilean origin) achieving a final soluble content of 46 mg P per 100 ml. In addition to insoluble sources of P, strain 1008 solubilized zinc in a modified Pikovskaya medium containing ZnO (Table 1 and Supplementary Figure 2A).

The production of indole acetic acid (IAA)-like compounds was first detected qualitatively as a positive reaction to the Salkowski reagent in cells grown in agarized medium supplemented with 5 mM tryptophan (Table 1). The production of IAA was confirmed by HPLC-MS/MS analysis of liquid cultures. Strain 1008 produced IAA in a tryptophan-dependent manner reaching up to 46,7 mg/100 ml after 48 h of growth (Supplementary Figure 2E). The production of extracellular proteolytic and lecithinase activities were revealed in qualitative plate assays (Table 1 and Supplementary Figures 2B,C). Spectrophotometric enzymatic assays revealed the production of both acid and alkaline phosphatases, with a major fraction of the activity detected in acid conditions (586 μg *p*-nitrophenol/mg bacteria.h) of which 83% was located intracellularly. By contrast, 82% of the total alkaline phosphatase activity (10 μg *p*-nitrophenol/mg bacteria.h) was detected in the culture supernatant.

Strain 1008 produced soluble and diffusible iron-chelating compounds detected in CAS plates (Table 1 and Supplementary Figure 2D) and it was not able to grow on ACC as the sole C source in defined medium, thus disclosing the lack of ACC deaminase activity under the tested conditions (Table 1). Finally, strain 1008 did not produce hydrogen cyanide and did not inhibit the growth of a variety of phytopathogenic fungi or an oomycete in dual culture plates (Table 1 and Supplementary Figure 2F).

Pure cultures of strain 1008 in NYB medium revealed polarly flagellated cells typical for the *Pseudomonas* genus, with average dimensions of 1.9 ± 0.4 μm long and 0.9 ± 0.1 μm wide (Figure 1h). Cells showed on average 1 or 2 polar flagella of up to 10 μm long (Figure 1h). Interestingly, strain 1008 showed evidence of colony phase variation in solid medium (Figures 1i–m). The identity of both phase variants was confirmed by 16S rDNA sequencing (Supplementary Figure 3). Whereas the colony morphotypes were clearly distinct (Figures 1i–m), both colony phase variants were undistinguishable at the cellular level (Supplementary Figure 3).

To summarize, strain 1008 was isolated from the rhizosphere of field grown wheat plants at the tillering stage and preliminary typed as a member of the *P. fluorescens* subgroup on the basis of phenotypical tests and its 16S rDNA sequence (Supplementary Figure 1; see below). *In vitro* assessment of plant growth-promoting traits revealed that strain 1008 had features that may directly impact on plant growth (such as an improvement of P availability to plant roots and production of IAA). For this reason, strain 1008 was formulated as an inoculant for wheat seed treatment and subjected to multiple field assays to evaluate its performance as a plant biostimulant.

### Agronomic efficiency of *Pseudomonas* sp. strain 1008

The impact of treating wheat seeds with Rizofos^®^, a commercial formulation based on *Pseudomonas* strain 1008, was evaluated in a series of 25 field assays carried out in the period 2010–2017 (Supplementary Table 2). This dataset involved seven campaigns in two or three locations (Figure 1a), a total of five wheat genotypes, two different forecrops, two treatments with antifungals (Compinche^®^, or none), and two treatments with strain 1008 (Rizofos^®^, or none), under no-till management. The variables that were measured in all 25 assays were grain yield, number of plants and number of spikes per m^2^ (Supplementary Table 2). These were used to carry out multivariate analyses.

**FIGURE 2:**
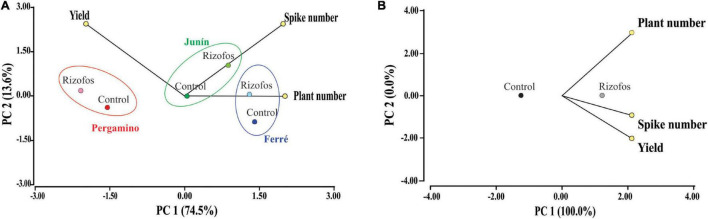
Agronomic efficiency of *Pseudomonas* sp. strain 1008. Principal component analyses of wheat parameters measured for 25 field assays (for details, see Supplementary Table 2). Data were partitioned by seed bacterization treatment with *Pseudomonas* sp. strain 1008, and by location **(A)**; or, only by seed bacterization treatment **(B)**. **(A)** In each location, wheat grain yield and the number of spikes per m^2^ mainly explain the seed bacterization effect. Together, PC1 and PC2 explain >85% of the total variance. PC1 explains the geographical effect, whereas PC2 explains the bacterization effect for each geographical site. **(B)** Seed bacterization clearly influenced the three measured variables, explaining the whole variance. In both plots, “Rizofos” refers to the seed bacterization treatment.

By considering all classifiers (location, campaign, genotype, seed treatment with antifungals, forecrop, and treatment with Rizofos^®^), we did not find a general impact of any of the factors (Supplementary Data 1). If location and treatment with Rizofos^®^ were only considered for data classification, the effect of inoculation was clear-cut, specifically based on the contribution of grain yield and spike number (Figure 2A). The impact of location was more evident in the plant number per m^2^ (Figure 2A). If we only consider the impact of seed inoculation with Rizofos^®^ in a principal component analysis, the PC1 explained 100% of the variability, with those plots in which seeds were treated with Rizofos^®^ showing the highest values for the three variables (number of plants and of spikes per m^2^, and grain yield) (Figure 2B). This global analysis of the field assay dataset thus indicated that all factors other than seed treatment with Rizofos^®^ did not strongly influence the data behavior. Thus, we next proceeded to apply a generalized linear mixed model, in which all factors (except for seed treatment with Rizofos^®^) were considered as random effects. This allowed us to evaluate the impact that treatment of seeds with the inoculant based on strain 1008 had on the different variables recorded in the field trials (Table 2 and Supplementary Data 1).

**TABLE 2:** Agronomic efficiency of *Pseudomonas* sp. strain 1008.

Variable	*n*	Control	+Rizofos^®^	SE	*P*-value
Grain yield (kg/ha)	150	4,084.4^a^	4,423.8^b^	289.0	<0.0001
Number of plants per m^2^	150	n.s.	n.s.	n.s.	0.1874
Number of spikes per m^2^	150	371.4^a^	388.3^b^	37.3	0.0274
Tiller number per m^2^	114	n.s.	n.s.	n.s.	0.6314
NDVI at tillering	*	*	*	*	*
Tiller dry weight (g)	34	100.3^a^	101.9^b^	10.4	<0.0001
Tiller fresh weight (g)	102	452.6^a^	489.9^b^	43.9	0.0007
Spike dry weight (g)	*	*	*	*	*
Spike fresh weight (g)	48	1,815.0^a^	1,719.3^b^	86.0	0.0019

Output of GLMM applied to analyze the impact of wheat seed bacterization with Pseudomonas sp. strain 1008 (Rizofos^®^) in field assays. Adjusted average values for non-bacterized (Control) and bacterized (+Rizofos^®^) treatments and associated standard error (SE) are shown. Fisher’s LSD test (α = 0.05) was applied for multiple comparisons. p-value correction was not applied. See Supplementary Data 1 for more details of the GLMM analysis. *The model could not be built. Different superscript lowercase letters (a, b) denote statistical meaningful differences between treatment average values. n.s., there was not significant statistical difference among control and treatment with Rizofos.

Overall, the variables that responded better and with statistical support to the seed treatment with Rizofos^®^ were grain yield (+8%), number of spikes (+5%), and tiller fresh weight (+8%) (Table 2). It is worth mentioning that we did not detect an interaction between the application of fungicide (Compinche^®^) and seed treatment with Rizofos^®^ for any of the variables recorded in the field trials (Supplementary Data 1). In other words, the positive effects of seed inoculation with Rizofos^®^ were not influenced by the application of the fungicide Compinche. With regards to the location of the field trials, the highest yields were registered nearby Pergamino, although there was no interaction between the location and seed inoculation with Rizofos^®^ (Supplementary Data 1).

When the effect of Rizofos^®^ was analyzed for each of the individual field assays by means of a simple ANOVA, we found that in 18 out of 26 cases (70%), the bacterization of seeds significantly increased the yield of wheat with *p* < 0.05 (Supplementary Table 4). If the statistical significance is relaxed at *p* < 0.1, the application of Rizofos^®^ showed a positive yield response in 100% of the 26 field trials (Supplementary Table 4).

In summary, the application to wheat seeds of an inoculant formulated with live *Pseudomonas* sp. strain 1008 showed a robust positive impact on the grain yield in the field across a number of campaigns, soil properties, seed genotypes, and with no significant impact of the simultaneous treatment of the seeds with a fungicide.

### Genomic properties of *Pseudomonas* sp. strain 1008

**FIGURE 3:**
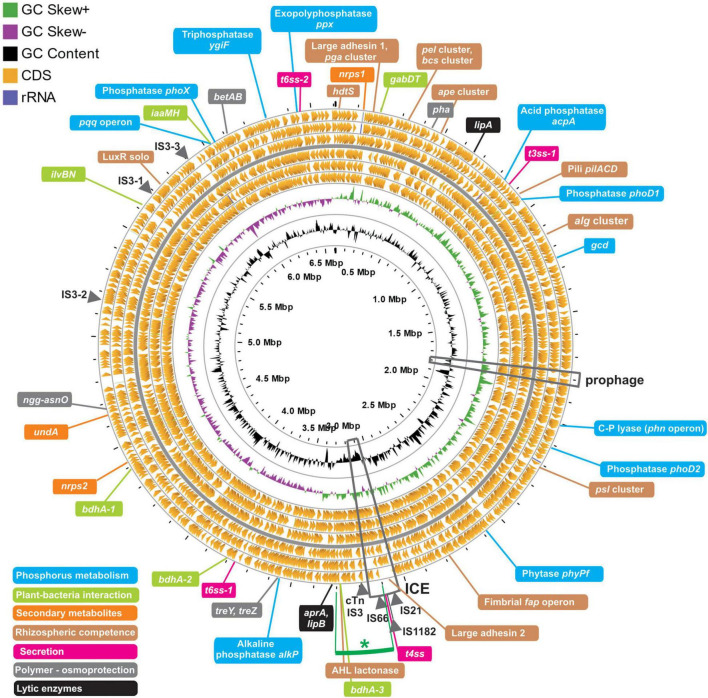
Characteristics and features of the genome of *Pseudomonas* sp. strain 1008. The circular map of the 6.6 Mb chromosome was performed with the GCview server (http://cgview.ca/). The circles represent, from outside to inside: rings 1–6, protein coding genes in the three different reading frames, oriented in the forward (rings 1–3) and reverse (rings 4–6) orientations, respectively. Ring 7 shows GC skews, with positive and negative values being indicated with green and purple colors, respectively. Ring 8 shows G + C% content plot (black). The innermost ring indicates absolute chromosome coordinates. Positions of mobile genetic elements are indicated with arrowheads in the outermost ring. The location of an intact prophage and of an integrative and conjugative element (ICE) are indicated with boxes. Finally, the position of a number of genetic loci related to plant growth promotion and rhizosphere competitiveness are indicated with colored lines, following the reference colors of the legend positioned at the lower left corner.

Prompted by the possibility to uncover the genetic basis for the biostimulant behavior of strain 1008 on the one hand, and to permit a more confident taxonomic positioning of the strain, we determined its genome sequence. The 6.6 Mb genome of this plasmid-free strain is composed by a circular chromosome with a GC content of 60.72% and is predicted to contain 6,000 protein-coding genes, five ribosomal operons and 67 tRNA genes (Table 3 and Figure 3). The size and informational content of strain 1008’ genome is slightly above the average for the genus (6.3 Mb and 5,775 CDS^6^, as of July 2021).

**TABLE 3:** Description of the sequenced, assembled, and annotated whole genome of *Pseudomonas* sp. strain 1008.

Sequencing technology	PacBio RS chemistry sequencing
Polymerase subread bases	1,267,610,607 bp
Total subreads	134,530
Average subread length	9,422 bp
Average reference coverage	192×
Bio-project number	PRJNA741525
NCBI Accession number	CP078013
Genome size	6,609,162 bp
G + C content	60.72%
Chromosome	1
CDS	6,000
Coding density	89%
rRNA operons	5
tRNAs	67
Max. CDS length	14,115 bp
Mean CDS length	980 bp
Genes with COG identified	3,951
Hypothetical proteins (COGs R or S)	34%

One integrative and conjugative element (ICE) of 115 kb is present in the chromosomal region 3,036,538–3,151,403 (Figure 3 and Supplementary Figure 4). It has a GC content of 56.12% (−4.6% with respect to the chromosome average; Table 3 and Supplementary Figure 4) and is flanked by perfect direct repeats of 16 bp at positions 3,036,538 – 3,036,553 (*attL*) and 3,151,388 – 3,151,403 (*attR*). This ICE encodes 91 proteins comprising a likely functional T4SS apparatus with its own relaxase and T4-coupling protein (Supplementary Figure 4). No integrated plasmids were detected, and no *oriT* or relaxase sequences were found outside the ICE region. A search at the Comprehensive Antibiotic Resistance Database using the Resistance Gene Identifier tool revealed four ORFs related to efflux pumps of the RND and MFS families (Supplementary Table 5), typical of pseudomonads (Pasqua et al., 2019). One intact 38 kb-prophage of the *Pseudomonas* phiCTX family (Hayashi et al., 1990) flanked by *attL-attR* sites was identified in between positions 1,778,760 – 1,816,567 (Figure 3). The prophage has a GC content of 57.4% and encodes 41 ORFs (Supplementary Figure 5A). Importantly, the *ctx* gene encoding a eukaryotic cell pore-forming toxin, originally described for the phiCTX phage of *P. aeruginosa* hosts (Hayashi et al., 1990), is not present in the prophage region of strain 1008 (Supplementary Figure 5A). A second but incomplete prophage region is present in positions 3,466,884 – 3,483,655 (16.7 kb; Supplementary Figure 5B), and it most likely represents a phage remnant. Importantly, according to the PathogenFinder tool of the Center for Genomic Epidemiology (Cosentino et al., 2013), the genome of *Pseudomonas* sp. strain 1008 corresponds to a microorganism not pathogenic for humans (Supplementary Figure 6). In support of the latter conclusion, strain 1008 did not show evidence of toxicity toward the nematode *Caenorhabditis elegans* and the springtail *Folsomia candida*, when applied into soil following standardized protocols (Supplementary Data 2, 3). Strain 1008 was neither pathogenic in a murine model following intranasal and oral inoculation of 1 × 10^8^ cells (Supplementary Data 4).

The chromosome of strain 1008 contains minimal orphan CRISPR arrays, three of which seem to be derived from foreign genetic material and two appear to be a self-targeting spacer originated from the own chromosome (Supplementary Table 6). However, no CRISPR-associated (Cas) proteins were detected, so disregarding the functionality of these CRISPR loci.

Finally, a number of transposable elements were identified (Figure 3 and Supplementary Table 7), including single copies of IS66-, IS21-, and IS1182-like elements within the ICE, and one composite transposon bracketed by two IS3 elements located just downstream the ICE. In addition, three copies of IS3 were detected in the rest of the chromosome (Figure 3 and Supplementary Table 7).

The bacterial genome available in public databases with the highest similarity at the DNA sequence level to that of *Pseudomonas* sp. strain 1008 is by large that of *Pseudomonas azotoformans* F77 (GenBank accession CP019856), with an ANIm = 99.08% along a 94.4% of chromosomes alignment. We will deepen on the taxonomical implications of this finding in the next section. As expected, there is a substantial degree of synteny along both chromosomes, except for the fact that strain F77 lacks a large region of about 200 kb in the middle of the replicon, mapping to positions 3,100,000 – 3,300,000 in the chromosome of strain 1008 (Figure 3 and Supplementary Figure 7). This gap in the F77 genome may be partially explained by the insertion of the mobile genetic elements detected in the chromosome of strain 1008 (specifically, the ICE and the composite transposon; Figure 3 and Supplementary Figure 7).

### Genome mining of plant growth-promoting, rhizosphere colonization and competitiveness traits of *Pseudomonas* sp. strain 1008

The complete genome of *Pseudomonas* sp. strain 1008 was annotated with state-of-the-art tools and mined to search for genetic determinants of direct and indirect plant growth-promotion, rhizosphere colonization, and competitiveness (see section “Materials and Methods”). An exhaustive list of the identified genes and operons of interest for this work is presented in Supplementary Table 8, and the physical location of a number of relevant genes/operons is shown in Figure 3.

*Pseudomonas* sp. strain 1008 does not have the genetic capacity to carry out biological nitrogen fixation (Supplementary Table 8), a feature that, within the genus, is only restricted to the species *Pseudomonas stutzeri* (Silby et al., 2011) and to a few number of uncharacterized isolates (Li et al., 2017). Neither it has the genetic determinants for a complete denitrification pathway, only bearing one copy of *napA* encoding the periplasmic nitrate reductase, and the *nirBD* operon encoding subunits of the periplasmic nitrite reductase (Supplementary Table 8). However, the genome of strain 1008 is equipped with a number of genes encoding enzymes that may substantially contribute to the acquisition of phosphorus from extracellular inorganic and organic sources (Figure 3 and Supplementary Table 8). For instance, we identified homologs of *acpA* (acid phosphatase), *alkP* (alkaline phosphatase), *ygiF* (inorganic triphosphatase), *phoD1* and *phoD2* (phosphatases), *phoX* (phosphatase), *ppx* (exopolyphosphatase), the *phn* operon (C-P lyase for organophosphonate utilization), and *phyPf* (phytase of the β-propeller type) (Figure 3 and Supplementary Table 8). Of these, the polypeptides encoded by *phyPf*, *acpA*, *phoD1*, *phoD2*, and *phoX*, contain signal peptides for their export across the plasma membrane (of the Sec/SPI type for the phytase, and of the TAT system for the rest). Additionally, there is one full *pqq* operon encoding enzymes for the biosynthesis of pyrroloquinoline quinone (PQQ). This redox coenzyme is required for the activity of glucose dehydrogenase, an enzyme whose critical role in the solubilization of inorganic phosphates has been demonstrated (De Werra et al., 2009). The corresponding glucose dehydrogenase gene *gcd* was also identified (Figure 3 and Supplementary Table 8).

In pure cultures, strain 1008 produces IAA and this is stimulated by the addition of tryptophan (Supplementary Figure 2). Of the known pathways for the biosynthesis of auxin (Spaepen et al., 2007), we could only detect two genes probably constituting an operon encoding the enzymes for the IAM pathway: namely, the tryptophan-2-monooxygenase IaaM and the indole-3-acetamide hydrolase IaaH (Figure 3 and Supplementary Table 8). On the other hand, we did not find the cluster of genes for IAA catabolism (*iacHABICDEFG*). In addition to IAA, the genome of strain 1008 revealed its genetic potential to produce the volatile compounds acetoin and 2,3-butanediol (Figure 3 and Supplementary Table 8), which have been reported as plant biostimulants and inducers of systemic resistance (Chung et al., 2016). Under stress conditions plants produce and accumulate GABA, but its production has to be controlled because high levels of GABA can impair cell elongation and plant stress resistance (Li et al., 2021). The identification of *gabT* and *gabD* homologs, encoding GABA aminotransferase and succinate-semialdehyde dehydrogenase involved in GABA degradation, and the *gabP* gene, encoding the GABA permease (Figure 3 and Supplementary Table 8), suggests that strain 1008 can acquire and metabolize GABA in the rhizosphere, thus indirectly modulating root GABA levels.

The type three secretion system (T3SS) is a complex protein structure evolutionary derived from the flagellar apparatus that spans the cytoplasmic and outer membranes of bacteria and the cell envelope of the eukaryotic host to deliver effectors directly into its cytosol (Galan et al., 2014). Thus, T3SSs play an important role in plant cell-bacterial interactions, with an outcome of virulent or mutualistic association, essentially depending on the nature of the transported effectors (Zamioudis and Pieterse, 2012). We have detected one T3SS locus of the Hrp-1 type in the genome of strain 1008 (Figure 3 and Supplementary Table 8). The T3SS cluster is highly similar and syntenic to those of the plant-beneficial strains *P. simiae* WCS417 and *P. fluorescens* SBW25 (Supplementary Figure 8). Based on the lack of plant virulence of strain 1008, this T3SS gene cluster -if functional- may be instrumental for induction of systemic resistance and/or modulation of other physiological responses in root cells (Stringlis et al., 2019).

Notably, the genome of strain 1008 lacks biosynthetic gene clusters (BGCs) responsible for the production of antimicrobial compounds that are hallmarks of biocontrol strains of the species *P. protegens*, *P. chlororaphis*, *Pseudomonas donghuensis*, or *P. putida* (namely, 2,4-diacetylphloroglucinol, pyrrolnitrin, pyoluteorin, polyynes, phenazines, hydrogen cyanide, and 7-hydroxytropolone, among others; Supplementary Table 8 and Figure 3). The sole exceptions are the presence of the *undA* gene, encoding an enzyme for the production of the antifungal volatile 1-undecene (Rui et al., 2014), and of a homolog of the *ycaO* gene involved in posttranslational modification of ribosomally synthesized peptides (Burkhart et al., 2017) (Figure 3 and Supplementary Table 8). The number of identified BGCs of the NRPS and PKS type is also remarkably low, just two orphan NRPS clusters and none PKS clusters (Supplementary Table 8). Homologs of the broadly distributed genes in *Pseudomonas* species encoding the extracellular metalloprotease A (*aprA*), the extracellular phospholipase C (*plcN*), and two extracellular lipases (*lipA*, *lipB*) were identified. By contrast, no chitinase gene was detected. Strain 1008 neither possesses genes for characterized insecticidal proteins (Kupferschmied et al., 2013).

In terms of iron scavenging, we identified the *pvd* gene cluster for the biosynthesis of pyoverdine. In fact, spectroscopic signals in the UV-visible range typical of pyoverdine were detected in supernatants from cultures of strain 1008 in iron-deficient medium (Supplementary Figure 2G). Besides the pyoverdine gene cluster, we found genes encoding the hemophore protein HasA and the corresponding hemophore receptor. Interestingly, we did not detect the genes for biosynthesis of the siderophore achromobactin, but we identified one gene encoding a putative achromobactin receptor (Supplementary Table 8). No other genetic determinants for the production of additional siderophores were found. With regards to the transport of iron complexes, the genome of strain 1008 bears five genes encoding putative ferric-pyoverdine receptors and 13 genes for TonB-dependent receptors (six of which are located adjacent to iron-metabolism genes; Supplementary Table 8).

Effective colonization of the rhizosphere is key to execute direct and indirect mechanisms of plant growth-promotion (Lugtenberg and Kamilova, 2009). Our current studies on the root colonizing ability of strain 1008 point to a strong competitiveness to access the rhizoplane of wheat in natural soil in the presence of indigenous microorganisms and irrespective of its initial location (bulk soil or seeds), although the survival of strain 1008 in bulk soil is severely limited in the absence of plant roots (unpublished data). The abilities to adhere to surfaces and establish biofilms, as well as to compete with other rhizobacteria, are critical factors to colonize the rhizoplane. In this sense, the genome of strain 1008 is endowed with the genetic capacity to produce polysaccharides commonly found in the extracellular matrix of biofilms (e.g., alginate, Psl, and Pel), as well as for the biosynthesis of a set of macromolecules mediating surface adhesion (cellulose, PGA adhesin, Fap amyloid fimbriae, and two large adhesins of the ShlA/HecA/FhaA family) (Figure 3 and Supplementary Table 8). The rhizosphere niche colonization by strain 1008 may be facilitated by its swimming motility driven by the flagellar apparatus (Figure 1) encoded by the flagellar operons, as well as by twitching motility associated with pili (Supplementary Table 8).

Strain 1008 lacks canonical *luxIR* genes for AHL production and global coordination of gene expression according to cell density, but it possesses one AHL synthase gene of the *hdtS* type, one LuxR-solo gene, and one AHL-lactonase encoded within the ICE region (Figure 3 and Supplementary Table 8). This implies that strain 1008 would be able to respond to its own and/or to foreign AHLs, and/or to interfere with cell–cell communication of other rhizobacteria. Finally, we identified genes for production of metabolites that confer protection to cells against different types of stress, namely osmotic stress [*betAB*, *ngg-asnO*, and *treY*/*treZ*, for betaine, NAGGN and trehalose biosynthesis, respectively; (Kurz et al., 2010)], oxidative stress [*ape* cluster for aryl polyene production; (Schoner et al., 2016)], and general stress [*pha* genes for polyhydroxyalkanoates; (Lopez et al., 2015)] (Figure 3 and Supplementary Table 8). Collectively, the expression of these genes may confer increased fitness in the rhizosphere to strain 1008.

An interesting bacterial strategy to compete with non-kin cells is the type VI secretion system (T6SS), a contractile nanoweapon to inject toxins directly into the cell membranes, periplasm, or cytoplasm, leading to killing of competitor cells if they do not have the matching immunity protein (Ryu, 2015). The injected T6SS effectors have different biochemical activities, but the nanomachine and their injected toxins are not harmful for plant cells (Lucke et al., 2020). The genome of strain 1008 contains two complete T6SS loci, and a number of potential effector toxins of the *tse5* (four copies) and *vrgG* (three copies) types (Figure 3 and Supplementary Figure 9 and Supplementary Table 8). Besides the genetic determinants of the T6SS nanomachines, strain 1008 contains genes encoding bacteriocins (Ghequire and De Mot, 2014; Jackson et al., 2018): we identified functional homologs of S-pyocins of the *pys1* (for a colicin-like rRNAse) and PA3865 (for a tRNAse) types, as well as two copies of putative CDI toxins related to PA0041 and PA2462 proteins from *P. aeruginosa*, and three copies of a gene encoding a putative bacteriocin of the DUF692 family. It would be interesting to evaluate the functionality of the two detected T6SS loci in standard bioassays for antibacterial activity.

The broadly conserved global regulatory cascade of *Pseudomonas* species known as Gac-Rsm is fundamental to coordinate different traits for colonization of plant tissues, production of secondary metabolites and the interaction with eukaryotic organisms sharing the niche with pseudomonads (Ferreiro et al., 2018; Sobrero and Valverde, 2020). The genome of strain 1008 contains all the essential and accessory genetic elements delineating a functional Gac-Rsm cascade, including the GacS-GacA two-component system, the GacS-modulating histidine kinases RetS, LadS, and PA1611-like, three ortholog RNA binding proteins of the Rsm family (RsmA, RsmE, and RsmI), and the two cognate non-coding regulatory RNAs RsmY and RsmZ (Supplementary Table 8).

### Phylogenomic, phenotypic and chemotaxonomic comparative analysis of *Pseudomonas* strain 1008 and related taxa

The first attempt to assign a taxonomic position to *Pseudomonas* sp. strain 1008 by comparative analysis of a 1,325 bp partial sequence of the PCR amplified 16S rDNA gene indicated that the isolate had >98.4% of sequence identity with several species belonging to the *P. fluorescens* subgroup within the *P. fluorescens* lineage (Table 4 and Supplementary Figure 1). For a more detailed analysis of its phylogenetic positioning, we drew on the complete genome sequence of strain 1008. A pairwise genome comparison with a set of type species having highly similar 16S rDNA sequences revealed that the closest taxonomic species was *Pseudomonas lurida* (Table 4). However, the calculated ANIb and ANIm values for strain 1008 were far below the current accepted threshold of 95% for species definition (Jain et al., 2018). In addition, the dDDH values calculated for each close relative species with respect to strain 1008, were all below 70% (Table 4), the cutoff for delimiting species (Meier-Kolthoff et al., 2013). These findings firmly suggested that strain 1008 may be a representative of a novel *Pseudomonas* species within the *P. fluorescens* subgroup.

**TABLE 4:** Overall genome relatedness indices derived from pairwise genomic comparison between *Pseudomonas* sp. strain 1008 and type species with the highest similarity of 16S rDNA sequences.

Type strain	16S^1^	ANIb (%)^2^	ANIm (%)^3^	dDDH^4^
*P. lurida* LMG 21995	99.02	90.30 (79.9)	91.73 (79.8)	67.6 (64.2 – 70.8)
*P. marginalis* pv. *marginalis* ICMP 3553	98.79	90.02 (76.7)	91.41 (77.8)	61.6 (58.3 - 64.8)
*P. extremorientalis* LMG 19695	98.57	89.92 (79.5)	91.22 (80.0)	66.9 (63.5 – 70.1)
*P. azotoformans* LMG 21611	99.85	89.88 (81.3)	91.22 (91.9)	66.6 (63.2 – 69.8)
*P. simiae*_CCUG_50988	98.91	89.51 (77.2)	90.96 (77.2)	62.5 (59.2 – 65.7)
*P. veronii* DSM 11331	98.42	86.49 (69.7)	88.87 (66.5)	n.d.
*P. salomonii* LMG 22120	99.09	86.34 (74.4)	88.59 (71.1)	n.d.
*P. fluorescens* ATCC 13525	98.79	86.11 (72.9)	88.45 (69.0)	n.d.
*P. trivialis*_LMG_21464	98.87	85.45 (62.8)	88.33 (58.8)	n.d.

^1^% identity along a 1,325 bp amplicon sequence from Pseudomonas sp. strain 1008, based on BlastN. ^2^Pairwise average nucleotide identity calculation based on Blast (ANIb) with the % of aligned genome sequence in between parentheses. ^3^Pairwise average nucleotide identity calculation based on MUMmer (ANIm) with the % of aligned genome sequence in between parentheses. ^4^Digital DNA–DNA hybridization (dDDH) estimate values (%) based on in silico DDH according to formula d_6_ [a.k.a. GGDC formula 3, TYGS server; (Meier-Kolthoff et al., 2013; Meier-Kolthoff and Göker, 2019)]; model-based confidence intervals are specified in between parentheses. Shaded cells indicate the highest scores for every genomic feature. n.d., not determined.

**FIGURE 4:**
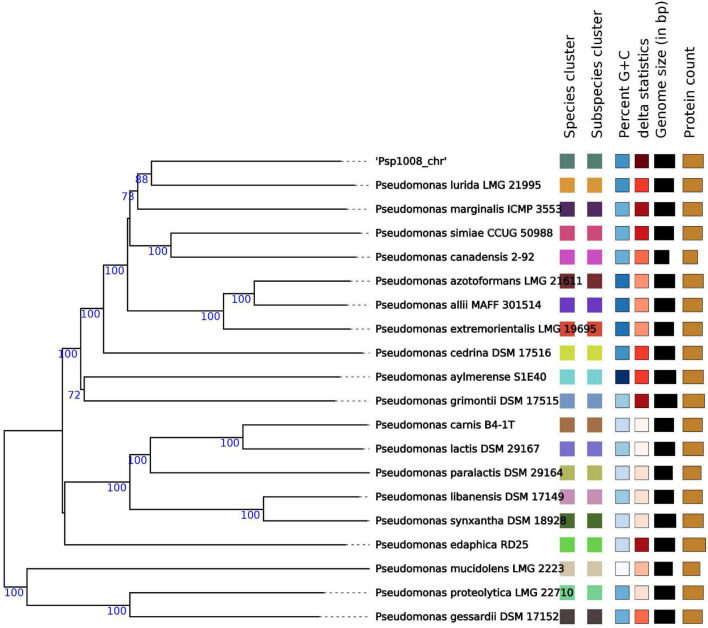
Genome BLAST Distance Phylogeny (GBDP) tree of *Pseudomonas* sp. strain 1008. The phylogenetic tree was constructed with the type (Strain) Genome Server (Meier-Kolthoff and Göker, 2019), which produces a GBDP tree by approximating intergenomic relatedness using the MASH algorithm among all type strain genomes in the TYGS database and by extracting and comparing 16S rRNA gene sequences with >14.990 type strains using BLAST as a proxy to identify the 50 closest type strains to calculate precise distances. The tree itself was constructed using FastME version 2.1.4 to infer a balanced minimum evolution tree with branch support. The tree represents only the *Pseudomonas* spp. most closely related to strain 1008. Bootstrap support values are shown at the nodes. The leaf label “Species cluster” assigns a different color to denote genomes from different type species.

To get further support for this hypothesis we profited on the genome-based taxonomy service provided by the DSMZ Type (Strain) Genome Server (TYGS; Meier-Kolthoff and Göker, 2019). In agreement with the ANI similarity and dDDH data (Table 4), the TYGS analysis reinforced the finding that strain 1008 is a representative of a novel species of *Pseudomonas* (Figure 4 and Supplementary Figure 8).

**FIGURE 5:**
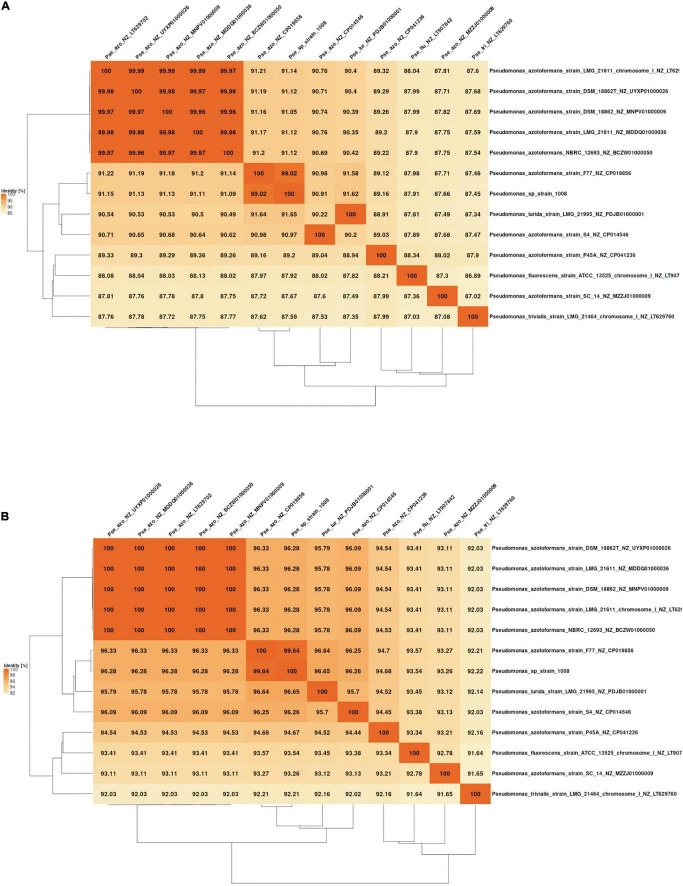
Overall genome relatedness derived from pairwise genomic comparison between *Pseudomonas* sp. strain 1008 and type species and isolates with the highest similarity of 16S rDNA sequences. **(A)** Average nucleotide identity matrix generated with the fastANI matrix tool of the EDGAR platform (https://edgar.computational.bio.uni-giessen.de/cgi-bin/edgar_login.cgi). **(B)** Average amino acid identity matrix generated with the AAI matrix tool of the EDGAR platform (https://edgar3.computational.bio.uni-giessen.de/cgi-bin/edgar.cgi).

When the relatedness of the genome of strain 1008 was analyzed by comparison to all *Pseudomonas* genomes available in public databases, we found that the highest similarity indexes corresponded to the genome of *P. azotoformans* strain F77 (NCBI accession CP019856), with ANIb and AAI (average amino acid identity) values of 99.02 and 99.64, respectively (Figure 5). However, the genomic similarity between the type strain of the *P. azotoformans* species (LMG 21611) and strain 1008 (Table 4 and Figures 4, 5) is not consistent with this finding, thus raising doubts about the taxonomical positioning of *P. azotoformans* F77. In fact, the ANIm and AAI analyses of all available genomes of isolates designated as members of the *P. azotoformans* species (including that of the species type strain), clearly support the fact that both strains 1008 and F77 are representatives of a novel species distinct from *P. azotoformans* and from closely related type species (Figures 4, 5). In addition to the evidence provided by genomic metrics, the comparison of the composition of whole-cell fatty acids and of key biochemical activities between strain 1008 and closely related taxa (Tables 5, 6), also provide phenotypic support for the separate species status that was shown by the phylogenomic analysis.

**TABLE 5:** Comparison of the relative abundance (%) of major cellular fatty acids of *Pseudomonas* sp. strain 1008 and its closest phylogenetic relatives (see Table 4 and Figure 4).

Fatty acid	16:0	16:1 w7c	18:1 w7c	17:0 cyclo w7c	12:0 2OH	12:0 3OH
*Pseudomonas* sp. 1008	30.4	29.5	15.3	9.1	4.3	3.7
*P. lurida* LMG 21995	n.r.	n.r.	n.r.	n.r.	n.r.	n.r.
*P. marginalis*_ICMP_3553	32.4	26.6	14.8	7.5	n.r.	n.r.
*P. extremorientalis* LMG 19695	37.0	8.7	14.0	31.3	n.r.	n.r.
*P. azotoformans* LMG 21611	31.1	32.0	17.2	3.6	3.7	4.0
*P. simiae* OLi^T^	30.3	27.0	12.3	11.7	n.r.	n.r.

Values were obtained from Campos et al. (2010) for P. marginalis ICMP 3553T, (Ivanova et al., 2002) for P. extremorientalis LMG 19695, (Sawada et al., 2021) for P. azotoformans LMG 21611, and (Vela et al., 2006) for P. simiae OLi^T^. Values for P. lurida LMG 21995 could not be found in the literature. Cellular fatty acids analysis of strain 1008 was carried out by DSMZ Services, at the Leibniz-Institut DSMZ (Germany). n.r., not reported in the literature.

**TABLE 6:** Biochemical features differentiating *Pseudomonas* sp. strain 1008 from the type strains of closely related species (see Table 4 and Figure 4).

	Strain 1008	*P. lurida*	*P. orientalis*	*P. extremorientalis*	*P. azotoformans*	*P. simiae*
Nitrate reduction	+w	−	−	−	+	+
Gelatinase	−	+	+	+	+	+
Urease	−	n.r.	+	−	−	−
Arginine dehydrolase	−	+	+	+	+	+
L-rhamnose assimilation	+w	+	+	−	−	−
*N*-acetyl-D-glucosamine assimilation	+	+	+	+	+	−

Data were retrieved from BacDive (https://bacdive.dsmz.de/) and from the literature (Dabboussi et al., 1999; Behrendt et al., 2007; Von Neubeck et al., 2017; Lick et al., 2020). +, positive reaction; +w, weak positive reaction; −, negative. n.r., not reported.

Based on the evidence described above demonstrating that strain 1008 represents a newly derived branch in the phylogenomic tree of the *P. fluorescens* lineage (Figures 4, 5), we propose a new taxonomic entity with the name “*Pseudomonas pergaminensis*” sp. nov.

### Description of *Pseudomonas pergaminensis* sp. nov.

*Pseudomonas pergaminensis* (per ɣa mi ′nen sis), N.L. masc./fem. adj. pergaminensis, belonging to Pergamino county (Province of Buenos Aires, Argentina), from where the type strain was isolated.

Cells of strain 1008^T^ are Gram-reaction negative, aerobic, oxidase positive, rod-shaped bacteria ranging between 0.6–1.1 mm wide × 1.2–3.2 μm long, non-endospore-forming rods. Grows on *Pseudomonas* agar F at 28°C forming circular colonies <2 mm, with smooth borders, opaque, of light beige color, and creamy texture. It may give rise to phase-variant colonies. Growth occurs at 4–35°C but not at 37°C. Strain 1008^T^ grows in a pH range from 6–9, and with a NaCl concentration of up to 1% w/v. Results from tests using API 20 NE Gallery System show that the following substrates are utilized: D-glucose, D-arabinose, D-mannose, D-mannitol, *N*-acetyl-D-glucosamine, D-gluconate, capric acid, adipic acid, malic acid, and citrate. Using the Biolog GenIII system, tests positive for the utilization of the following additional substrates: dextrin, D-fructose, D-fructose-6-phosphate, D-galactose, D-fucose, L-fucose, L-rhamnose, L-galactonic acid lactone, D-galacturonic acid, D-glucuronic acid, glucuronamide, acetic acid, quinic acid, γ-amino butyric acid, formic acid, and Tween-40 (Supplementary Data 5 and Supplementary Figure 10). The major cellular fatty acids are C_16:0_ (30.4%), C_16:1 *w*7*c*_ (29.5%), C_18:1 *w*7*c*_ (15.3%), C_17:0 *cyclow*7*c*_ (9.1%), C_12:0 2*OH*_ (4.3%), C_12:0 3*OH*_ (3.7%), C_10:0 3*OH*_ (3.0%), and C_12:0_ (2.3%) (Supplementary Data 6). It is able to solubilize mineral phosphates and zinc oxide, to produce IAA and siderophores, to secrete proteases and phospholipases, but not chitinases. The strain 1008^T^ was isolated form the rhizosphere of field grown *Triticum aestivum*, in Pergamino, Buenos Aires province, Argentina, in 2003. The genomic DNA G + C content of the type strain is 60.72%. The full genome sequence of the strain 1008^T^ has been deposited at the NCBI GenBank under accession number CP078013. The type strain is 1008^T^ (= DSM 113453 = ATCC TSD-287).

## Discussion

The urgent need of sustainable and eco-friendly strategies to mitigate the impact of agrochemical industry and its massive application has boosted research on plant-biostimulant microorganisms, whereas at the same time, it has exposed frequent inconsistencies between the plant-beneficial potential of isolated microorganisms studied under lab conditions and their efficiency in the field (Timmusk et al., 2017; Backer et al., 2018; Basu et al., 2021). Such hurdles may be partially reduced if the microorganisms are isolated from plant tissues of the target crop and are applied in the geographical area of origin (Armada et al., 2018; Müller and Behrendt, 2021). *Pseudomonas* sp. strain 1008 was isolated from the rhizosphere of healthy field-grown wheat plants, as a bacterium able to increase the availability of phosphate to plant roots and to produce auxin (Figure 1 and Supplementary Figure 2). Based on these traits, strain 1008 was formulated as an inoculant for its application on wheat seeds before sowing and tested in a series of field trials at three different locations around the isolation site, across several campaigns involving different wheat varieties (Figure 1a and Supplementary Tables 1, 2, 4). Collectively, the results of the whole set of field trials strongly support the use of this biostimulant bacterium (Du Jardin, 2015) as an efficient agricultural input for promoting the yield of wheat (Table 2 and Supplementary Table 4). The fact that a single seed bacterizing shot with live *Pseudomonas* sp. strain 1008 resulted in an average increase of wheat yield of 8% (median = +9.7%; Table 2 and Supplementary Table 4), suggests that seedling establishment and its early development are critical stages that are strongly responsive to the presence of the bacterium and its physiological activities, and whose beneficial effects are largely transduced all along the growth cycle of the plant. Recently, it has been proposed that domestication and breeding strategies have worked against the maintenance of plant genes promoting interactions with plant-beneficial bacteria (Valente et al., 2020). In this regard, bacterization of commercial wheat seeds with strain 1008 may represent a way to by reinstate key microbial functions in the rhizosphere of seedlings by compensating the reduced ability of modern wheat varieties to interact with PGPR (Perez-Jaramillo et al., 2016; Valente et al., 2020).

### Physiological and genomic plant-beneficial traits of *Pseudomonas* sp. strain 1008

*In vitro*, strain 1008 was capable of solubilizing phosphate from pure calcium triphosphate and from mineral phosphate rocks, mineralizing phosphate from organic esters, solubilizing zinc from ZnO, producing siderophores and extracellular lytic enzymes of the phospholipase and protease types (Table 1 and Supplementary Figure 2). All these features may cooperatively contribute to enhancing nutrient availability in the rhizosphere of wheat plants.

The closest related strain to *Pseudomonas* sp. 1008 described in the literature is the isolate F77 (Supplementary Figures 7, 8). Strain F77 was originally assigned to the *P. azotoformans* species, but on the basis of our phylogenomic analysis this species ascription should be revised (Figure 5). Strain F77 was reported as a strong biomineralizer of aluminum and iron from biotite minerals, being this feature fully dependent on the activity of the product of the *gcd* gene, encoding the periplasmic glucose dehydrogenase enzyme (Wang et al., 2020). In pseudomonads, glucose dehydrogenase drives the oxidation of glucose into gluconic acid in a reaction that requires the PQQ cofactor (Miller et al., 2010). Gluconic acid may be further oxidized in the periplasm to 2-ketogluconic acid by the activity of the product of the *gad* gene (gluconic acid dehydrogenase) (Miller et al., 2010). This pathway of glucose uptake leads to acidification of the cell environment, which indirectly facilitates solubilization of mineral phosphates. In fact, genetic data confirmed the absolute requirement of *gcd*, *gad* and *pqq* genes for solubilization of calcium triphosphate by the biocontrol rhizospheric strains *P. fluorescens* F113 and *P. protegens* CHA0 (De Werra et al., 2009; Miller et al., 2010). Gcd can also oxidize xylose (Dvorak and De Lorenzo, 2018), another abundant sugar present in plant root exudates. The genome of strain 1008 encodes *gcd*, *gad*, and *pqq* homologs (Figure 3 and Supplementary Table 8), thus providing genomic support for the observed capacity to solubilize mineral phosphates. Acidification of the extracellular environment in the rhizosphere has been found to contribute to local suppression of root immunity upon recognition of bacterial inducers of ISR, an effect that for certain rhizospheric *Pseudomonas* species required PQQ biosynthesis and gluconic acid production (Yu et al., 2019). Thus, root colonization by strain 1008 may also be promoted through this mechanism of ISR modulation associated with the control of extracellular pH driven by PQQ-dependent acidification (Yu et al., 2019), which in parallel contributes to solubilization of mineral phosphates.

Additionally, several genes of strain 1008 encode phosphatases acting on organic phosphate esters, some of which are predicted to be secreted (i.e., a β-propeller-phytase, the acid phosphatase AcpA, and the phosphatases PhoD1, PhoD2, and PhoX). Notably, strain 1008 has an *alkP* homolog, encoding an alkaline phosphatase, that is also present in only two out of the 613 complete genomes available in the PseudoDB database: the phylogenetically closest isolate “*P. azotoformans*” strain F77 and *Pseudomonas extremorientalis* BS2774, the latter being a member of a subgroup whose type species is also closely related to strain 1008 (Table 4 and Figure 4). Altogether, the repertoire of genes related to the metabolism of inorganic and organic sources of phosphates present in the genome of strain 1008 (Figure 3 and Supplementary Table 8) suggests its potential as a phosphorus biofertilizer in the rhizosphere of wheat.

*Pseudomonas* sp. strain 1008 produces auxin in a tryptophan-dependent manner (Table 1 and Supplementary Figure 2). In bacteria, five different tryptophan-dependent pathways for IAA biosynthesis have been reported (Spaepen et al., 2007). By contrast, the genetic basis for the tryptophan-independent production of IAA is unknown (Spaepen et al., 2007). We could not identify the *ipdC* (IPA pathway), *oxd* and *nha1* (IAOc/IAN pathway), and *tdc* (TPM pathway) genes in the genome of strain 1008. We detected, however, a putative operon highly resembling the *iaaMH* tandem of the IAM pathway (Figure 3 and Supplementary Table 8). Given that the gene responsible for the fifth biosynthetic pathway involving a tryptophan side-chain oxidase (TSO) is yet unknown (Spaepen et al., 2007), we cannot rule out the presence of a *tso* homolog in the genome of strain 1008. Besides auxin, the genome of strain 1008 has the potential to produce other phytostimulators, such as the volatiles 2,3-butanediol and acetoin [two compounds known to be involved in plant growth stimulation (Ryu et al., 2004; Chung et al., 2016)], and the cofactor PQQ, which in addition to its role in phosphate solubilization, it has been reported to act a phytostimulator with antioxidant properties in tomato and cucumber (Choi et al., 2008). With regards to modulation of plant hormone homeostasis during abiotic stress responses, the genome of strain 1008 does not encode a *bona fide* ACC deaminase gene *acdS*, but it encodes the GABA permease GabP and the GABA catabolic enzymes GabD-GabT (Figure 3 and Supplementary Table 8). Thus, strain 1008 may contribute to attenuate the GABA-mediated response to abiotic and biotic stress (Li et al., 2021) in the rhizosphere of colonized plants, in a way comparable with bacterial ACC deaminase to limit ethylene production by plant tissues (Gamalero and Glick, 2015). All these genetic traits directly related to phytoregulation suggest that cells of *Pseudomonas* sp. strain 1008 colonizing the rhizosphere of wheat may have a key role in promoting root system development locally and contributing systemically to modulation of plant responses to stresses.

With regards to indirect mechanisms of plant growth promotion, strain 1008 was unable to produce HCN [a volatile compound responsible for the inhibition of diverse phytopathogens (Blumer and Haas, 2000)] and to antagonize several phytopathogenic fungi and one oomycete *in vitro* (Table 1 and Supplementary Figure 2). These findings provide stronger support toward a direct plant biostimulatory effect, rather than to an indirect biocontrol-based mechanism, as the main contribution of strain 1008 to the promotion of wheat growth and yield in the field. In this regard, mining of the full genome sequence of strain 1008 clearly indicated that this isolate was not evolved to fight pathogens with secondary metabolites typical of biocontrol PGPR, like antibiotics, antimetabolites, and lipopeptides of NRPS origin (Figure 3 and Supplementary Table 8) (Keswani et al., 2020). However, the capacity to induce systemic resistance cannot be discarded for strain 1008, as typical general elicitors of ISR like LPS, flagella and siderophores of the pyoverdine type (Pieterse et al., 2014) are structural and functional components of strain 1008 (Figure 1, Supplementary Figure 2, and Supplementary Table 8). Furthermore, the genome of strain 1008 encodes genes for the production of the volatile compounds 2,3-butanediol and acetoin (Figure 3 and Supplementary Table 8), which are also elicitors of ISR (Pieterse et al., 2014; Silva Dias et al., 2021). In addition, it has been recently proposed that certain plant-beneficial rhizobacteria may engage into a feedback loop. The recognition of key MAMPs by the plant immune system triggers a defense reaction that stimulates bacterial production of IAA, thus reinforcing development of lateral roots and ISR itself on the plant side, but also promoting root colonization by the rhizobacterium (Tzipilevich et al., 2021). It would be interesting to explore if the IAA-producer strain 1008 can also enter into such a virtuous loop with plant roots.

The compendium of genes and operons detected in the genome of *Pseudomonas* sp. strain 1008 that are potentially involved in biofertilization and phytostimulation as the main underlying mechanisms for promotion of wheat yield in the field, as well as those genetic determinants potentially relevant for rhizosphere colonization and competitiveness (Figure 3 and Supplementary Table 8), set the basis for designing functional genetics screens and gene expression profiling of strain 1008, in order to provide experimental support linking the functional relevance of the cataloged genetic repertoire of plant-beneficial traits in the rhizosphere. An important issue that has to be considered when aiming to link gene functions to PGPR mechanisms and, by transition, to agronomic efficiency in the field, is that plant-beneficial rhizospheric microbes may display a set of concurring mechanisms that contribute to the overall robust performance in the field, as it has been addressed for the agronomic response of different crops to the inoculation with *Azospirillum brasilense* [i.e., the “multiple mechanisms hypothesis” (Bashan and De-Bashan, 2010; Cassán et al., 2020)]. This hypothesis may explain the overall positive impact of strain 1008 on the yield of wheat across a number of field assays carried out in different locations, with different seed genotypes and across different campaigns (Figure 2 and Supplementary Table 2). Under this scenario, we have preliminary data indicating that strain 1008 promotes early root development of wheat in natural soil, and that induces root branching and expression of auxin-responsive reporter fusions in *Arabidopsis thaliana* (unpublished data). These effects would be consistent with the observed production of auxin by strain 1008 (Supplementary Figure 2) and with the identification of an operon encoding the auxin biosynthetic proteins IaaM-IaaH of the IAM pathway (Figure 3 and Supplementary Table 8); together, these clues would point to auxin production in the rhizosphere as one of the possible mechanisms of strain 1008 contributing to the promotion of root development in the field. However, and importantly, such hypothesis needs to be challenged with targeted mutation of the *iaaMH* genes; the same reasoning applies to all other genetic traits with potential relevance in the field that were identified in the genome of strain 1008 (Figure 3 and Supplementary Table 8).

### Strain 1008 is a representative of the proposed novel species *Pseudomonas pergaminensis*

The initial taxonomic assignment of strain 1008 indicated that the isolate belonged to the *P. fluorescens* lineage, with its 16S rDNA sequence being 98.4–99.1% similar to those of different type species within this large and complex cluster of the *Pseudomonas* genus (Table 4 and Supplementary Figure 1). On the basis of the full genome sequence, we concluded that isolate 1008 represents a novel species of the genus *Pseudomonas* (Figure 4 and Supplementary Figure 8). Moreover, our genomic comparative analysis led us to conclude that this novel proposed species for strain 1008, must also include the aluminum- and iron-weathering isolate F77, which had been originally typed as a *P. azotoformans* isolate (Wang et al., 2020), and that shares an overall genomic relatedness with strain 1008 of 99.08% at the nucleotide level, and of 99.64% at the amino acid level (Figure 5). The phenotypic and chemotaxonomic features of strain 1008 confirmed its differentiation from related taxa (Tables 5, 6, Supplementary Datas 5, 6, and Supplementary Figure 10). The results from this polyphasic approach support the classification of 1008^T^ as a novel species of *Pseudomonas*, and the name of *Pseudomonas pergaminensis* is thus proposed for this strain.

## Conclusion

We report the isolation, physiological characterization, and genomic analysis of a wheat rhizospheric strain representative of a novel species of the genus *Pseudomonas*, that showed a robust positive effect on the yield of field-grown wheat upon its application as a formulated inoculant on seeds. *In vitro* and genomic traits strongly suggest that *Pseudomonas pergaminensis* 1008^T^ acts as a plant biostimulant, rather than as a biocontrol bacterium. Full genome information will be useful for functional characterization of the mechanisms underlying the robust performance of strain 1008 in the field.

## Data availability statement

The datasets presented in this study can be found in online repositories. The names of the repository/repositories and accession number(s) can be found in the article/Supplementary Material.

## Author contributions

MD and TB isolated, characterized, and developed the commercial formulation of strain 1008. FN designed, implemented, and monitored field trials. GGA and WC supervised research and the agronomical evaluation of the commercial formulation. BA performed the statistical analysis of agronomical data and prepared DNA for genome sequencing. DW carried out genome assembly, annotation, and a set of comparative genomic tests. CV carried out genome mining, phylogenomic analysis, organized datasets, elaborated article structure, and wrote the manuscript. All authors contributed to manuscript revision, read, and approved the submitted version.

## Footnotes

1
https://imagej.nih.gov/ij/

2
http://www.suelo-fertil.com.ar

3
https://www.ncbi.nlm.nih.gov/tools/vecscreen/

4
https://card.mcmaster.ca/analyze/rgi

5
https://blast.ncbi.nlm.nih.gov/Blast.cgi

6
www.pseudomonas.com
