# Supplementary material for: Agronomic efficiency and genome mining analysis of the wheat-biostimulant rhizospheric bacterium Pseudomonas pergaminensis sp. nov. strain 1008T
Source: Front Plant Sci. 2022 Jul 28;13:894985. doi: 10.3389/fpls.2022.894985 (PMC9369656; doi:10.3389/fpls.2022.894985)
Supplement: Supplementary file 1 [file Data_Sheet_1.PDF]

Supplementary Data 1

Statistical analysis of field assays

The dataset involves 25 field assays with the following conditions:

- 7 campaigns carried out mainly in 2 locations (only the 2014/1015 campaign was carried out in 3 locations).
- 5 wheat varieties.
- 2 forecrops (soybean, or wheat/soybean).
- 2 pre-treatment of seeds (with the fungicide Compinche®, or no fungicide).
- Agricultural management: no-till.

In all 25 field assays, the following variables were recorded, and considered for the multivariate analysis:

- Grain yield (kg/ha).
- Number of plants per m^2.
- Number of spikes per m^2.

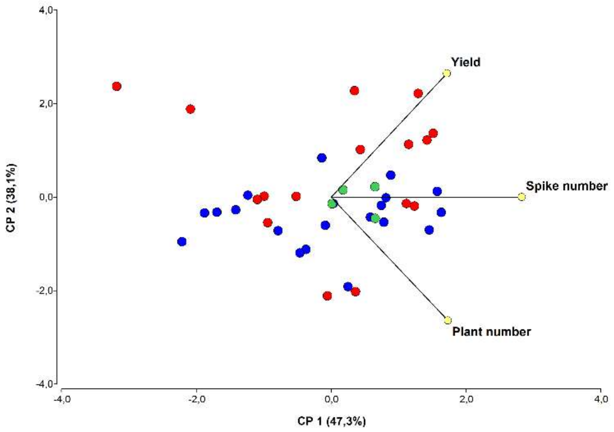

Principal component analysis of crop parameters measured for wheat in 25 field assays (see Supplementary Table 2 - Raw data of field assays.xlsx). Symbols for locations: red, Pergamino; blue, Ferré; green, Junín. Classifiers: location, campaign, variety, fungicide treatment, forecrop, seed bacterization. CP1 and CP2 explain more than 90% of the total variance.

No single factor within the classifiers showed a general effect. Data are grouped in pairs "bacterized/non-bacterized seeds". This is even more evident if the classifiers are only location and seed bacterization:

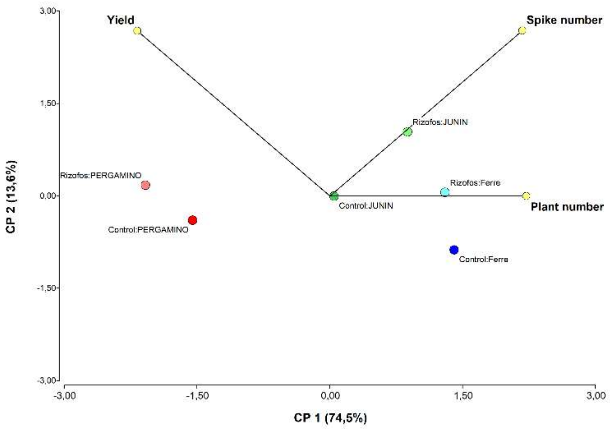

The effect of seed bacterization is clear, and mainly dependent on the contribution of grain yield and number of spikes per m^2. The effect of location is evident on the number of plants per m^2.

If we only consider the influence of seed bacterization in a PCA, the PC1 explains 100% of the data variability, and the field plots where wheat seeds were bacterized showed the highest values for the three variables measured in all trials (grain yield, number of plants per m^2, and number of spikes per m^2):

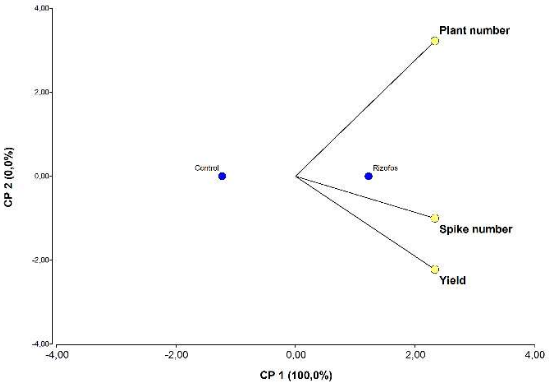

Based on the observation that the rest of the variables had a minor contribution to data variability, we applied a Generalized Linear Mixed Model in which all variables (except for seed bacterization) were considered as random effects.

The model with the best fit (*i.e.*, with the lowest values of AIC and BIC) was:

<u>Fixed effect</u>: seed bacterization (+Rizofos®, control)
<u>Random effects</u>: forecrop, location, campaign, fungicide application [all affecting the Constant]; seed variety [affecting seed bacterization]
All variables with adjusted variance (VarIdent), individually.
VarPower fitted to ensure homocedasticity.

Grain yield

Model fitting values

N	AIC	BIC	logLik	Sigma	R2 0	R2 1	R2 2	R2 3	R2 4	R2 5
150	2157,30	2232,23	-1053,65	1,2E-10	0,04	0,05	0,05	0,05	0,89	0,89

Marginal hypotheses tests (SC type III)

	numDF	denDF	F-value	p-value
(Intercept)	1	129	220,90	<0,0001
Treatment	1	129	18,32	<0,0001

Adjusted average and standard errors for *Treatment*

LSD Fisher (Alpha=0,05)

p-values adjustment procedure: No

Treatment	Average	S.E.	
Rizofos	4423,79	288,96	**A**
Control	4084,38	288,95	**B**

Average values followed by the same letter are not statistically different (p > 0,05)

Number of plants per m²

Model fitting values

N	AIC	BIC	logLik	Sigma	R2 0	R2 1	R2 2	R2 3	R2 4	R2 5
150	1417,83	1492,76	-683,92	674303,51	1,1E-03	1,1E-03	1,1E-03	1,1E-03	1,1E-0	0,60

Marginal hypotheses tests (SC type III)

	numDF	denDF	F-value	p-value
(Intercept)	1	129	2845,91	<0,0001
Treatment	1	129	1,76	0,1874

Number of spikes per m²

Model fitting values

N	AIC	BIC	logLik	Sigma	R2 0	R2 1	R2 2	R2 3	R2 4	R2 5
150	1622,32	1697,25	-786,16	5,92	2,7E-03	0,01	0,67	0,67	0,84	0,84

Marginal hypotheses tests (SC type III)

	numDF	denDF	F-value	p-value
(Intercept)	1	129	104,55	<0,0001
Treatment	1	129	4,98	0,0274

Adjusted average and standard errors for *Treatment*

LSD Fisher (Alpha=0,05)

p-values adjustment procedure: No

Treatment	Average	S.E.	
Rizofos	388,34	37,30	**A**
Control	371,35	37,38	**B**

Average values followed by the same letter are not statistically different (p > 0,05)

From here below, the GLMM fitting data correspond to parameters that were determined for a subset of the total field assays (see Supplementary Table 2 - Raw data of field assays.xlsx).

Tiller number

Model fitting values

N	AIC	BIC	logLik	Sigma	R2 0	R2 1	R2 2	R2 3	R2 4	R2 5
114	1277,86	1343,10	-614,93	0,01	2,4E-05	0,01	0,84	0,90	0,91	0,94

Marginal hypotheses tests (SC type III)

	numDF	denDF	F-value	p-value
(Intercept)	1	98	98,64	<0,0001
Treatment	1	98	0,23	0,6314

NDVI at tillering → the model could not be estimated.

Tiller dry weight

Model fitting values

N	AIC	BIC	logLik	Sigma	R2 0	R2 1	R2 2	R2 3	R2 4	R2 5
34	326,33	360,05	-140,17	6,9E-06	2,0E-03	2,0E-03	2,0E-03	2,0E-03	0,02	0,97

Marginal hypotheses tests (SC type III)

	numDF	denDF	F-value	p-value
(Intercept)	1	18	92,01	<0,0001
Treatment	1	18	1,466	<0,0001

Adjusted average and standard errors for *Treatment*
LSD Fisher (Alpha=0,05)
p-values adjustment procedure: No

Treatment	Average	S.E.	
Rizofos	101,86	10,37	**A**
Control	100,26	10,37	**B**

Average values followed by the same letter are not statistically different (p > 0,05)

Tiller fresh weight

Model fitting values

N	AIC	BIC	logLik	Sigma	R2 0	R2 1	R2 2	R2 3	R2 4	R2 5
102	1209,71	1269,63	-581,86	7,5E-04	0,02	0,02	0,02	0,02	0,02	0,88

Marginal hypotheses tests (SC type III)

	numDF	denDF	F-value	p-value
(Intercept)	1	86	117,11	<0,0001
Treatment	1	86	12,32	0,0007

Adjusted average and standard errors for *Treatment*
LSD Fisher (Alpha=0,05)
p-values adjustment procedure: No

Treatment	Average	S.E.	
Rizofos	489,90	43,86	**A**
Control	452,63	43,88	**B**

Average values followed by the same letter are not statistically different (p > 0,05)

Spike fresh weight

Model fitting values

N	AIC	BIC	logLik	Sigma	R2 0	R2 1	R2 2	R2 3	R2 4	R2 5
48	652,10	683,18	-309,05	1503655717,83	2,8E-03	2,8E-03	0,13	0,22	0,22	0,35

Marginal hypotheses tests (SC type III)

	numDF	denDF	F-value	p-value
(Intercept)	1	40	428,99	<0,0001
Treatment	1	40	11,04	0,0019

Adjusted average and standard errors for *Treatment*
LSD Fisher (Alpha=0,05)
p-values adjustment procedure: No

Treatment	Average	S.E.	
Control	1814,96	87,04	**A**
Rizofos	1719,33	86,01	**B**

Average values followed by the same letter are not statistically different (p > 0,05)

Spike dry weight → the model could not be estimated.

To summarize, the factors that showed the best performance (significative) for seed treatment with Rizofos® in comparison to non-bacterized seeds (control), were:

- **Grain yield.**
- **Number of spikes per m^2.**
- **Tiller fresh weight and tiller dry weight.**

Note: spike fresh weight was the only factor that showed an inverse effect when compared to the other factors, *i.e.,* spike fresh weight resulted higher for non-bacterized seeds than for seeds treated with Rizofos®.

Complementary analyses

If we consider the effect of **seed pre-treatment with fungicide** separately, so that samples are only influenced by the fungicide treatment (with or without bacterization), in most cases there was no significant impact of the fungicide (Compinche®) on grain yield (with the sole exception of samples from the campaign 2014/2015 in Ferré and Pergamino).

Ferre 2011/2012:

ANOVA (SC tipo III)

F.V.	SC	gl	CM	F	p-valor
Model	1674416,67	3	558138,89	4,62	0,0190
Treatment	847582,31	1	847582,31	7,02	0,0191
Seed treatment	333973,03	1	333973,03	2,77	0,1185
Treatment*Seed treatment	126031,86	1	126031,86	1,04	0,3243
Error	1690471,70	14	120747,98		
Total	3364888,37	17			

Pergamino 2021/2013:

ANOVA (SC tipo III)

F.V.	SC	gl	CM	F	p-valor
Model	487162,71	3	162387,57	4,76	0,0345
Treatment	400475,12	1	400475,12	11,74	0,0090
Seed treatment	82119,95	1	82119,95	2,41	0,1594
Treatment*Seed treatment	4567,64	1	4567,64	0,13	0,7239
Error	272885,73	8	34110,72		
Total	760048,44	11			

Junín 2014/2015:

ANOVA (SC tipo III)

F.V.	SC	gl	CM	F	p-valor
Model	648648,09	3	216216,03	0,33	0,8060
Treatment	474684,02	1	474684,02	0,72	0,4112
Seed treatment	26242,76	1	26242,76	0,04	0,8450
Treatment*Seed treatment	17611,02	1	17611,02	0,03	0,8727
Error	9259982,02	14	661427,29		
Total	9908630,11	17			

Ferre 2014/2015 (Klein Yarará):

ANOVA (SC tipo III)

F.V.	SC	gl	CM	F	p-valor
Model	689166,81	3	229722,27	40,39	<0,0001
Treatment	155101,58	1	155101,58	27,27	0,0008
Seed treatment corrected	515388,89	1	515388,89	90,62	<0,0001
Treatment*Seed treatment c..	18676,34	1	18676,34	3,28	0,1075
Error	45497,16	8	5687,15		
Total	734663,97	11			

Test:LSD Fisher Alpha=0,05 DMS=100,40296

Error: 5687,1456 gl: 8

Seed treatment corrected	Avg	n	S.E.	
None	3828,49	6	30,79	A
Compinche	4242,97	6	30,79	B

Average values followed by the same letter are not statistically different (p > 0,05)

Pergamino 2014/2015:

ANOVA (SC tipo III)

F.V.	SC	gl	CM	F	p-valor
Model	242296,74	3	80765,58	8,57	0,0070
Treatment	70380,24	1	70380,24	7,47	0,0257
Seed treatment	144103,62	1	144103,62	15,30	0,0045
Treatment*Seed treatment	27812,89	1	27812,89	2,95	0,1241
Error	75359,89	8	9419,99		
Total	317656,63	11			

Test:LSD Fisher Alpha=0,05 DMS=129,21844

Error: 9419,9864 gl: 8

Seed treatment corrected	Avg	n	S.E.	
Compinche	6588,35	6	39,62	A
None	6807,51	6	39,62	B

Average values followed by the same letter are not statistically different (p > 0,05)

Ferre 2015/2016:

ANOVA (SC tipo III)

F.V.	SC	gl	CM	F	p-valor
Model	1491675,86	3	497225,29	4,59	0,0104
Treatment	882646,55	1	882646,55	8,15	0,0083
Seed treatment corrected	226686,23	1	226686,23	2,09	0,1599
Treatment*Seed treatment	4764,38	1	4764,38	0,04	0,8355
Error	2815266,75	26	108279,49		
Total	4306942,62	29			

We did not detect any interaction between seed pre-treatment with the fungicide and seed bacterization, for all measured parameters (grain yield, number of plants, spikes or tillers) → the impact of seed bacterization with Rizofos® would not be affected by seed pre-treatment with fungicide.

The influence of **seed variety** could only be analyzed in one particular case: Ferré 2014/2015, in which field trials involved two varieties.

ANOVA (SC tipo III)

F.V.	SC	gl	CM	F	p-valor
Modelo	3115466,85	3	1038488,95	114,55	<0,0001
Treatment	218323,78	1	218323,78	24,08	0,0012
Variety	2853008,06	1	2853008,06	314,70	<0,0001
Treatment*Variety	44135,01	1	44135,01	4,87	0,0584
Error	72527,18	8	9065,90		
Total	3187994,03	11			

Test:LSD Fisher Alpha=0,05 DMS=126,76657
Error: 9065,8973 gl: 8

Variety	Avg	n	S.E.	
Klein Yarará	4242,97	6	38,87	A
Baguette 601	5218,17	6	38,87	B

Average values followed by the same letter are not statistically different (p > 0,05)

Even though there was a strong difference in the grain yield between different varieties, we did not detect any interaction between seed variety and seed bacterization.

The influence of the field trial **location** could not be evaluated without modifying the rest of the parameters (campaign/seed variety/fungicide treatment). If we analyze the whole data set, there was a clear effect of the location, with Pergamino having the highest grain yields. However, in this case, we neither detected an interaction between location and seed bacterization.

ANOVA (SC tipo III)

F.V.	SC	gl	CM	F	p-valor
Modelo	32322619,31	5	6464523,86	7,43	<0,0001
Treatment	3717993,87	1	3717993,87	4,27	0,0406
Location	26563940,79	2	13281970,40	15,26	<0,0001
Treatment*Location	13945,72	2	6972,86	0,01	0,9920
Error	125371735,00	144	870637,05		
Total	157694354,31	149			

Test:Tukey Alpha=0,05 DMS=531,35913
Error: 870637,0486 gl: 144

Location	Avg	n	S.E.	
Ferre	3928,18	84	101,81	A
Junin	4305,77	18	219,93	A
Pergamino	4860,32	48	134,68	B

Average values followed by the same letter are not statistically different (p > 0,05)

Supplementary Data 2

Soil Toxicity Test with the Nematode *Caenorhabditis elegans* RIZOFOS LIQ

Guideline ASTM E2172-01

Study Number: BI – 128744

MU 90451/D1

Ref. 11857

Date:

Sponsor:

Rizobacter S.A.

Avda. Dr. Arturo Frondizi N° 1150, Parque Industrial

CP B2702HDA – Pergamino, Buenos Aires – Argentina

Copy in English of the Original Report in Spanish

Study conducted by

MICROQUIM GROUP
Environmental Department
Microquim S.A.

Index

		Pages
Title		1
Index		2
A.	Technical Direction Statement	3
B.	Statement of Compliance with Good Laboratory Practice	4
C.	Preface	5
D.	Summary	8
E.	Purpose	9
F.	Test System and Materials	9
G.	Test Performance	10
H.	Results	12
I.	Conclusion	12
J.	Reference	12

Tables

1.	Soil Moisture and pH Determination	13
2.	Recovery of organisms-Mortality	14

Annex

I.	Certificate of Analysis provided by the Sponsor	15
II.	Quality Assurance Unit Statement	16
III.	GLP Certificate	17

Copy in English of the Original Report in Spanish

A) TECHNICAL DIRECTION STATEMENT

The study described in this final report was performed according to the Good Laboratory Practice (GLP) of the OECD (Organization for Economic Co-Operation and Development) review 1997 and published in 1998.

The documents under which this study was performed are those mentioned under the item “Test Guide”, and inside the Study Director Statement regarding the GLP Compliance besides the Study Plan Sponsor approval.

Technical Direction comments:………………………………………………………………

……………………………………………………………………………………………….

……………………………………………………………………………………………….

……………………………………………………………………………………………….

DATE: …. /…. /….

Copy in English of the Original Report in Spanish

B) STATEMENT OF COMPLIANCE WITH GOOD LABORATORY PRACTICE

TITLE: Soil Toxicity Test with the Nematode *Caenorhabditis elegans*. RIZOFOS LIQ.

TEST SUBSTANCE: RIZOFOS LIQ.

STUDY NUMBER: BI – 128744.

This study was conducted according to the OECD series on principles of Good Laboratory Practice and compliance monitoring, N°1, ENV/MC/CHEM (98) 17 OECD and pursuant to the written study plan, approved by the Study Director following the Standard Operating Procedures (SOP) stated in the Procedures of MICROQUIM GROUP This report is a true and accurate record of the results obtained, and there were no known circumstances that could have affected the quality and integrity of the data.

The results obtained, as well as any storage medium for electronically recorded data, all documentation, study plan and final report are retained in the corresponding archives at MICROQUIM GROUP.

This declaration does not apply to the information provided by the Sponsor (Certificate of Analysis of the sample, Certificate of the Reference Substance and any other information regarding the Identification and Characteristics of the Test Substance).

Copy in English of the Original Report in Spanish

Florencia Parodi
Biology Degree
Study Director **Date:**

C) PREFACE

C.1) GENERAL

Title: Soil Toxicity Test with the Nematode *Caenorhabditis elegans*. RIZOFOS LIQ.

Sponsor: **Rizobacter S.A.** Avda. Dr. Arturo Frondizi N° 1150, Parque Industrial CP B2702HDA – Pergamino, Buenos Aires – Argentina.

Test number: BI – 128744.

Test substance: RIZOFOS LIQ.

Test system: nematode *Caenorhabditis elegans.*

Testing Institution: MICROQUIM GROUP, Av. Triunvirato 3447, (1427) Ciudad Autónoma de Buenos Aires, Argentina

Address of the Study Director: Av. Triunvirato 3447, (1427) Ciudad Autónoma de Buenos Aires, Argentina.

C.2) STAFF

Study Director:	Florencia Parodi<br>Biology Degree
Test Performance:	Florencia Parodi<br>Biology Degree
Archive Responsible:	Silvina López

C.3) SCHEDULE

Date of entrance of the sample: January 06th 2021

Date of the beginning of the experimental phase of the test: January 21st 2021

Observation period: 24 hours.

Date of the end of the experimental phase: February 02nd 2021

C.4) TEST GUIDELINE

This test was carried out in compliance with the following method: Guideline ASTM E2172-01 "Standard Guide for Conducting Laboratory Soil Toxicity Tests with the Nematode *Caenorhabditis elegans*".

Copy in English of the Original Report in Spanish

C.5) GOOD LABORATORY PRACTICE

The Study Plan, the experimental phase, the final report and related standard operating procedures, were inspected periodically and the dates of inspections were included in the final report.

C.6) CERTIFICATIONS, REGISTRATION, ACCREDITATIONS AND REGISTERS

- Certification in compliance with the Principles of Good Laboratory Practice of the OECD (1998), issued by the **OAA**.
- **COFILAB** (Consejo de Fiscalización de Laboratorios).
- **SENASA** (Servicio Nacional de Sanidad y Calidad Agroalimentaria) LR 0060 registered as laboratory of agrochemicals analysis to perform physicochemical, toxicological studies and determination of pesticide residues in vegetable matrices.
- Animal facility registered at **SENASA** (Servicio Nacional de Sanidad y Calidad Agroalimentaria) according to regulations of Res. 617/02 for the production of toxicological and ecotoxicological data.
- **INSTITUTO COLOMBIANO AGROPECUARIO (ICA)**, part of the Treaty of the Andean Pact, Resolution No. 03431 as a Quality Control Laboratory of chemical pesticides for agricultural use.
- **EPA** (Environmental Protection Agency) assigned laboratory code number 955079.
- **Environment Aptitude Certificate** issued by the Government of the Buenos Aires City Res. 077-A.A. Law No. 123/2000.
- Laboratory of Industrial Analysis registered at **OPDS** (Organismo Provincial para el Desarrollo Sostenible) according to the provisions of Resolution No. 504/01. Registration No. 31.
- **SEDRONAR** (Secretaría de Programación para la Prevención de la Drogadicción y la Lucha contra el Narcotráfico) N° RN 858 PQ.
- **SENAVE** (Servicio Nacional de Calidad y Sanidad Vegetal y de Semillas) N° LR 0008.

Copy in English of the Original Report in Spanish

C.7) AMENDMENT PROCEDURE

There were no amendments to the Study Plan.

C.8) DEVIATION PROCEDURE

There were no deviations to the Study Plan.

C.9) ARCHIVES

The laboratory will preserve the following data at least for 6 years: study plan, digital copy of the report and original data, in the general archive situated at 3447 Triunvirato Av. (1427), Ciudad Autónoma de Buenos Aires, Argentina. During that period no data will be discarded without the Sponsor's consent.

If the Sponsor requests the documentations corresponding to the study (Study Plan, raw data, logbook and digital copies of the Final Report) it will remain in custody of the Sponsor. Otherwise, it will remain in the general archive situated at 3447 Triunvirato Av. (1427), Ciudad Autónoma de Buenos Aires, Argentina. This period of 6 years can be modified by specific request of the Sponsor. Once finished this period, the testing institution will deliver the documentation to the Sponsor, who will be responsible for this archive.

C.10) COMMITMENT OF CONFIDENTIALITY

The signatories of this final report are committed to safeguarding the confidentiality of all information involved in this study, both delivered by the Sponsor as that generated by this laboratory.

C.11) SAFETY PRECAUTIONS

This study was carried out taking precautions according to the type of the test substance.

Copy in English of the Original Report in Spanish

D) SUMMARY

Title: Soil Toxicity Test with the Nematode *Caenorhabditis elegans*. RIZOFOS LIQ.

Test substance: RIZOFOS LIQ.

The test was carried out on a previously treated artificial soil. Two Treatments and a negative control group were evaluated:

Negative Control (TT0): soil without germinating.

Treatment 1 (TT1): soil from the germination of seeds treated with the test substance.

Treatment 2 (TT2): soil from the germination of seeds treated with water.

Observation period: 24 hours

The acceptability criteria of the trial were met:

Recovery ≥ 80%, Control survival ≥ 90%.

Conclusion:

Under the conditions of this test, the recovery was greater than 80%. The mortality percentage for the three (3) evaluated treatments was less than 10%, therefore, no effect related to the test substance **RIZOFOS LIQ** was observed on the toxicity of the nematode *Caenorhabditis elegans* in soil.

Copy in English of the Original Report in Spanish

E) PURPOSE

The aim of this test was to assess the effect of **RIZOFOS LIQ** on the soil toxicity with the nematode *Caenorhabditis elegans*.

F) TEST SYSTEM AND MATERIALS

F.1) TEST ANIMALS

Test system: *Caenorhabditis elegans* (Nematode) N2 wild-type. Origin: Caenorhabditis Genetics Center (CGC) Universidad de Minessota EEUU.

Source: Neoambiental. Calle 15 N° 345, City Bell, Bs. As. Argentina.

Number and age of the test animals at the start of the test: Ten (10) 3-4 days-old worms from age-synchronized cultures, per replicate.

Distribution: at random in Petri dishes 35 x 10 mm.

Identification: number on replicate. Number of replicates:6.

Soil: A modified artificial soil is used with an organic matter content of 10%: 10% sphagnum peat, 20% kaolin clay, 70% fine industrial sand. Calcium carbonate is added to obtain pH of 7.0 +0.5 (add up to 0,4%)

Seeds treatment and germination in artificial soil, prior to toxicity testing:

Treatment 1: 200 g of wheat seeds were treated homogeneously with 1.6 ml of test substance.

Treatment 2: 200 g of wheat seeds were treated homogeneously with 1.6 ml of de-ionised water.

Treated seeds were germinated in artificial soil. 10 days later, plants were removed from soil and available soil was used for the toxicity assay.

Seeds treatment and germination were done according to Sponsor's protocol.

Equipment:

Analytical Balance (TR-014), Culture chamber (MA-043), pHmeter (MI-022), Stove (DQ-018), Centrifuge (MA-017).

Copy in English of the Original Report in Spanish

F.2) IDENTIFICATION AND CHARACTERISTICS OF THE TEST SUBSTANCE (According to information provided by the Sponsor)

Test Item Name:	RIZOFOS LIQ
Active ingredient:	*Pseudomonas fluorescens* cepa 1008
Bacterial concentration:	$\geq 1 \times 10^8$ UFC/mL (According to CoA of the Sponsor)
Batch Number:	Y0040076
Expiry Date:	15 March 2021
Product description:	Liquid
Storage conditions:	Room temperature

G) TEST PERFORMANCE

G.1) TEST CONDITIONS

Variables controlled during the test:

Test duration: 24h, No food was administered.

Temperature: remained in the range 20 ± 1°C.

Test was carried out under darkness.

Moisture content of the testing soil was in the range 35-45%.

pH range of 3.1 – 11.9.

G.2) TEST PROCEDURE

There were two Treatments and a negative control group:

Negative Control (TT0): soil not germinated

Treatment 1 (TT1): soil from germinated seeds treated with the test substance.

Treatment 2 (TT2): soil from germinated seeds treated with water.

Each soil batch was checked for soil humidity and water content adjusted to 40%. The quantity of soil in the batch was enough for the number of replicates, determinations of pH and soil moisture content.

2.33 g of soil (dry weight) was added to each container. (35 x 10 mm petri dishes).

Copy in English of the Original Report in Spanish

Test vessels were positioned randomly in the test incubator for a 3-day equilibration period approximately.

Soil humidity was checked at the beginning and at the end of the test.

pH was measured at the beginning and at the end of the test. Measurements were made in one extra control sample and one extra sample of the treated soil samples prepared and maintained in the same way as the test cultures, but without the addition of worms.

Once the equilibration period was finished, worms were transferred individually to each test vessel and placed onto the surface of the soil. Test containers were once again positioned randomly in the test incubator for the next 24 hours.

At the end of the test, mortality was assessed. Worms were extracted by centrifugation and flotation and counted.

<u>Protocols</u>:

<u>pH Measurement</u>: 23.33 g of soil and 15 ml of KCl 1M solution were equilibrated for 7 days. The pH was measured in the supernatant.

<u>Water content of the soil</u>: measured by the gravimetric method.

A subsample of fresh hydrated soil was weighed (Wm) in a container (T), oven dried at 105°C until there was no further mass loss (approx. 24 hours) and then reweighed (Ws) in the container (T). Using the following formula:

Gravimetric Water content (w) = (Wm - Ws)/Ws

<u>Worms Recovery</u>: After exposure, the soil and worms were rinsed from dishes into 50 ml centrifuge tubes with 20 ml of Ludox AS. Then centrifuged at 700g for 2 minutes to obtain a compact pellet. Tubes were set aside for 15minutes to allow time for the worms to buoy to the top of the solution. The solution was then poured into 100 mm petri dishes and viewed under loupe. Worms were removed and placed on K-agar plates with food source. If worms did not respond, they were scored as dead.

Copy in English of the Original Report in Spanish

H) RESULTADOS

Test Validity:

The acceptability criteria of the trial were met:

Recovery ≥ 80%, Control survival ≥ 90%.

I) CONCLUSION

Under the conditions of this test, the recovery was greater than 80%. The mortality percentage for the three (3) evaluated treatments was less than 10%, therefore, no effect related to the test substance **RIZOFOS LIQ** was observed on the toxicity of the nematode *Caenorhabditis elegans* in soil.

J) REFERENCES

Good Laboratory Practice – ENV/MC/CHEM (98) 17 OECD.

Guideline ASTM E2172-01 "Standard Guide for Conducting Laboratory Soil Toxicity Tests with the Nematode *Caenorhabditis elegans*".

The data contained in this report as well as the conclusion are the accurate reproduction of the raw data registered on the logbook: ODT 33366-128744-CLI31-MU90451/D1.

Copy in English of the Original Report in Spanish

MICROQUIM GROUP
FLORENCIA PARODI
Biology Degree
Study Director

TABLE N°1:

SOIL HUMIDITY AND PH DETERMINATION.

Treatment Group	% Initial Soil Humidity	% Final Soil Humidity	Initial pH	Final pH
Negative Control (TT0)	39	38	6.2	6.2
Treatment 1 (TT1)	38	38	6.1	6.1
Treatment 2 (TT2)	39	38	6.2	6.2

Copy in English of the Original Report in Spanish

TABLE N°2:

RECOVERY OF ORGANISMS- MORTALITY.

Number of nematodes per replicate at the start of the test: 10

Treatment Groups	Replicate	Recovery		Mortality	
		Recovery	% Recovery	Mortality	% Mortality
Negative Control (TT0)	T0.1	9/10	83,3	2/9	9,7
	T0.2	8/10		1/8	
	T0.3	8/10		1/8	
	T0.4	8/10		0/8	
	T0.5	9/10		1/9	
	T0.6	8/10		0/8	
Treatment 1 (TT1)	T1.1	9/10	83,3	1/9	10,0
	T1.2	8/10		2/8	
	T1.3	8/10		0/8	
	T1.4	9/10		1/9	
	T1.5	8/10		0/8	
	T1.6	8/10		1/8	
Treatment 2 (TT2)	T2.1	8/10	81,7	1/8	8,1
	T2.2	8/10		1/8	
	T2.3	9/10		1/9	
	T2.4	8/10		1/8	
	T2.5	8/10		0/8	
	T2.6	8/10		0/8	

Copy in English of the Original Report in Spanish

ANNEX I

Certificate of Analysis provided by the Sponsor

RIZOBACTER

CERTIFICADO DE ANÁLISIS

03.12.2020

**Producto**	Biofertilizante para uso sobre semillas, foliar y/o en surco. Industria Argentina.
**Cantidad**	2 Vejigas por 0.4 Litros

Lote	Cantidad	Fecha vencimiento
Y0040076	2	15.03.2021

Especificaciones	
Componente básico	*Pseudomonas fluorescens*
Concentración a la elaboración (1)	$\geq 1x10^9$ UFC/mL
Concentración al vencimiento (1)	$\geq 1x10^8$ UFC/mL
Pureza microbiológica (2)	Cultivo puro, libre de microorganismos contaminantes
pH	5.8 – 7.8

(1) Recuento de bacterias viables por siembra en superficie en agar F para Pseudomonas. Placas incubadas a 28°C ± 2°C.

(2) Detección de contaminantes por conteo de microorganismos heterotróficos en placas con Agar Tríptico de Soja (TSA) para bacterias y Agar Papa Glucosado para hongos.

Marisa Díaz
Microbióloga
Líder de I+D PGPM

ANNEX II

GLP Quality assurance unit statement

TITLE: Soil Toxicity Test with the Nematode *Caenorhabditis elegans*. RIZOFOS LIQ.

TEST SUBSTANCE: RIZOFOS LIQ.

STUDY NUMBER: BI – 128744.

This study was audited during its different stages. For this study, the final report was compared with the study plan and Standard Operating Procedures (SOP).

The report is in accordance with the all obtained data.

This report is a true and accurate record of the result obtained, and there were no known circumstance that could have affected the quality and integrity of the data.

The audit was carried out according to the Standard Operating Procedures (SOP) established in the Procedures Manual of MICROQUIM GROUP.

The audit report was remitted to Direction and the Study Director, filing a copy of it in the Safety Enclosure belonging to Digital Archive and Documents Department of MICROQUIM GROUP.

Study Phase	***Date(s) of Inspection***	***Date(s) of Reporting***
Study Plan Auditing		
Facilities Auditing		
Process based Inspections (Support Assembly and Observations Day 0)		
Process based Inspections (Count)		
Final Report Auditing		

GLP Quality Assurance Unit

Copy in English of the Original Report in Spanish

ANNEX III
GLP Certificate

Página 1 de 1

OAA
Organismo Argentino de Acreditación

Reconocido internacionalmente en los ámbitos de ILAC, IAF e IAAC

Bartolomé Mitre 648, 1° piso cfte.
(C1036AAL) Bs. As. Argentina
Teléfonos: 54-11 2150-2155 / 2156
info@oaa.org.ar / www.oaa.org.ar

OAA- Organismo Argentino de Acreditación
F13-(PRO-BPL) v2, F.e.V.= 23-marzo-2016

CERTIFICATE OF COMPLIANCE WITH OECD PRINCIPLES OF GOOD LABORATORY PRACTICE

Granted to the Testing Facility

GRUPO MICROQUIM- MICROQUIM S.A.

Av. Triunvirato 3447- CABA, Argentina

The Argentine Accreditation Body -OAA- declares that according to the requirements of its Good Laboratory Practice Monitoring Program, the mentioned Test Facility carries out the non-clinical studies mentioned in this certificate in compliance with the Principles of Good Laboratory Practice of the Organisation for Economic Co-operation and Development - OECD - (1998).

REGISTRATION OF COMPLIANCE WITH GLP No 01

AUTHORIZED REPRESENTATIVE: Alejandro Lucini

Area of expertise:

1-Physical-chemical testing
3-Mutagenicity testing
4- Ecotoxicity studies on aquatic and terrestrial organisms
5- Studies on behavior in water, soil and air; bioaccumulation
6- Residue studies (analytical phase).
8- Analytical and clinical Chemistry testing

Non-clinical study performed in: AGROCHEMICAL

The test facility has received the following inspections according to the Monitoring Program GLP - OECD established by OAA

Date	Type of Inspection
August 12, 13 and 14, 2009	Initial Inspection and study audit
10/2010, 09/2011, 11/2012, 05/2014, 11/2015	Re-inspection and study audit
March 13, 2015	Special Inspection and study audit
March 5 and 6, 2018	Re-Inspection and study audit
March 21 and 22, 2019	Special Inspection and study audit

President
Gonzalo Gómez Romero

This certificate is issued in Buenos Aires on May 28, 2019.

Supplementary Data 3

Chronic Toxicity Test on *Folsomia candida*

(Effects on Collembolans reproduction in soil)

RIZOFOS LIQ

Guideline OECD Nº 232

Study Number: BI - 125407

MU 90451

Ref. 11857

Date:

JULY 10 2020

Sponsor:

Rizobacter S.A.

Avda. Dr. Arturo Frondizi N° 1150, Parque Industrial

CP B2702HDA – Pergamino, Buenos Aires – Argentina

Copy in English of the Original Report in Spanish

Study conducted by

MICROQUIM GROUP

Department of Environmental Studies

Microquim S.A.

Index

		Pages
Title		1
Index		2
A.	Technical Direction Statement	3
B.	Statement of Compliance with Good Laboratory Practice	4
C.	Preface	5
D.	Summary	9
E.	Purpose	10
F.	Test System and Materials	10
G.	Test Performance	11
H.	Results	13
I.	Conclusion	14
J.	Reference	14

Tables

1.	Soil Moisture: Weight loss	15
2.	Soil pH	16
3.	Extraction: Mortality. Reproduction	17

Annex

I.	Statistical Analysis	19
II.	Certificate of Analysis provided by the Sponsor	22
III.	Quality Assurance Unit Statement	23
IV.	GLP Certificate	24

Copy in English of the Original Report in Spanish

A) TECHNICAL DIRECTION STATEMENT

The study described in this final report was performed according to the Good Laboratory Practice (GLP) of the OECD (Organization for Economic Co-Operation and Development) review 1997 and published in 1998.

The documents under which this study was performed are those mentioned under the item “Test Guide”, and inside the Study Director Statement regarding the GLP Compliance besides the Study Plan Sponsor approval.

Technical Direction comments:……………………………………………………….

…………………………………………………………………………………………….

…………………………………………………………………………………………….

…………………………………………………………………………………………….

DATE: …. /…. /….

Copy in English of the Original Report in Spanish

B) STATEMENT OF COMPLIANCE WITH GOOD LABORATORY PRACTICE

TITLE: Chronic Toxicity Test on *Folsomia candida.* (Effects on Collembolans reproduction in soil). RIZOFOS LIQ.

TEST SUBSTANCE: RIZOFOS LIQ.

STUDY NUMBER: BI – 125407.

This study was conducted according to the OECD series on principles of Good Laboratory Practice and compliance monitoring, N°1, ENV/MC/CHEM (98) 17 OECD and pursuant to the written study plan, approved by the Study Director following the Standard Operating Procedures (SOP) stated in the Procedures of MICROQUIM GROUP This report is a true and accurate record of the results obtained, and there were no known circumstances that could have affected the quality and integrity of the data.

The results obtained, as well as any storage medium for electronically recorded data, all documentation, study plan and final report are retained in the corresponding archives at MICROQUIM GROUP.

This declaration does not apply to the information provided by the Sponsor (Certificate of Analysis of the sample, Certificate of the Reference Substance and any other information regarding the Identification and Characteristics of the Test Substance).

Copy in English of the Original Report in Spanish

Florencia Parodi
Biology Degree
Study Director **Date:**

C) PREFACE

C.1) GENERAL

Title: Chronic Toxicity Test on *Folsomia candida*. (Effects on Collembolans reproduction in soil). RIZOFOS LIQ.

Sponsor: **Rizobacter S.A.** Avda. Dr. Arturo Frondizi N° 1150, Parque Industrial CP B2702HDA – Pergamino, Buenos Aires – Argentina.

Test number: BI – 125407.

Test substance: RIZOFOS LIQ.

Test system: Collembola *(Folsomia candida).*

Testing Institution: MICROQUIM GROUP, Av. Triunvirato 3447, (1427) Ciudad Autónoma de Buenos Aires, Argentina

Address of the Study Director: Av. Triunvirato 3447, (1427) Ciudad Autónoma de Buenos Aires, Argentina.

Copy in English of the Original Report in Spanish

C.2) STAFF

Study Director:	Florencia Parodi<br>Biology Degree
Test Performance:	Florencia Parodi<br>Biology Degree
Archive Responsible:	Silvina López

C.3) SCHEDULE

Date of entrance of the sample: January 30th 2020

Date of the beginning of the experimental phase of the test: May 14th 2020

Observation period: 28 days.

Date of the end of the experimental phase: June 19th 2020

C.4) TEST GUIDELINE

This test was carried out in compliance with the following method: Guideline Nº 232 “Collembolan Reproduction Test in soil” by Organization for Economic Cooperation and Development (OECD), adopted on 29 July 2016.

C.5) GOOD LABORATORY PRACTICE

The Study Plan, the experimental phase, the final report and related standard operating procedures, were inspected periodically and the dates of inspections were included in the final report.

C.6) CERTIFICATIONS, REGISTRATION, ACCREDITATIONS AND REGISTERS

- Certification in compliance with the Principles of Good Laboratory Practice of the OECD (1998), issued by the **OAA**.
- **COFILAB** (Consejo de Fiscalización de Laboratorios).
- **CALIBA** (Cámara Argentina de Laboratorios Independientes, Bromatológicos, Ambientales y Afines).
- **SENASA** (Servicio Nacional de Sanidad y Calidad Agroalimentaria) LR 0060 registered as laboratory of agrochemicals analysis to perform physicochemical, toxicological studies and determination of pesticide residues in vegetable matrices.
- Animal facility registered at **SENASA** (Servicio Nacional de Sanidad y Calidad Agroalimentaria) according to regulations of Res. 617/02 for the production of toxicological and ecotoxicological data.
- **ICA (**Instituto Colombiano Agropecuario**)**, part of the Treaty of the Andean Pact, Resolution No. 03431 as a Quality Control Laboratory of chemical pesticides for agricultural use.
- **EPA** (Environmental Protection Agency) assigned laboratory code number 955079.
- **Environment Aptitude Certificate** issued by the Government of the Buenos Aires City Res. 077-A.A. Law No. 123/2000.

Copy in English of the Original Report in Spanish

- Laboratory of Industrial Analysis registered at **OPDS** (Organismo Provincial para el Desarrollo Sostenible) according to the provisions of Resolution No. 504/01. Registration No. 31.
- **SEDRONAR** (Secretaría de Programación para la Prevención de la Drogadicción y la Lucha contra el Narcotráfico) Nº RN 858 PQ.

C.7) AMENDMENT PROCEDURE

An amendment to Study Plan was made, it did not affect the quality or data integrity.

This final report can be amended by the Study Director. The Study Director will sign detailed descriptions of all amendments. The amendment will be effective at the time of Study Director's signature and informed to the Sponsor.

C.8) DEVIATION PROCEDURE

There were no deviations to the Study Plan.

C.9) ARCHIVES

The laboratory will preserve the following data at least for 6 years: study plan, digital copy of the report and original data, in the general archive situated at 3447 Triunvirato Av. (1427), Ciudad Autónoma de Buenos Aires, Argentina. During that period no data will be discarded without the Sponsor's consent.

If the Sponsor requests the documentations corresponding to the study (Study Plan, raw data, logbook and digital copies of the Final Report) it will remain in custody of the Sponsor. Otherwise, it will remain in the general archive situated at 3447 Triunvirato Av. (1427), Ciudad Autónoma de Buenos Aires, Argentina. This period of 6 years can be modified by specific request of the Sponsor. Once finished this period, the testing institution will deliver the documentation to the Sponsor, who will be responsible for this archive.

Copy in English of the Original Report in Spanish

C.10) COMMITMENT OF CONFIDENTIALITY

The signatories of this final report are committed to safeguarding the confidentiality of all information involved in this study, both delivered by the Sponsor as that generated by this laboratory.

C.11) SAFETY PRECAUTIONS

This study was carried out taking precautions according to the type of the test substance.

Copy in English of the Original Report in Spanish

D) SUMMARY

Title: Chronic Toxicity Test on *Folsomia candida.* (Effects on Collembolans reproduction in soil). RIZOFOS LIQ.

Test substance: RIZOFOS LIQ.

Dose levels: 100 µg/bee.

The test was carried out on a previously treated artificial soil. Two Treatments and a negative control group were evaluated:

Negative Control (TT0): soil without germinating.

Treatment 1 (TT1): soil from the germination of seeds treated with the test substance.

Treatment 2 (TT2): soil from the germination of seeds treated with water.

Observation period: 28 days

The validity criteria of the trial for the control group were met:

Maximum mean mortality 20%, Minimum reproduction rate of 100 juveniles and Maximum coefficient of variation 30%.

Statistical Analysis:

The Hypothesis tests did not show evidence of lack of Normality (Shapiro-Wilkis), nor was there significant evidence of lack of Homocedasticity, constant variance between groups (Levene's Test).

ANOVA: The hypothesis of equality of means between the treatments is tested. In order to assess statistical significance, the p-value is examined. Since the p-value is greater than the specified level of significance α, the difference between means is not statistically significant.

Conclusion:

The ANOVA statistical analysis showed that there are no significant differences (p> 0.05) between the different treatments.

Under the conditions of this trial, **RIZOFOS LIQ** showed no toxicity to *Folsomia candida.*

Copy in English of the Original Report in Spanish

E) PURPOSE

The purpose of the study was to determine the effect of **RIZOFOS LIQ** on the reproduction of Collembola in soil.

F) TEST SYSTEM AND MATERIALS

F.1) TEST ANIMALS

Test system: *Folsomia candida* (Collembolans). Synchronous juveniles were obtained according to Annex 3, OECD 232, page 15: "Guidance on rearing and synchronisation of *F.candida*."

Source: Neoambiental. Calle 15 N° 345, City Bell, Bs. As. Argentina.

Number and age of the test animals at the start of the test: Ten (10) juvenile females 9-12 days old, per replicate.

Distribution: at random in transparent glass containers with perforated caps to allow ventilation.

Identification: number on replicate. Number of replicates:8.

Soil: A modified artificial soil is used with an organic matter content of 5%: 5% sphagnum peat, 20% kaolin clay, approximately 74% fine industrial sand.

Seeds treatment and germination in artificial soil, prior to toxicity testing:

Treatment 1: 200 g of wheat seeds were treated homogeneously with 1.6 ml of test substance.

Treatment 2: 200 g of wheat seeds were treated homogeneously with 1.6 ml of de-ionised water.

Treated seeds were germinated in artificial soil. 10 days later, plants were removed from soil and available soil was used for the toxicity assay.

Seeds treatment and germination were done according to Sponsor's protocol.

Equipment:

Analytical Balance (TR-014), Culture chamber (MA-043), pHmeter (MI-022), Stove (DQ-018).

Copy in English of the Original Report in Spanish

F.2) IDENTIFICATION AND CHARACTERISTICS OF THE TEST SUBSTANCE (According to information provided by the Sponsor)

Test Item Name:	RIZOFOS LIQ
Active ingredient:	*Pseudomonas fluorescens* cepa 1008
Bacterial concentration:	4.65 x 10^9 UFC/mL (According to CoA of the Sponsor)
Batch Number:	MCY0010067
Expiry Date:	June 2020
Product description:	Liquid
Storage conditions:	Room temperature

Copy in English of the Original Report in Spanish

G) TEST PERFORMANCE

G.1) TEST CONDITIONS

Variables controlled during the test:

Temperature: remained in the range 20 ± 1°C.

Lighting: 400-800 lux, with photoperiods of 16 light hours - 8 hours dark.

Soil moisture: remained in the range 40-60% of the maximum WHC.

pH: was in the range 6.3 + 0.2

G.2) TEST PROCEDURE

Initially, the maximum WHC of the soil was determined according to the procedure described in Annex 5 of the OECD guide 232. Considering that the soil was watered at field capacity during the germination prior to the test; the gravimetric humidity of the 3 soil types, TT0, TT1 and TT2 was evaluated in order to adjust the humidity to 40-60% WHC. The soils were in the correct humidity range for the test.

Approximately 30g of soil from each of the treatments (8 replicates) were placed in each container. They were then weighed, recording the initial weight.

The Collembola were individually transferred to each container and placed on the ground surface.

Two Treatments and a negative control group were evaluated:

Negative Control (TT0): soil without germinating.

Treatment 1 (TT1): soil from the germination of seeds treated with the test substance.

Treatment 2 (TT2): soil from the germination of seeds treated with water.

Containers were randomly distributed within the incubation chamber and those positions were rotated randomly weekly.

Feeding: Approximately 5 mg of commercial dry yeast was added to each container at the start of the trial and after two weeks.

To determine the loss of soil moisture, the containers were weighed at the beginning, at 14 days and at the end of the test. After 14 days of testing, the lost water was replaced with deionized water, by gravimetric method.

The pH was determined at the beginning and at the end of the test according to the Procedure indicated in Annex 6 of the OECD 232 guide.

At the end of the test, day 28, the collembola were extracted by the flotation method:

The soil in each container is gradually flooded, 20 ml of water are added at a time to a final volume of 200ml. The collembolla float on the surface and are recovered with a Pasteur pipette to be transferred to a Petri dish for counting with the naked eye and with the help of a magnifying glass.

Adult mortality and number of juveniles were evaluated. Unrecovered adults were considered dead.

<u>G.3) REFERENCE SUBSTANCE</u>

The sensitivity of the test system was evaluated with technical grade Boric Acid (Brand: Analar, Lot: 17I154119, Expiration: August 24, 2020) as a reference substance. The dose 100mg/kg artificial soil was evaluated, which reduced reproduction more than 50%.

Copy in English of the Original Report in Spanish

H) RESULTADOS

Test Validity:

The validity criteria of the trial for the control group were met:

Maximum mean mortality 20%, Minimum reproduction rate of 100 juveniles and Maximum coefficient of variation 30%.

Statistical Analysis:

Excel XLSTAT 2020.3.1.16 statistical software was used.

The reproduction values obtained (number of juveniles per container) were statistically evaluated using ANOVA, in order to compare the different treatments (TT0, TT1 and TT2).

As a prerequisite, a hypothesis test is performed ($p \leq 0.05$):

Test of Normality (Shapiro-Wilkis)

Homegeneity of Variance (Homocedasticity) (Levene's Test)

Results:

The Hypothesis tests did not show evidence of lack of Normality. There is no significant evidence of lack of Homocedasticity, constant variance between groups.

ANOVA: The hypothesis of equality of means between the treatments is tested. In order to assess statistical significance, the p-value is examined. Since the p-value is greater than the specified level of significance α, the difference between means is not statistically significant.

Copy in English of the Original Report in Spanish

I) CONCLUSION

The ANOVA statistical analysis showed that there are no significant differences ($p > 0.05$) between the different treatments.

Under the conditions of this trial, **RIZOFOS LIQ** showed no toxicity to *Folsomia candida.*

J) REFERENCES

Good Laboratory Practice – ENV/MC/CHEM (98) 17 OECD.

Guideline Nº 232: "Collembolan Reproduction Test in soil" by Organization for Economic Cooperation and Development (OECD), adopted on 29 July 2016.

Excel XLSTAT 2020.3.1.16 statistical software

The data contained in this report as well as the conclusion are the accurate reproduction of the raw data registered on the logbook: ODT 31646-125725/M1-CLI454-MU90626.

Copy in English of the Original Report in Spanish

MICROQUIM GROUP
FLORENCIA PARODI
Biology Degree
Study Director

TABLE N°1:

SOIL HUMIDITY. WEIGHT LOSS.

Treatment Group	Replicate	Weight loss (%)	
		Day 0-14	Day 14-28
Negative Control (TT0)	T0.1	2,42	1,09
	T0.2	1,15	2,45
	T0.3	0,91	2,07
	T0.4	1,37	1,91
	T0.5	1,08	1,18
	T0.6	2,32	1,78
	T0.7	2,26	2,65
	T0.8	1,45	2,09
Treatment 1 (TT1)	T1.1	0,98	2,67
	T1.2	0,81	1,88
	T1.3	1,05	2,37
	T1.4	1,69	1,17
	T1.5	2,72	1,98
	T1.6	1,52	1,11
	T1.7	2,24	2,18
	T1.8	1,71	1,90
Treatment 2 (TT2)	T2.1	1,41	2,65
	T2.2	1,06	2,73
	T2.3	0,98	2,10
	T2.4	1,55	1,23
	T2.5	2,45	0,96
	T2.6	1,04	1,43

Copy in English of the Original Report in Spanish

	T2.7	0,88	2,30
	T2.8	2,19	1,70
Reference Substance	R1	1,65	1,93
	R2	1,14	1,71
	R3	0,72	1,92
	R4	1,50	1,63
	R5	1,96	0,70
	R6	2,55	1,32
	R7	1,71	2,24
	R8	1,45	1,09

TABLE N°2:

DETERMINATION OF THE SOIL PH

Treatment Group	pH day 0	pH day 28
Negative Control (TT0)	6,1	6,2
Treatment 1 (TT1)	6,2	6,3
Treatment 2 (TT2)	6,3	6,4
Reference Substance	6,2	6,4

Copy in English of the Original Report in Spanish

TABLE N°3:

EXTRACTION: MORTALITY. REPRODUCTION

Number of adults per replicate at the start of the test: 10

CV = % STD/Media

Treatment Groups	Replicate	Mortality		Reproduction		
		Mortality	% Mortality	Number of juveniles	Mean (STD)	CV
Negative Control (TT0)	T0.1	0/10	11,25	127	189,88 (40,08)	21,1
	T0.2	1/10		149		
	T0.3	1/10		184		
	T0.4	0/10		188		
	T0.5	2/10		176		
	T0.6	3/10		224		
	T0.7	2/10		241		
	T0.8	0/10		230		
Treatment 1 (TT1)	T1.1	1/10	12,5	143	185,88 (47,85)	25,7
	T1.2	2/10		128		
	T1.3	2/10		162		
	T1.4	1/10		209		
	T1.5	2/10		245		
	T1.6	1/10		156		
	T1.7	0/10		185		
	T1.8	1/10		259		
Treatment 2 (TT2)	T2.1	0/10	10	256	205,63 (38,93)	18,9
	T2.2	1/10		189		
	T2.3	3/10		175		
	T2.4	0/10		210		
	T2.5	2/10		137		
	T2.6	1/10		205		

Copy in English of the Original Report in Spanish

	T2.7	1/10		226		
	T2.8	0/10		247		
Reference Substance	R1	3/10	17,5	93	79,88 (16,69)	20,9
	R2	1/10		67		
	R3	0/10		50		
	R4	4/10		79		
	R5	2/10		103		
	R6	1/10		89		
	R7	2/10		86		
	R8	1/10		72		

SUMMARY OF RESULTS

	% Mortality	Reproduction Mean (STD)	CV
**Negative Control (TT0)**	11,25	189,88 (40,08)	21,1
**Treatment 1 (TT1)**	12,5	185,88 (47,85)	25,7
**Treatment 2 (TT2)**	10	205,63 (38,93)	18,9
**Reference Substance**	17,5	79,88 (16,69)	20,9

Copy in English of the Original Report in Spanish

ANNEX I

STATISTICAL ANALYSIS

Descriptive statistics (Quantitative data):

Statistic	TT0	TT1	TT2	Reference Substance
No. of observations	8	8	8	8
Minimum	127,00	128,00	137,00	50,00
Maximum	241,00	259,00	256,00	103,00
Mean	**189,88**	**185,88**	**205,63**	**79,88**
Variance (n-1)	1606,13	2289,84	1515,41	278,41
Standard deviation (n-1)	**40,08**	**47,85**	**38,93**	**16,69**
Coefficient of variation (n-1)	**0,211**	**0,257**	**0,189**	**0,209**
Standard error	14,17	16,92	13,76	5,90
Lower limit of the mean (95%)	156,37	145,87	173,08	65,93
Upper limit of the mean (95%)	223,38	225,88	238,17	93,82

Normality Test:

Shapiro-Wilk test (number of juveniles):

W	0,953
p-value	0,311
alpha	0,05

Interpretation of the test:
H0: The variable from which the sample was drawn follows a Normal distribution.
Ha: The variable from which the sample was drawn does not follow a Normal distribution.
Since the calculated p-value is greater than the significance level alpha = 0.05, the null hypothesis H0 cannot be rejected.

Copy in English of the Original Report in Spanish

Graphic P-P (normal distribution):

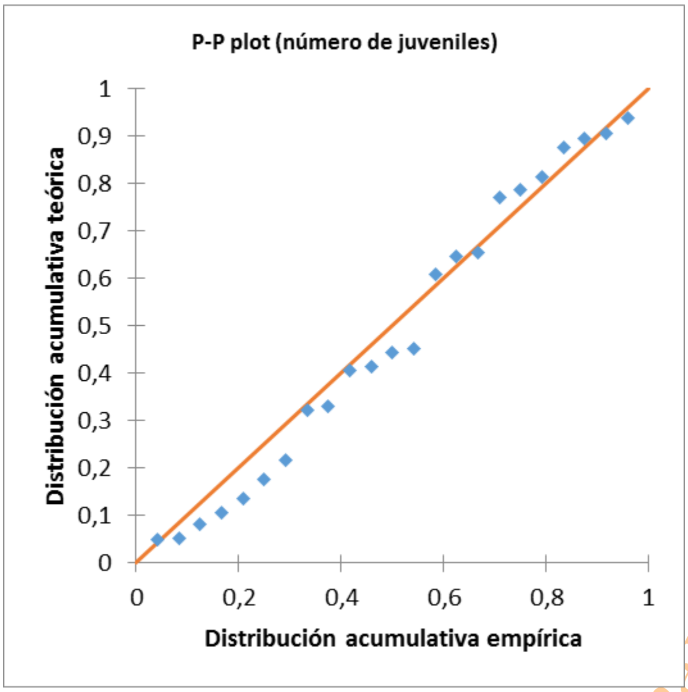

Copy in English of the Original Report in Spanish

<u>Variance Homogeneity Test:</u>

Levene's Test:

F (Observed value)	0,390
F (Critical value)	3,467
GL1	2
GL2	21
p-value	0,682
alpha	0,05

Interpretation of the test:
H0: The variances are identical.
Ha: At least one of the variances is different from the other
Since the calculated p-value is greater than the significance level alpha = 0.05, the null hypothesis H0 cannot be rejected.

ANOVA

Analysis of variance (number of juveniles):

Source	DF	Sum of Squares	Mean of Squares	F	Pr > F
Model	2	1744,333	872,167	0,484	0,623
Error	21	37879,625	1803,792		
Total	23	39623,958			

Calculated against the model Y = Average (Y)

Interpretation:
The analysis of variance indicates that there are no significant differences on the treatments analyzed, since the calculated p-value is greater than the level of significance alpha = 0.05.

Copy in English of the Original Report in Spanish

ANNEX II
Certificate of Analysis provided by the Sponsor

RIZOBACTER

CERTIFICADO DE ANALISIS

Producto	Inoculante para aplicaciones en semillas, foliares y/o surcos de siembra. Industria Argentina.
Marca comercial	**RIZOFOS LIQ**
Cantidad	2 sachets de 160 mililitros

Lote/Partida	***Cantidad de sachets***	***Vencimiento***
MCY0010067	2	6/2020

Componente básico	*Pseudomonas fluorescens* cepa 1008	
Especificaciones	**Rangos**	**Valores específicos para el lote MCY0010067**
Concentración bacteriana a la elaboración *(Ref.1)*	≥ 1 x 10^{9} UFC/mL	4,65 x 10^{9} UFC/mL
Concentración bacteriana al vencimiento	≥ 1 x 10^{8} UFC/mL	-
Pureza microbiológica *(Ref.2)*	Cultivo puro, libre de microorganismos contaminantes	Puro
pH	5,80 – 7,8	6,08
Densidad	1,04 – 1,1 g/mL	No determinado

Ref.1: Recuento de *Pseudomonas* viables mediante la técnica de siembra en placa por extensión en superficie en medio Agar F (AF).

Ref.2: Detección de contaminantes mediante recuento de microorganismos heterotróficos con la técnica de siembra en placa por extensión en superficie en medios Tripteína Soja Agar (TSA) para bacterias y Agar Papa Dextrosa (APD) para hongos.

Mic. Marisa Díaz
Líder de I+D PGPM

Avda. Dr. Arturo Frondizi N° 1150
Parque Industrial - C.P. B2702HDA
Pergamino (Bs. As.) - Argentina
TE: +54 2477 409400 - FAX: +54 2477 432893
info@rizobacter.com.ar
www.rizobacter.com

ANNEX III

GLP Quality assurance unit statement

TITLE: Chronic Toxicity Test on *Folsomia candida*. (Effects on Collembolans reproduction in soil). RIZOFOS LIQ.

TEST SUBSTANCE: RIZOFOS LIQ.

STUDY NUMBER: BI – 125407.

This study was audited during its different stages. For this study, the final report was compared with the study plan and Standard Operating Procedures (SOP).

The report is in accordance with the all obtained data.

This report is a true and accurate record of the result obtained, and there were no known circumstance that could have affected the quality and integrity of the data.

The audit was carried out according to the Standard Operating Procedures (SOP) established in the Procedures Manual of MICROQUIM GROUP.

The audit report was remitted to Direction and the Study Director, filing a copy of it in the Safety Enclosure belonging to Digital Archive and Documents Department of MICROQUIM GROUP.

Study Phase	***Date(s) of Inspection***	***Date(s) of Reporting***
Study Plan Auditing		
Facilities Auditing		
Process based Inspections		
Final Report Auditing		

GLP Quality Assurance Unit

Copy in English of the Original Report in Spanish

<u>ANNEX IV</u>
GLP Certificate

Página 1 de 1

OAA
Organismo Argentino de Acreditación

Reconocido internacionalmente en los ámbitos de ILAC, IAF e IAAC

Bartolomé Mitre 648, 1° piso cfte.
(C1036AAL) Bs. As. Argentina
Teléfonos: 54-11 2150-2155 / 2156
info@oaa.org.ar / www.oaa.org.ar

OAA- Organismo Argentino de Acreditación
F13-(PRO-BPL) v2, F.e.V.= 23-marzo-2016

CERTIFICATE OF COMPLIANCE WITH OECD PRINCIPLES OF GOOD LABORATORY PRACTICE

Granted to the Testing Facility

GRUPO MICROQUIM- MICROQUIM S.A.

Av. Triunvirato 3447- CABA, Argentina

The Argentine Accreditation Body -OAA- declares that according to the requirements of its Good Laboratory Practice Monitoring Program, the mentioned Test Facility carries out the non-clinical studies mentioned in this certificate in compliance with the Principles of Good Laboratory Practice of the Organisation for Economic Co-operation and Development - OECD - (1998).

REGISTRATION OF COMPLIANCE WITH GLP No 01

AUTHORIZED REPRESENTATIVE: Alejandro Lucini

Area of expertise:

1-Physical-chemical testing
3-Mutagenicity testing
4- Ecotoxicity studies on aquatic and terrestrial organisms
5- Studies on behavior in water, soil and air; bioaccumulation
6- Residue studies (analytical phase).
8- Analytical and clinical Chemistry testing

Non-clinical study performed in: AGROCHEMICAL

The test facility has received the following inspections according to the Monitoring Program GLP - OECD established by OAA

Date	Type of Inspection
August 12, 13 and 14, 2009	Initial Inspection and study audit
10/2010, 09/2011, 11/2012, 05/2014, 11/2015	Re-inspection and study audit
March 13, 2015	Special Inspection and study audit
March 5 and 6, 2018	Re-Inspection and study audit
March 21 and 22, 2019	Special Inspection and study audit

President
Gonzalo Gómez Romero

This certificate is issued in Buenos Aires on May 28, 2019.

Supplementary data 4

Faculty of Veterinary Science
National University of La Pampa
General Pico
La Pampa

SPECIAL MICROBIOLOGY CHAIR

EXPERIMENTAL DEVELOPMENT OF *Pseudomonas fluorescens* POSSIBLE PATHOGEN EFFECT IN MURIDAES ANIMAL MODEL.

P. fluorescens is a rod-shape or slightly curved bacillus of 0,5 to 1µm per 1,5 to 4,0 µm, Gram negative, motile because of the presence of a polar flagellum crest, aerobic, with respiratory metabolism, never fermentative, they use the H_2 or the CO_2 as electrons giver, they can use nitrate as electrons acceptor in anaerobiosis, they are chemoorganotrophs. They show very simple nutritive requirements; they are mesophilus and develop neutral pH. Oxidase positive, catalase positive, indole and methyl red negative, it does not produce piocianine and it does not grow at 43°C, it licuates gelatine, develops at 4 °C (these last tests are used to differentiate it from *P. aeruginosa)* (Brok, 2004).
P. fluorescens is considered pathogen in fish in which develops together with *Aeromonas hydrophila* the disease known as Haemorraghic Septicaemia. With regards to its importance to human health, it is uncommonly pathogen because it does not grow well at 37°C, as environmental contaminant may grow in refrigerated hematologic products (Brok, 2004). It is important to stress that this germ is considered the same as a typical opportunistic pathogen for mankind; it is found in hospital environment as contaminant of water sources, water solutions of quaternary ammonium, ponds and soils, among others (Bolaños and Díaz Rosa, 1997). It has been isolated from open cutaneous injuries, as well as from the urinary tract, although its virulence is soft and its pathogen power is discussed (Todar, 2004).

It was evaluated the possible pathogen effect of *P. fluorescens* suspension, that belongs to RIZOFOS LIQ biofertilizer, provided by RIZOBACTER ARGENTINA S.A

<u>PROTOCOL AND RESULTS OF THE ASSAY</u>:

PURITY AND CONCENTRATION EVALUATION OF THE MICROORGANISM IN THE COMMERCIAL INOCULANT

1.Purity of bacterial suspension was evaluated by inoculating agar base plates (B02-137-05) with 10 % of equine blood and Agar F *Pseudomonas* (B02-211-05) with 0,1 mL of homogenized suspension, it was incubated at 29 ºC for 24h. Biochemical tests were practiced to the developed colonies using APINE system.

<u>Result</u>: Inoculated plates showed uniform development of Gram negative bacillus, oxidase positive, with fluorescent pigment (Photo 1). Resulting API compatible with *P. fluorescens* (Photos 2 and 3)

Photo l. Agar F : fluorescent pigment

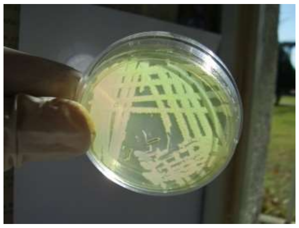

Photo 2. Inoculated biochemical tests with *P fluorescens* Gallery

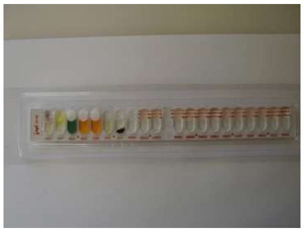

Photo 3: Main biochemical tests in *Pseudomonas* differentiation

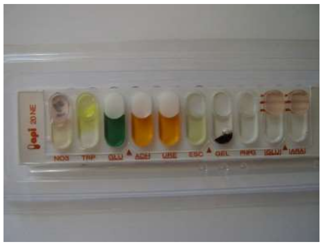

Nitrates: weak positive
Indole: negative
Glucose fermentation: negative
Arginine dehidrogenase in anaerobiosis: positve
Urea: positive
Esculine: negative
Gelatinase: positive

2. The count of viable ones was done by the dilution method in plate using the *Pseudomonas* Agar F (B02-2]) -05) mean culture. Two plates with 100 µL were planted, same were incubated at 29°C for 24 h for each dilution

<u>Result</u>: The analyzed suspension showed a count of viable ones of 6.4 x 10^9 bacteria/mL.

EVALUATION OF EXPERIMENTAL PATHOGEN POWER IN MURINO MODEL

A muridae animal model was used for the development of the possible pathogen power, using *Mus músculus* mice of CPZ:CrP:CFl genetical designation, *Outbried mouse* (albine), provided by biotery from Veterinary Sciences School of La Pampa National University.
Taking into account that *Pseudamonas fluorescens* is an opportunistic pathogen so it is responsible for infections in stressed individuals, an assay that considers animals in normal physiological state and animals submitted to stress was done.
The term stress involves a series of noxious stimuli that determine the increase of the circulating adenocorticotropic hormone (ACTH); most of these also activate the Sympathetic Nervous System decreasing the motility and the blood flow to mucous level, keeping a vascular reactivity to the catecholamines, and then causing a decrease of the mucous blood flow as an answer to the tension. The restriction to the blood flow at gastric mucous level causes its necrosis, penetrating through the muscular of the mucous to the inside of the sub mucous or deeper constituting the injury known as gastric ulcer (Toso, R.;2001).
On the basis of the enunciated concepts in previous paragraphs, animals in normal physiological state and animals stressed by hypothermia (bath at 22º C) and immobilization were used. As an inoculation way it was used the nasal-oral instillation (Photos 4,5 and 6) (Domínguez. 1997).

1) Non stressed check lot inoculated with *P fluorescens* nasal way, ocular way and orally kept at room temperature (10 animals)
2) Stressed by hypothermia and immobilization check lot, without inoculation (5 animals)

3) Stressed by hypothermia and immobilization check inoculated with *P fluorescens* nasal way, ocular way and orally (10 animals) (Photo 7).

Inoculation was done according to what it is detailed in table .

	Bacterial		Nasal	Bacterial		Orally	Bacterial	Inoculated
Ocular way	Dose		Way	Dose			Dose	Total
	R. Eye	L. Eye		R.N.	L.N.			
Zero	10^7	10^7	Zero	10^7	10^7	Zero	10^8	
Time			Time			zero		
1 Time	10^7	10^7	1 Time	10^7	10^7	1 Time	10^8	
Inoculated			Inoculated			Inoculated		
Total	4 x10^7		Total	4 x 10^7		Time	2 x 10^8	
								2,8 x 10^8

Table l. Ways and inoculated doses

Inoculation in non stressed and stressed lots (1 and 3) were done before animals were subject to stress and after it.

Photo 4. Nasal inoculation

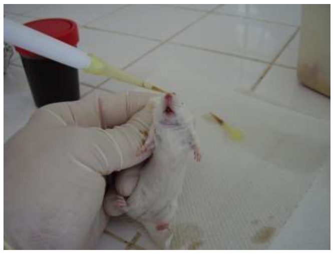

Photo 5: Oral Inoculation

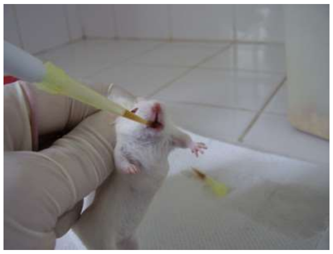

Photo 6: View of the eyeball inoculated

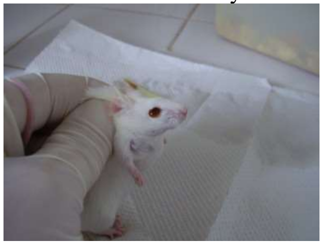

Photo 7: Animals under stress by hypothermia and immobilization

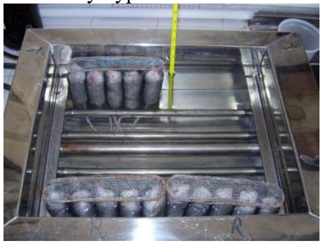

Animals from lots 1 and 3 were inoculated at zero time of the experiment and were inoculated again at 5 h from the beginning. (Table 1), they were distributed in conventional cages and kept in a partially isolated area, observing possible changes in the behavior, at 72h the 50% of the animals were bleeded by intracardiac puncture (Photo 8); O. 5 mL of blood were poured in the mean culture. Neonatal hemoculture (B07-205-86 Britania) incubating at 29°C in aerobiosis, making replications in blood agar base (B02-149-06) with 10% of equine blood defibrinated and agar Müller Hinton (B02-137-05 Britania)at 24, 48, 72 and 96 h. .

After blood extraction animals were sacrificed and necropsied, observing in detail internal organs and collecting samples from spleen, lung, liver, and eyeball in formol at 10% for histopathology studies (Photo 9).

At 120 hs. bleeding was done, then sacrifice and samples collection of the remaining 50%.

Photo 8. Blood extraction, by cardiac puncture

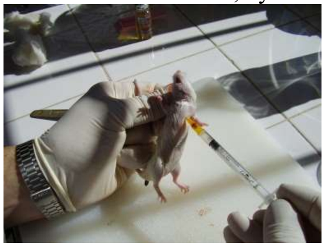

Photo 9. Samples of internal organs for histopatohology and hemoculture.

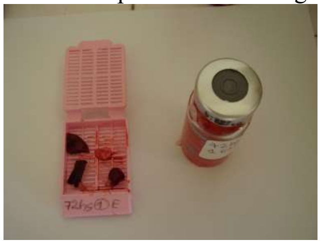

Result: Stress control lot (N°2), 100% of the animals showed ulcer in the gastric mucous, confirming this way that stress by hypothermia and immobilization was effective (Photo 10 and 11).

Photo 10. Stomach Post mortem Insuflation procedure, previous to ulcer exam

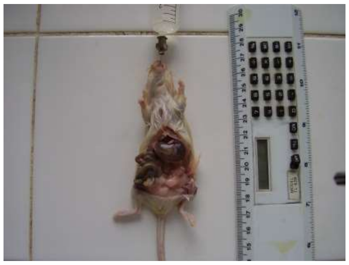

Photo 11. Visualization of ulcer in gastric mucous.

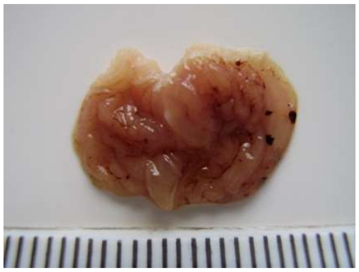

The lot of stressed and inoculated animals with *P. fluorescens* suspension showed a slight depression, lack of answer to external stimuli and indifference to food, during first 24 h., recovering it gradually. The lot of inoculated and non stressed animals didn´t show alteration in the behavior from the inoculation until the moment of the cardiac puncture.
The hemocultures and replications did not show bacterial development at 24, 48, 72 and 96h.

The histopathology did not show outstanding cellular changes compatible with acute bacterial infection. The absense of cellular changes in the liver compatible with bacterial and/or toxical hepatitis can be observed (Photo 12); the spleen did not show outstanding changes in the stroma (Fig. 13), the lungs did not show neither inflammatory changes nor pneumonic focus (Fig. 14), the kidneys did not show cellular changes compatible with inflammation and infection (Photo 15), the eyeballs did not show neither conjunctivitis nor

keratitis signs, looking at the cornea, the front and rear chamber of the eye, as well as the retina without changes (Photo 16).

Photo 12. Liver cell unaltered (40X). Hematoxylin eosin

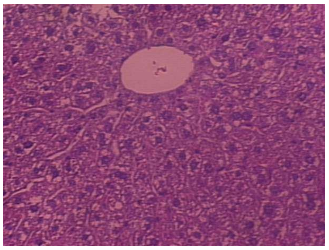

Photo 13. Spleen. Normal relationship between red pulp and white pulp (40X). Hematoxylin eosin

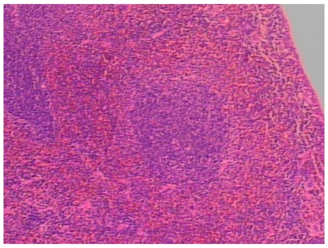

Photo 14. Renal cortex unaltered (40X). Hematoxylin eosin

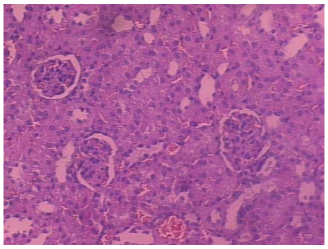

Photo 15. Unaltered respiratory alveoli (40X). Hematoxylin eosin

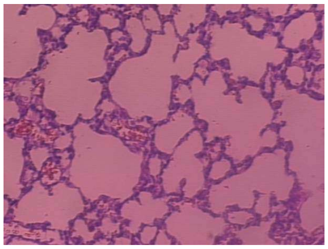

Photo 16. Eyeball unaltered (40X). Hematoxylin eosin

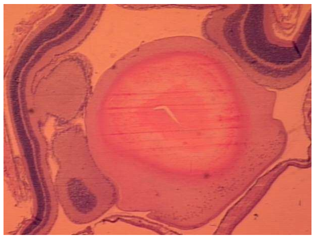

CONCLUSIONS

We can conclude that *Pseudomonas florescens* suspension found in the biofertilizer RIZOFOS LlQ provided by RIZOBACTER ARGENTINA S.A. did not prove to be aggressive when inoculating 2,8 x 10^8 bacteria per individual, in physiologically normal mice as well as in stressed by hypothermia and immobilization ones.

Delia Susana Oriani
Special Assistant Professor Microbiology
Veterinary Medical
Clinical and Industrial Bacteriologist
MSCs. Agricultural

Bibliography:

Brock (2004). Biología de los Microorganismos. Madigan, M.; Martinko, J.; Parker, J. (Microorganisms Biology)

Domínguez Abraham Quezada. (1997). Introducción al manejo de animales de laboratorio: roedores y pequeñas especies. Ediciones de la Universidad Autónoma de Yucatán México. (Introduction to management of laboratory animals: rodents and little species)

Esnard BolaIlos.S.C.; Díaz Rosa, O. (1997). Identificación y caracterización de bacilos Gram negativos no fermentadores en el medio hospitalario. Rev Cubana Hig Epidemiol v.35 n.1 . Ciudad de la Habana (Identification and characterization of Gram negative bacilli)

Todar Kcnneth (2004) Pseudomonas and Related Bacteria. In Textbook of Bacteriology.

Toso, Ricardo. 2001. Tesis doctoral UNS Actividad Gastroprotectora de Extractos de Centaurea solsitialis. (Doctoral thesis UNS gastroprotector Activity of Centaurea solsitialis extracts)

Supplementary Data 5

Program	OmniLog 2.3
User	Anja
Data File	IDE_357_211123_B.D5E
Data Location	C:\Program Files\Biolog\OL_DC_23\IDE\Data_2021\11\

Instrument	OmniLog	Project	IDE
Instrument S/N	357	Start Time	Nov 23 2021 8:38 AM
Data Mode	ID	Lapse Time	Nov 23 2021 6:47 PM
Read Mode	Normal ID	ID Called At	10.00Hrs
Plate Position	24-A	Target Incubation	22 Hrs
Plate Type	GEN III		
Plate Protocol	A		

Sample ID	21-725
Field 2	afr
Field 3	T-95%
Field 4	23.11.21 8.00
Field 5	
Field 6	
Field 7	
Field 8	
Field 9	
Field 10	

Biolog Database	Biolog GEN III 2_6_1_08.I5G
Status	Final ID

Result	Species ID: Pseudomonas fluorescens
Comment	
Notice	

Rank	SIM	DIST	PROB	Organism Type	Species
1	0.533	2.906	0.671	GN-Nent	Pseudomonas fluorescens
2	0.122	3.801	0.166	GN-Nent	Pseudomonas fragi
3	0.073	4.114	0.102	GN-Nent	Pseudomonas lundensis
4	0.042	4.441	0.061	GN-Nent	Pseudomonas aeruginosa

Key: <x: positive, x: negative, <x-: mismatched positive, x+: mismatched negative, {x: borderline, -x: less than A1 well

Well Color Values

Plate	1	2	3	4	5	6	7	8	9	10	11	12
A	57	{ 74	40	60	44	50	42	52	51	< 166	{ 142	81
B	53	40	56	44	42	40	43	45	48	{ 142	62	68
C	{ 89	56	{ 81	{ 80	58	{ 108	{ 100	70	44	{ 138	{ 134	{ 133
D	57	48	43	41	45	48	{ 114 -	54	47	{ 115	{ 149	66 +
E	51	46	50	43	46	52	44	45	59	{ 143	{ 102	{ 150
F	61	{ 100	71	57	{ 96	< 140 -	56	{ 94	56	{ 146	< 252	< 217
G	52	54	44	55	66	60	53	58	67	89 +	72	< 167
H	66	64	55	62	53	57	40	{ 83	66	{ 150	66	94

Report Date: November 23 2021 6:47 PM (report version: 2.3)

Supplementary Data 6

Volume: DATA File: E21C063.98A Samp Ctr: 9 ID Number: 1869
Type: Samp Bottle: 12 Method: TSBA6
Created: 12/6/2021 12:39:22 PM
Sample ID: UN-ID-21-725(M.535,1d,28C) GC/MS: FAME 211208_ave01; DMDS 211213_gem13; DMOX 211213_gem12

RT	Response	Ar/Ht	RFact	ECL	Peak Name	Percent	Comment1	Comment2	GC-MS
1.681	5.204E+8	0.027	----	7.017	SOLVENT PEAK	----	< min rt		
2.077	867	0.025	----	7.819		----	< min rt		
2.242	925	0.024	----	8.157		----	< min rt		
2.477	278	0.022	----	8.632		----	< min rt		
2.637	2120	0.026	----	8.956		----	< min rt		
3.075	557	0.029	----	9.840		----			
3.154	811	0.030	1.127	10.000	10:0	0.10	ECL deviates 0.000	Reference 0.004	OK
3.478	743	0.028	----	10.474		----			
4.222	26795	0.030	1.047	11.423	10:0 3OH	3.01	ECL deviates 0.001		OK
4.746	20851	0.033	1.023	12.000	12:0	2.29	ECL deviates 0.000	Reference 0.006	OK
5.144	245	0.028	----	12.350		----			
5.298	723	0.032	----	12.486	unknown 12.484	----	ECL deviates 0.002		
5.828	355	0.037	----	12.952		----			
6.123	40806	0.037	0.985	13.176	12:0 2OH	4.32	ECL deviates -0.001		OK
6.273	691	0.041	0.982	13.287	12:1 3OH	0.07	ECL deviates -0.001		Not confirmed
6.499	35422	0.036	0.978	13.454	12:0 3OH	3.72	ECL deviates 0.000		OK
6.985	1729	0.038	----	13.812		----			Identified as 14:1 w7c
7.238	3104	0.036	0.967	13.998	14:0	0.32	ECL deviates -0.002	Reference 0.005	OK
8.768	1267	0.041	----	15.000	15:0	----	ECL deviates 0.000		OK
10.125	289235	0.045	0.948	15.820	Sum In Feature 3	29.45	ECL deviates -0.002	16:1 w7c/16:1 w6c	Identified as 16:1 w7c
10.272	1017	0.043	0.947	15.909	16:1 w5c	0.10	ECL deviates 0.000		OK
10.427	298589	0.044	0.947	16.002	16:0	30.37	ECL deviates 0.002	Reference 0.008	OK
10.579	1923	0.061	----	16.091		----			
11.505	1019	0.044	0.945	16.629	17:0 iso	0.10	ECL deviates -0.001	Reference 0.004	OK
11.788	1337	0.054	0.945	16.793	17:1 w8c	0.14	ECL deviates 0.001		Not confirmed
11.952	89872	0.044	0.945	16.889	17:0 cyclo	9.13	ECL deviates 0.001		Identified as 17:0 Cyclo w7c
12.144	1800	0.040	0.945	17.001	17:0	0.18	ECL deviates 0.001	Reference 0.006	OK
13.463	1291	0.045	----	17.753		----			
13.589	150318	0.047	0.947	17.824	Sum In Feature 8	15.29	ECL deviates 0.001	18:1 w7c	Identified as 18:1 w7c
13.895	5834	0.046	0.947	17.999	18:0	0.59	ECL deviates -0.001	Reference 0.003	OK
14.038	2419	0.044	0.947	18.081	18:1 w7c 11-methyl	0.25	ECL deviates 0.000		OK
14.668	1683	0.048	----	18.443		----			
15.032	867	0.044	----	18.652		----			
15.371	1023	0.042	0.950	18.846	Sum In Feature 7	0.10	ECL deviates 0.000	un 18.846/19:1 w6c	Not confirmed
15.470	4486	0.048	0.950	18.903	19:0 cyclo w8c	0.46	ECL deviates 0.001		Identified as 19:0 Cyclo w7c
15.583	666	0.044	----	18.969		----			
----	289235	---	----	----	Summed Feature 3	29.45	16:1 w7c/16:1 w6c	16:1 w6c/16:1 w7c	
----	1023	---	----	----	Summed Feature 7	0.10	un 18.846/19:1 w6c	19:1 w6c/.846/19cy	
----	-----	---	----	----		----	19:0 cyclo w10c/19w6		
----	150318	---	----	----	Summed Feature 8	15.29	18:1 w7c	18:1 w6c	

ECL Deviation: 0.001 Reference ECL Shift: 0.005 Number Reference Peaks: 7
Total Response: 985488 Total Named: 975430
Percent Named: 98.98% Total Amount: 930865

Matches:

Library	Sim Index	Entry Name
TSBA6 6.21	0.904	Pseudomonas-putida-biotype B/vancouverensis
	0.772	Pseudomonas-putida-biotype A
	0.588	Herbaspirillum-autotrophicum

[TSBA6] Pseudomonas-putida-biotype B/vancouverensis
Sim Index: 0.904 (Distance: 1.596)

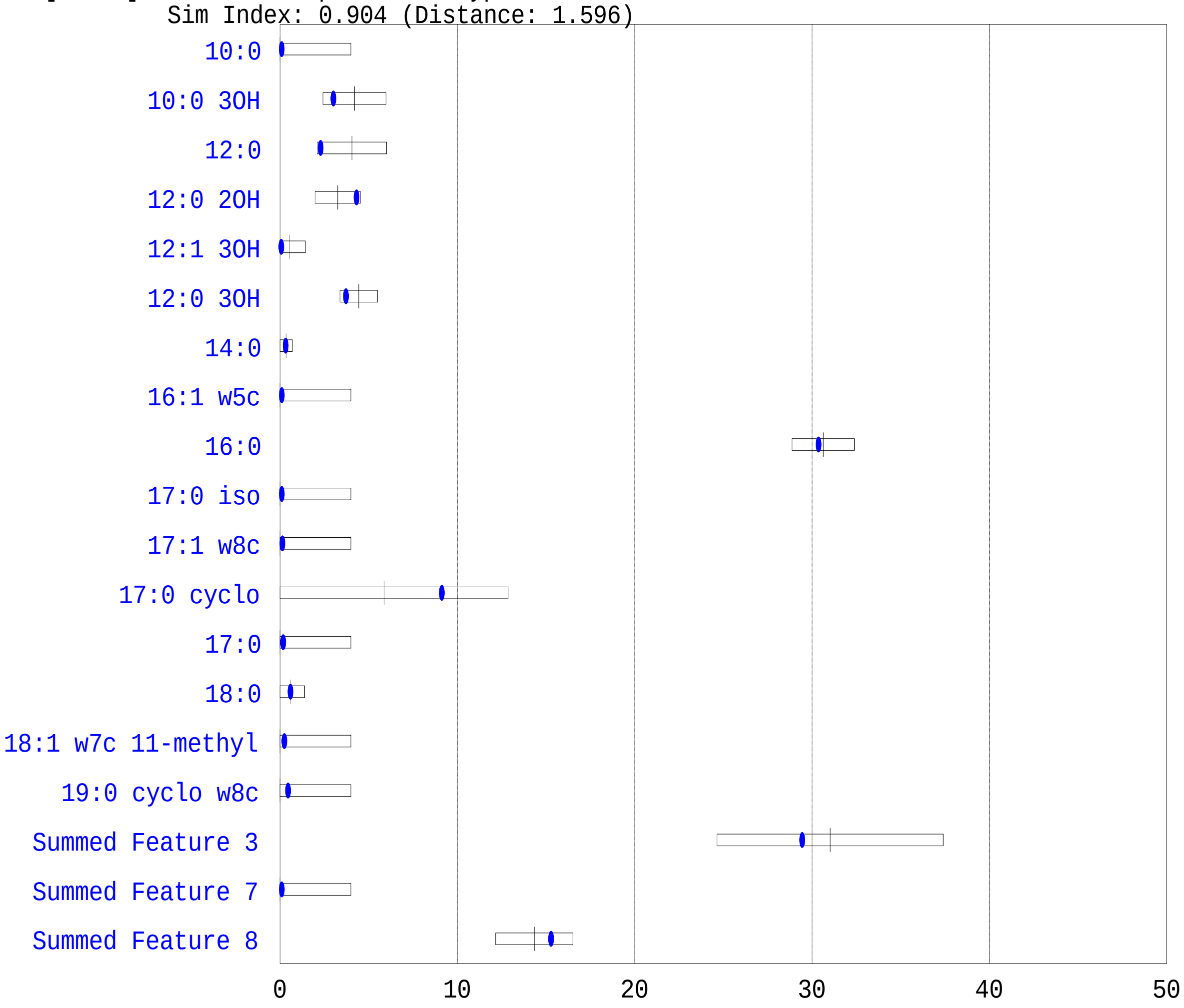

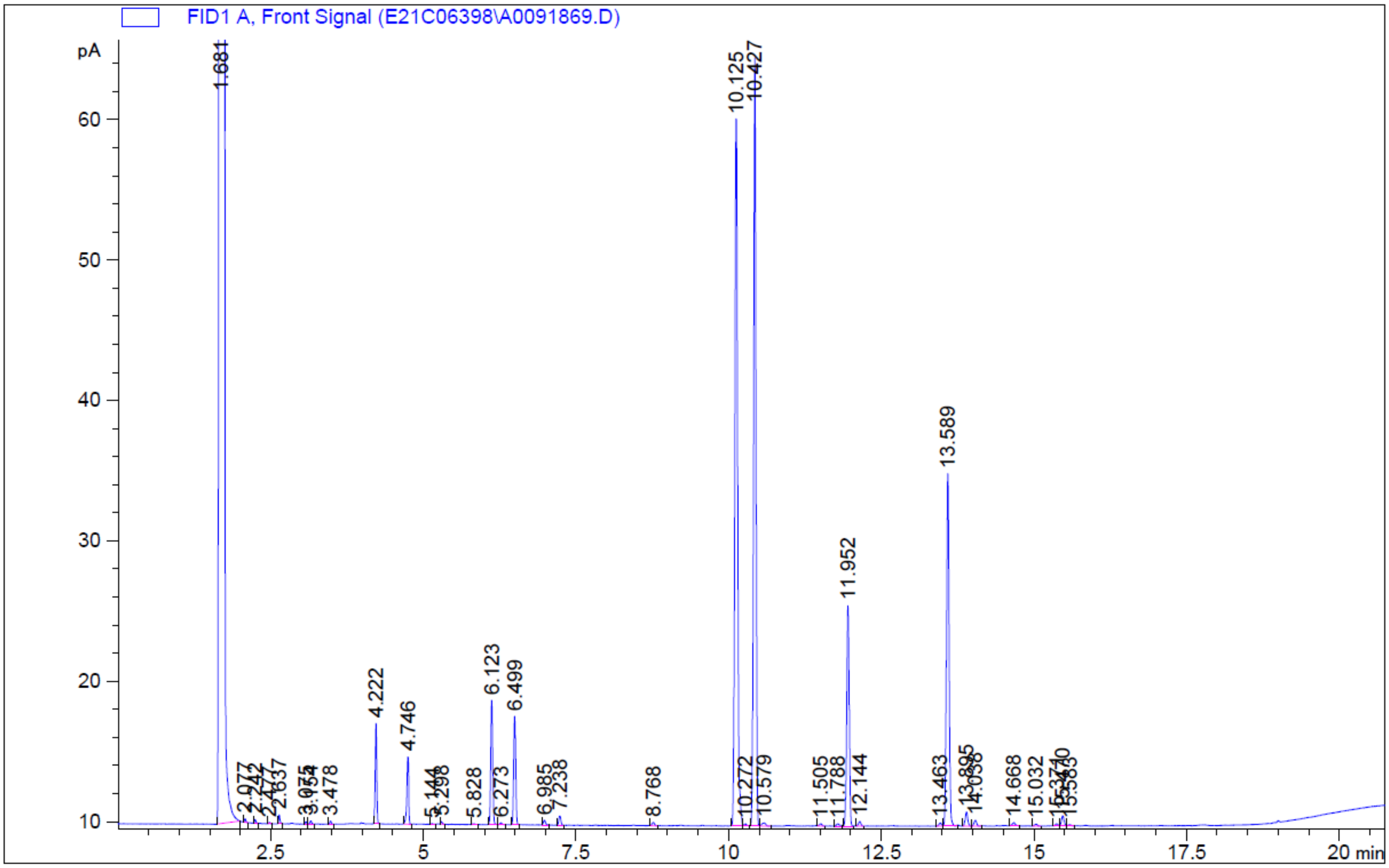
FID1 A, Front Signal (E21C06398\A0091869.D)
pA
min
1.681
4.222
4.746
6.123
6.499
10.125
10.427
11.952
13.589
