# Supplementary material for: Agronomic efficiency and genome mining analysis of the wheat-biostimulant rhizospheric bacterium Pseudomonas pergaminensis sp. nov. strain 1008T
Source: Front Plant Sci. 2022 Jul 28;13:894985. doi: 10.3389/fpls.2022.894985 (PMC9369656; doi:10.3389/fpls.2022.894985)
Supplement: Supplementary file 2 [file Data_Sheet_2.docx]

**Supplementary Figure 1.** Phylogenetic relationships of *Pseudomonas* sp. strain 1008 on the basis of its 16S rDNA gene sequence. Tree inferred with FastME 2.1.6.1 ([Lefort et al., 2015](#_ENREF_5)) from GBDP distances calculated from 16S rDNA gene sequences of the closely related type species. The branch lengths are scaled in terms of GBDP distance formula d5. The numbers above branches are GBDP pseudo-bootstrap support values > 60 % from 100 replications, with an average branch support of 61.9 %. The tree was rooted at the midpoint ([Farris, 1972](#_ENREF_1)). All type species shown in the graph belong to the *P. fluorescens* subgroup within the *P. fluorescens* lineage ([Lalucat et al., 2020](#_ENREF_4)). The figure was generated with the Type (Strain) Genome Server (TYGS) (<https://tygs.dsmz.de/>).

**
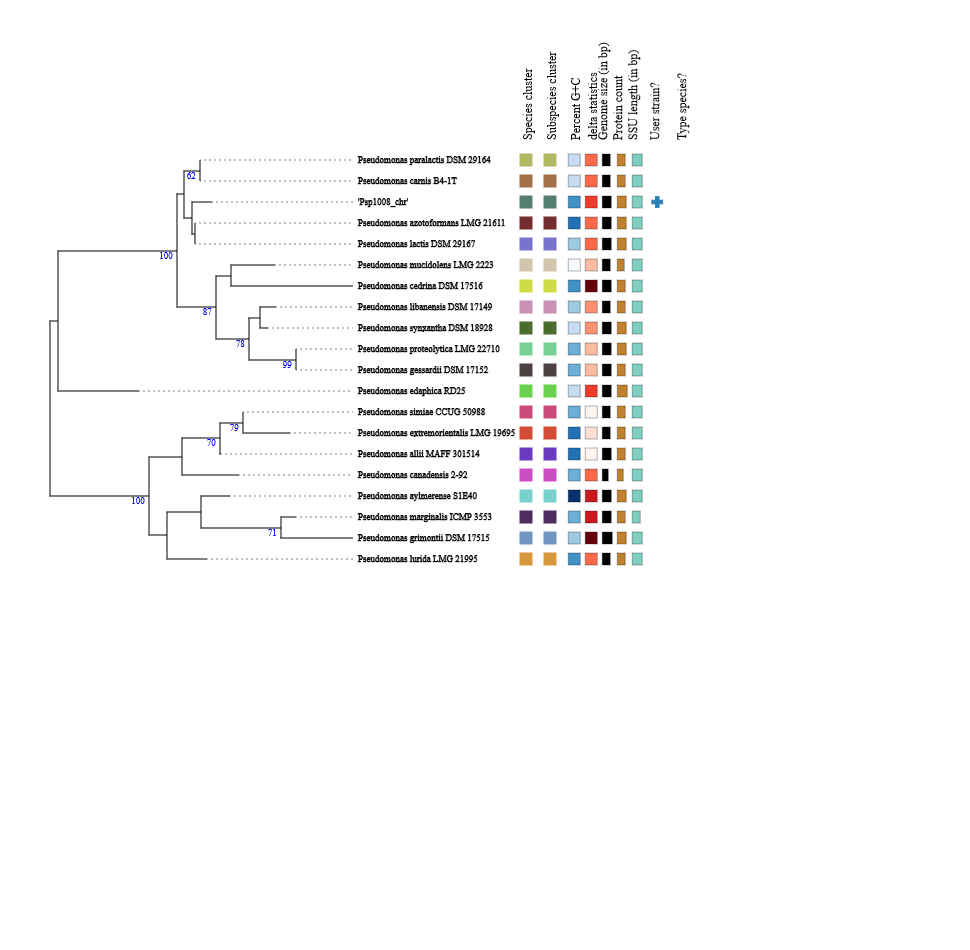
**

**Supplementary Figure 2.** Phenotypic characterization of *Pseudomonas* sp. strain 1008.


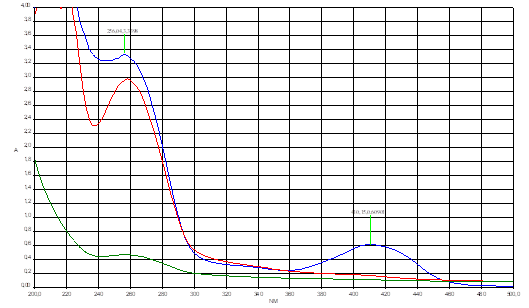

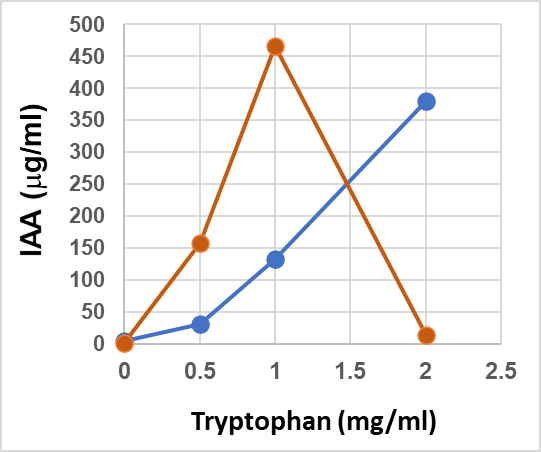

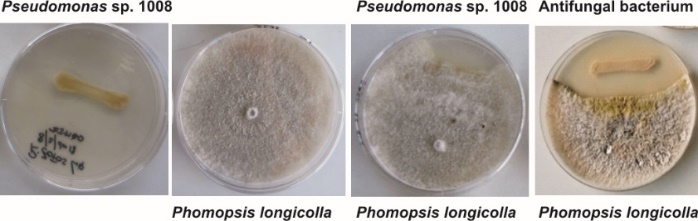

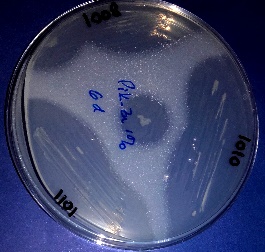

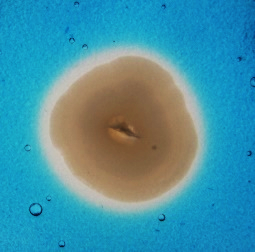


**g**

**1008**

**a**

**b**

**c**

**d**

**e**

**f**

**a.** Solubilization of ZnO (1% w/v) in Pikovskaya agar. The streak of strain 1008 is labelled in the upper part of the plate. **b.** Halo of protease activity around a macrocolony of strain 1008 on skim-milk agar. **c.** Halo of lecithinase (phospholipase) activity around a macrocolony of strain 1008 on egg-yolk agar. **d.** Halo of CAS out-competition by siderophores produced by a macrocolony of strain 1008 on CAS agar. **e.** IAA production in the supernatant of strain 1008 grown for 24 h (blue symbols) or 48 h (orange symbols) in the presence of different concentrations of tryptophan. **f.** Evaluation of antifungal activity with the dual culture assay on PDA plates. The assay is illustrated for *Phomopsis longicolla* as the test fungus. Plates were incubated at 25 °C in the dark for 7 days. Similar negative results for fungal inhibition by strain 1008 were observed for *Bipolaris sorokiniana*, *Rhizoctonia solani* strains R1 and R24, *Alternaria* sp., *Pythium debaryanum*, *Fusarium graminearum*, *Drechslera tritici-repentis*, and *Sclerotinia sclerotiorum*. **g**. UV-visible spectrum (200-500 nm) of supernatants from cultures of strain 1008. Green line: non-inoculated growth medium; red line, supernatant sampled at 6 h of growth in iron-replete medium; blue line, supernatant sampled at 6 h of growth in iron-deficient medium.

The characteristic pyoverdine peak is visible at 400 nm (blue line).

**Supplementary Figure 3.** Comparison of cellular structure and 16S rDNA sequence of phase variants 1 and 2 of *Pseudomonas* sp. strain 1008.


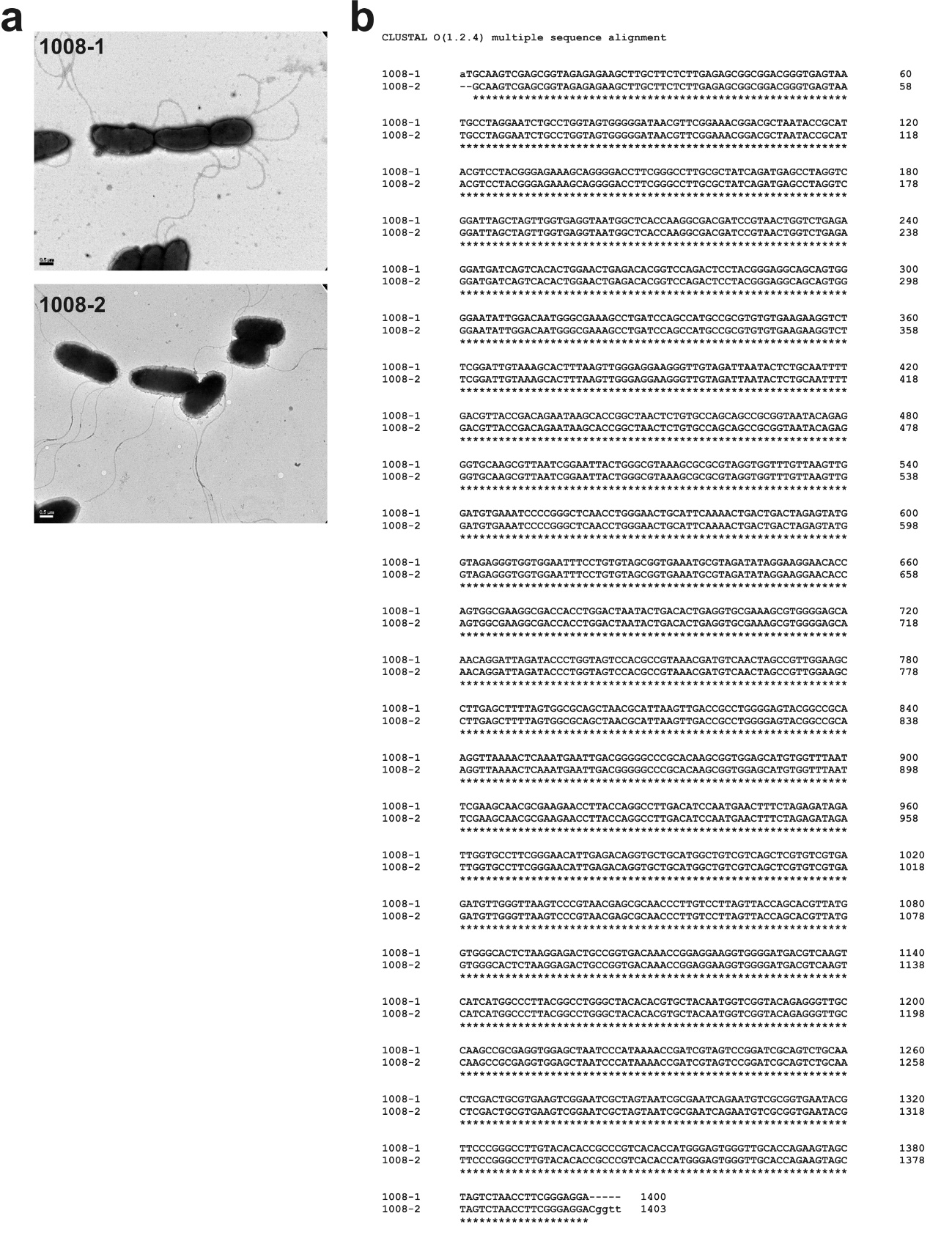


**a.** Transmission electron micrographs showing cells of *Pseudomonas* sp. strain 1008 from colony morphotypes 1 and 2, grown for 24 h in nutrient yeast broth at 28 °C and 200 rpm. **b.** Sequence alignment of partial 16S rDNA sequences from both colony morphotypes. Sequences are 100% identical along 1,400 bp.

**Supplementary Figure 4.** Identification of an integrative and conjugative element (ICE) in the chromosome of *Pseudomonas* sp. strain 1008 with the ICEfinder tool (<https://bioinfo-mml.sjtu.edu.cn/ICEfinder/index.php>).

**Supplementary Figure 5.** Identification of prophage regions in the chromosome of *Pseudomonas* sp. strain 1008.


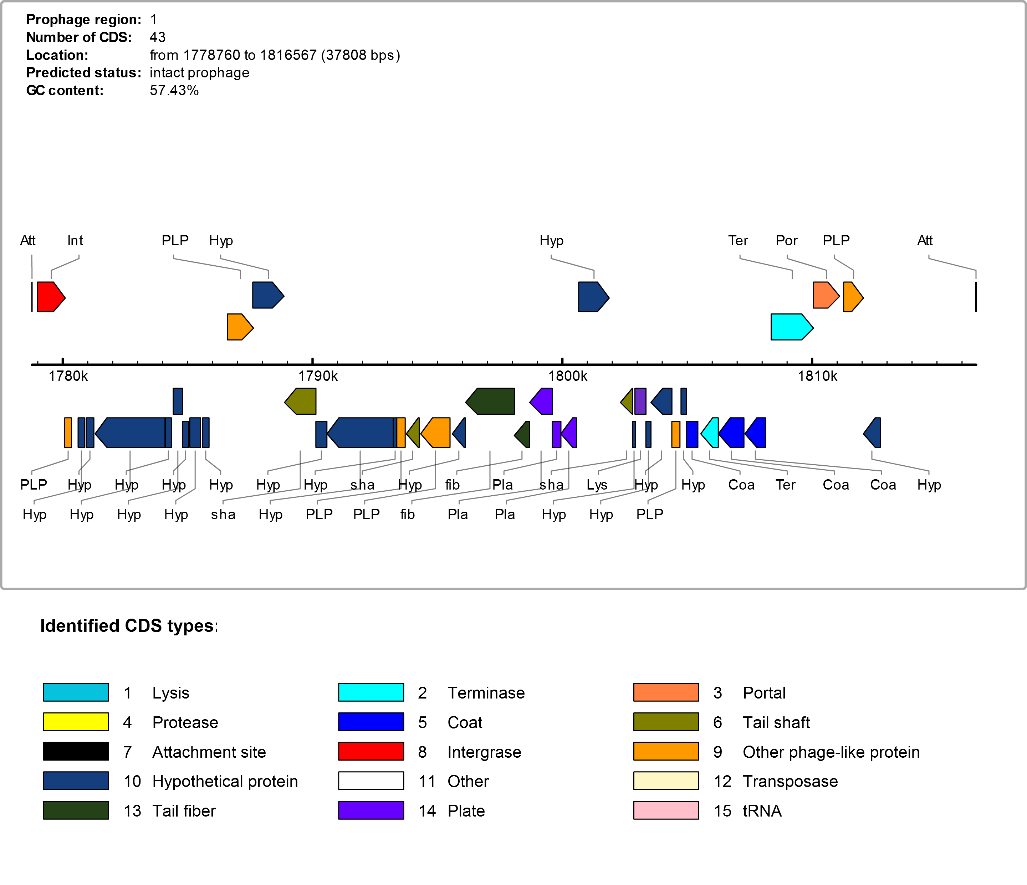


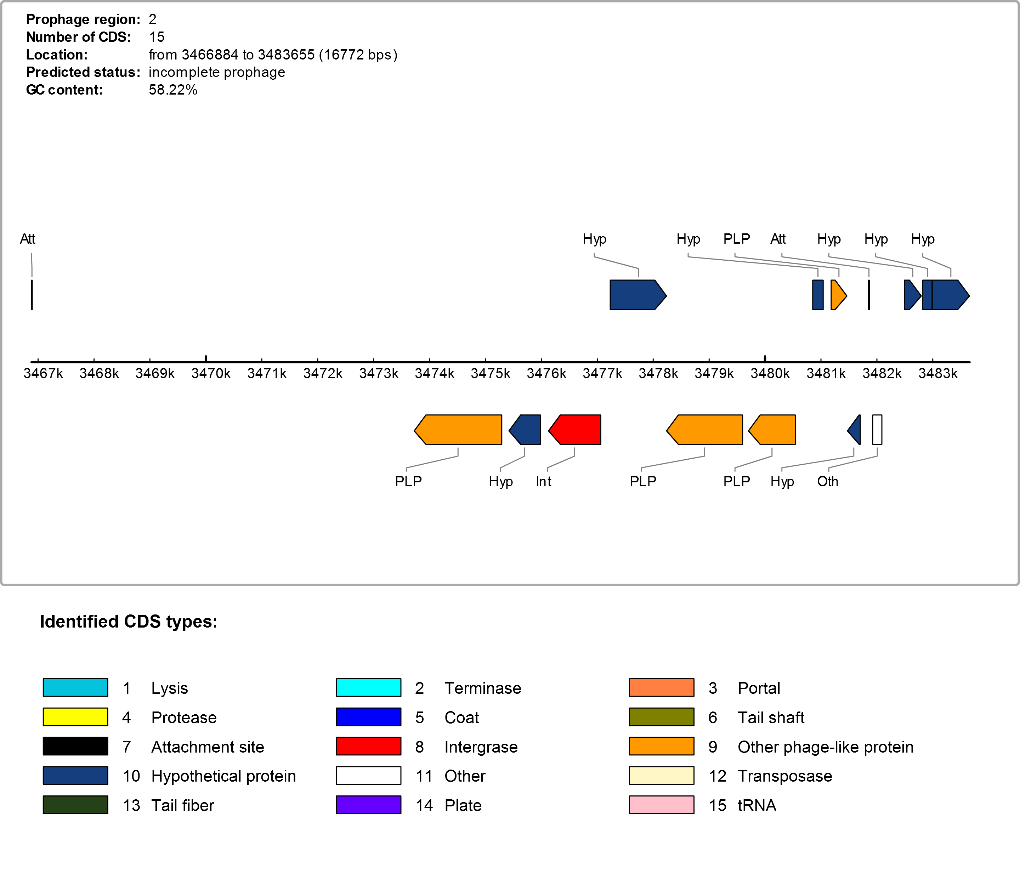


**a**. Prophage region 1 (intact prophage). **b**. Prophage region 2 (incomplete prophage). Prophage loci were identified with the Phaster tool (<https://phaster.ca/>).

**Supplementary Figure 6.** Output of the PathogenFinder tool from the Center for Genomic Epidemiology (<https://cge.cbs.dtu.dk/services/PathogenFinder/>) for the analysis of *Pseudomonas* sp. strain 1008 genome.


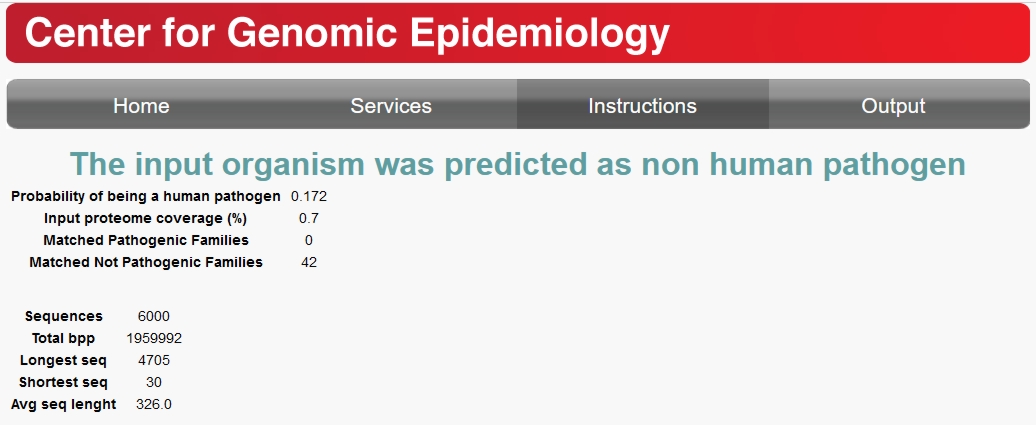


**Supplementary Figure 7.** Genome to genome comparison between *Pseudomonas* sp. strain 1008 and *P. azotoformans* strain F77.


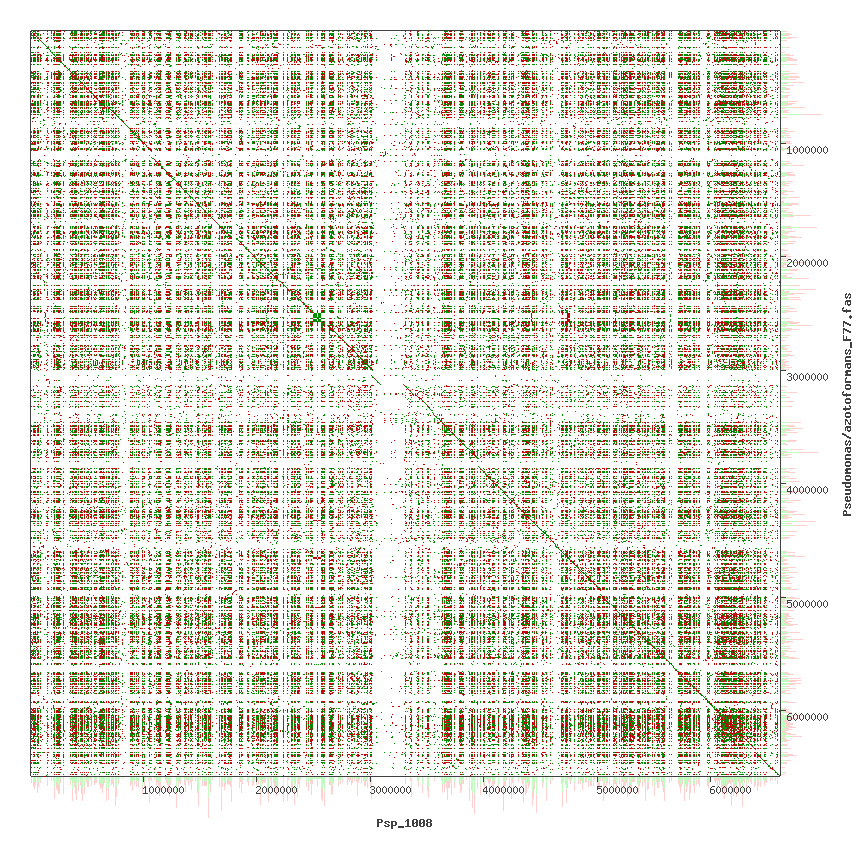

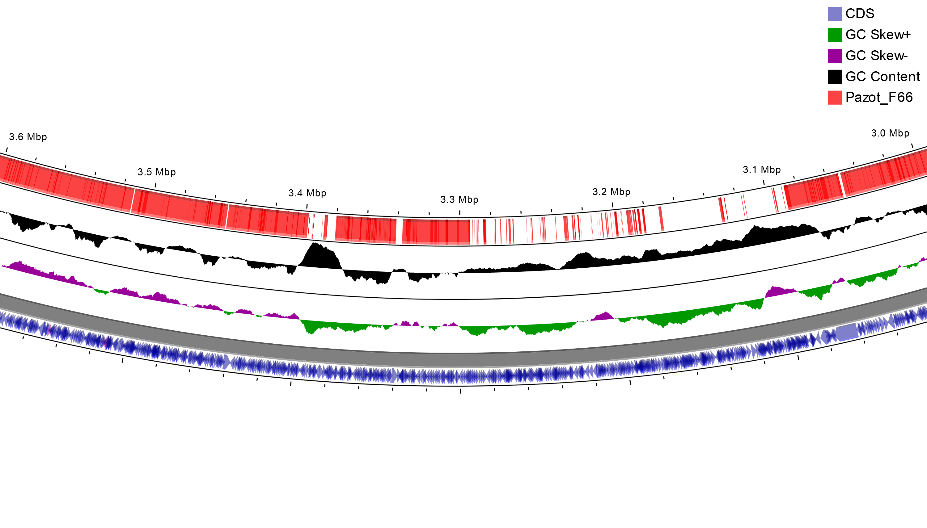


**a**

**b**

F77

**a.** Synteny matrix plot showing the comparison of the whole genomes of *Pseudomonas* sp. strain 1008 and *P. azotoformans* strain F77. The plot was generated with the Synteny plot tool of the EDGAR platform (<https://edgar3.computational.bio.uni-giessen.de/cgi-bin/edgar.cgi>). **b.** Close-up of the circular representation of the genome-to-genome alignment in the region of maximum dissimilarity between both genomes (positions 3.1-3.3 Mbp for strain 1008). The circular map was generated with the CGView server^BETA^ (<http://cgview.ca/>).

**Supplementary Figure 8.** Phylogenetic relationships of the T3SS components of *Pseudomonas* sp. strain 1008 with beneficial and phytopathogenic (*P. syringae* isolates) *Pseudomonas* spp.


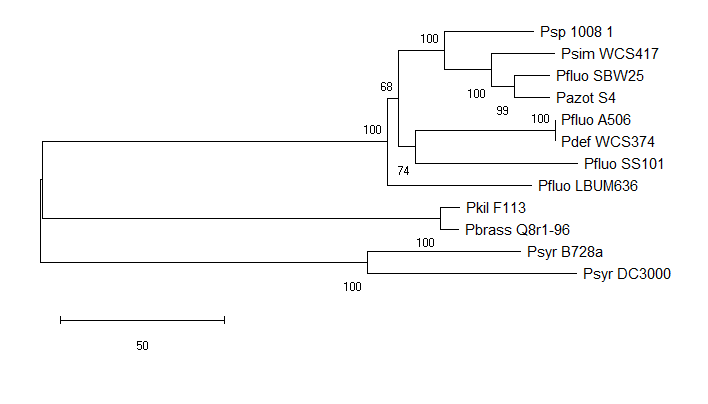

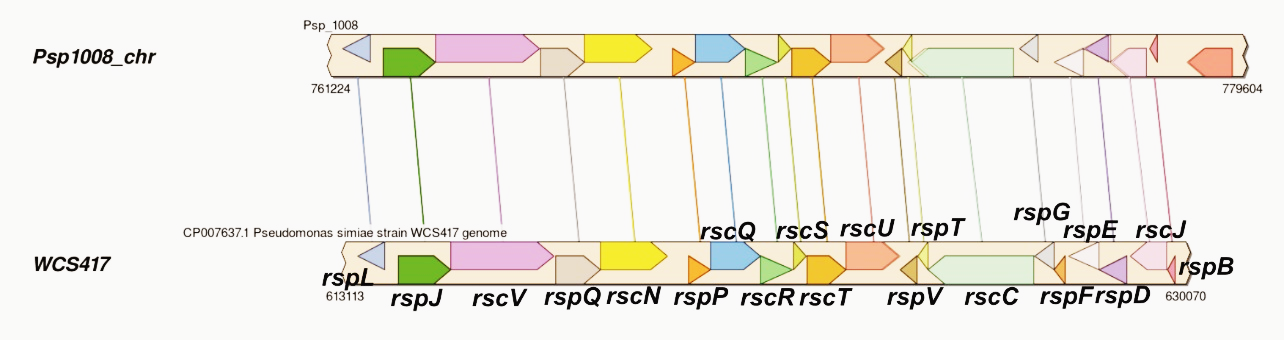


**a**

**b**

**a**. Phylogenetic relationships of the *Pseudomonas* sp. strain 1008 T3SS component RscC (secretin) with other beneficial and phytopathogenic *Pseudomonas* spp. ([Nazir et al., 2017](#_ENREF_6)). The evolutionary history was inferred using the Neighbor-Joining method ([Saitou and Nei, 1987](#_ENREF_8)). The optimal tree with the sum of branch length = 697.78906250 is shown. The percentage of replicate trees in which the associated taxa clustered together in the bootstrap test (500 replicates) are shown next to the branches ([Felsenstein, 1985](#_ENREF_2)). The tree is drawn to scale, with branch lengths in the same units as those of the evolutionary distances used to infer the phylogenetic tree. The evolutionary distances were computed using the number of differences method ([Nei and Masatoshi, 2000](#_ENREF_7)) and are in the units of the number of amino acid differences per sequence. The analysis involved 12 amino acid sequences. All positions containing gaps and missing data were eliminated. There were a total of 592 positions in the final dataset. Evolutionary analyses were conducted in MEGA X ([Kumar et al., 2018](#_ENREF_3)). **b**. Synteny between the T3SS loci of *Pseudomonas* sp. strain 1008 and *P. simiae* WCS417 ([Stringlis et al., 2019](#_ENREF_9)). The figure was generated with the SimpleSynteny server (<https://www.dveltri.com/simplesynteny/>).

**Supplementary Figure 9.** Identification of type 6 secretion systems in the chromosome of *Pseudomonas* sp. strain 1008.


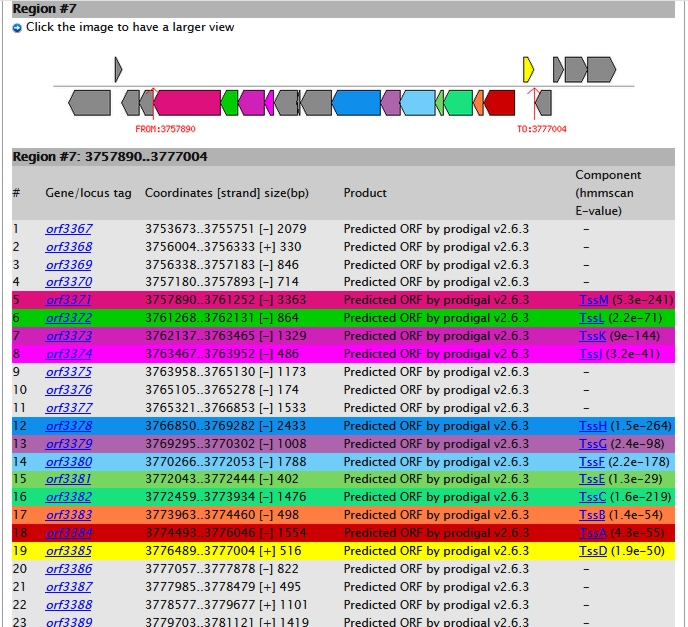


**a**


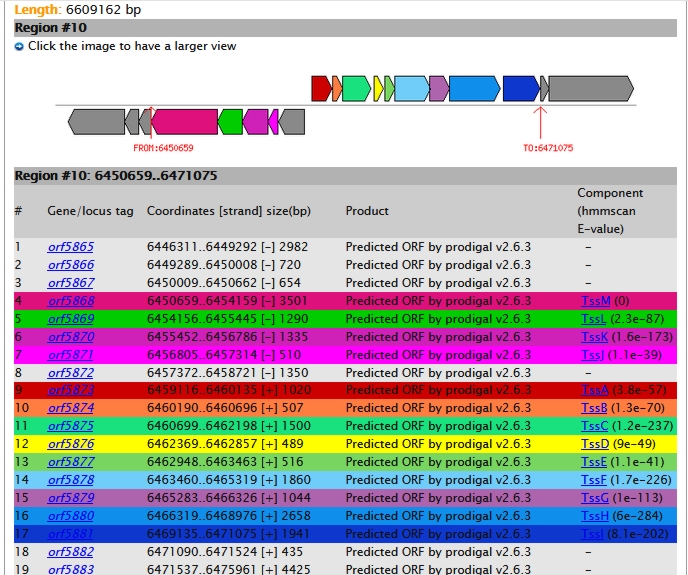


**b**

**a**. T6SS locus 1. **b**. T6SS locus 2. The loci were identified with SecReT6 server (<https://bioinfo-mml.sjtu.edu.cn/SecReT6/t6ss_prediction.php>).

**Supplementary Figure 10.** Graphical summary of the metabolic profiling of *Pseudomonas* sp. strain 1008 with the Biolog GenIII microplate system (DSMZ Services; see raw data in Supp. Data 6.pdf file).


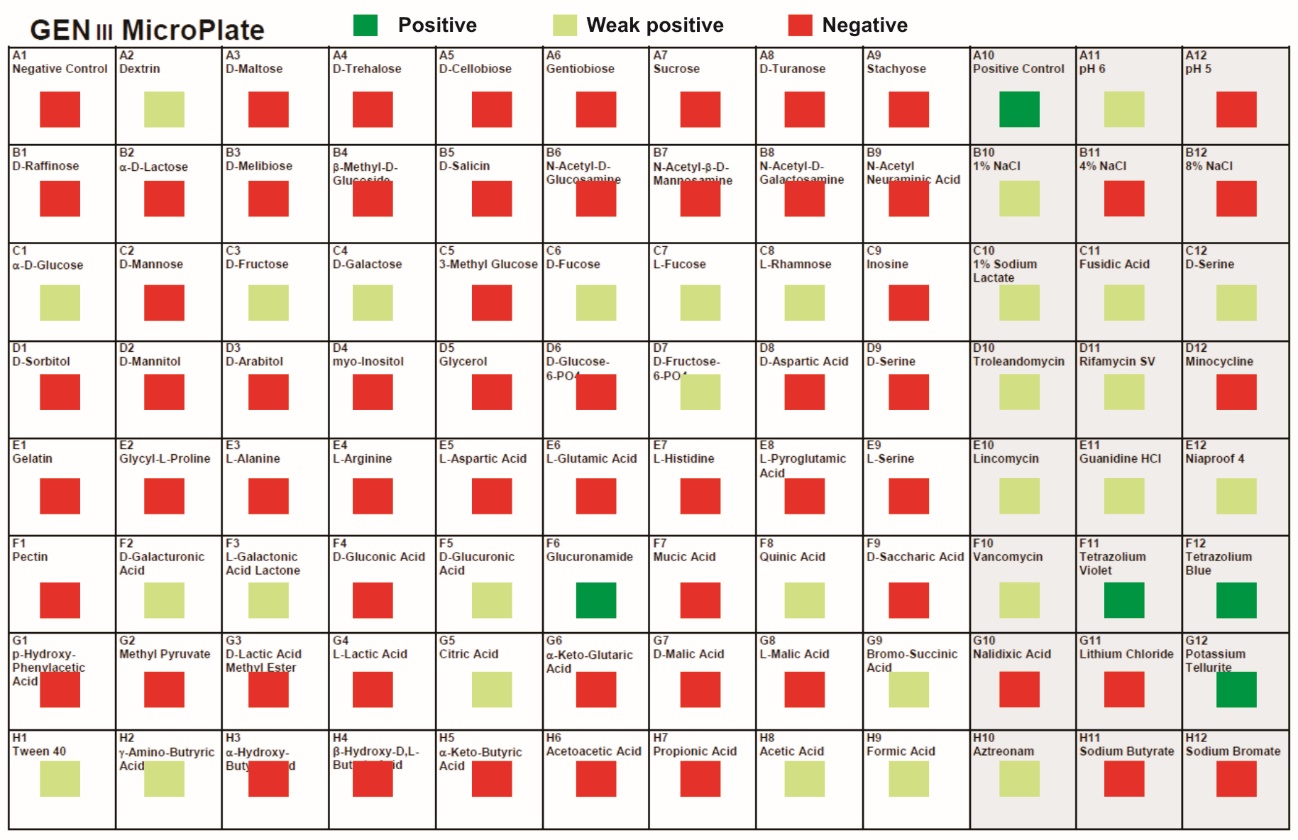


**References**

Farris, J.S. (1972). Estimating phylogenetic trees from distance matrices. *The american naturalist* 106**,** 645-668. doi

Felsenstein, J. (1985). Confidence limits on phylogenies: An approach using the bootstrap. *Evolution* 39**,** 783-791. doi 10.1111/j.1558-5646.1985.tb00420.x

Kumar, S., Stecher, G., Li, M., Knyaz, C., and Tamura, K. (2018). MEGA X: Molecular evolutionary genetics analysis across computing platforms. *Mol Biol Evol* 35**,** 1547-1549. doi 10.1093/molbev/msy096

Lalucat, J., Mulet, M., Gomila, M., and Garcia-Valdes, E. (2020). Genomics in bacterial taxonomy: Impact on the genus *Pseudomonas*. *Genes (Basel)* 11. doi 10.3390/genes11020139

Lefort, V., Desper, R., and Gascuel, O. (2015). FastME 2.0: A comprehensive, accurate, and fast distance-based phylogeny inference program. *Mol Biol Evol* 32**,** 2798-2800. doi 10.1093/molbev/msv150

Nazir, R., Mazurier, S., Yang, P., Lemanceau, P., and Van Elsas, J.D. (2017). The Ecological Role of Type Three Secretion Systems in the Interaction of Bacteria with Fungi in Soil and Related Habitats Is Diverse and Context-Dependent. *Front Microbiol* 8**,** 38. doi 10.3389/fmicb.2017.00038

Nei, M., and Masatoshi, S.K. (eds.). (2000). *Molecular Evolution and Phylogenetics.* Oxford: Oxford University Press. 0195135849

Saitou, N., and Nei, M. (1987). The neighbor-joining method: a new method for reconstructing phylogenetic trees. *Mol Biol Evol* 4**,** 406-425. doi 10.1093/oxfordjournals.molbev.a040454

Stringlis, I.A., Zamioudis, C., Berendsen, R.L., Bakker, P., and Pieterse, C.M.J. (2019). Type III Secretion System of beneficial rhizobacteria *Pseudomonas simiae* WCS417 and *Pseudomonas defensor* WCS374. *Front Microbiol* 10**,** 1631. doi 10.3389/fmicb.2019.01631
