# Supplementary material for: Agronomic efficiency and genome mining analysis of the wheat-biostimulant rhizospheric bacterium Pseudomonas pergaminensis sp. nov. strain 1008T
Source: Front Plant Sci. 2022 Jul 28;13:894985. doi: 10.3389/fpls.2022.894985 (PMC9369656; doi:10.3389/fpls.2022.894985)
Supplement: Supplementary file 3 [file Table_1.docx]

**Supplementary Table 1**. Climatic data during the field trials to evaluate the agronomic efficiency of bacterizing wheat seeds with *Pseudomonas* sp. strain 1008.
