# Supplementary material for: Agronomic efficiency and genome mining analysis of the wheat-biostimulant rhizospheric bacterium Pseudomonas pergaminensis sp. nov. strain 1008T
Source: Front Plant Sci. 2022 Jul 28;13:894985. doi: 10.3389/fpls.2022.894985 (PMC9369656; doi:10.3389/fpls.2022.894985)
Supplement: Supplementary file 5 [file Table_3.docx]

**Supplementary Table 3**. Results of the bioMérieux API 20 NE Gallery System for *Pseudomonas* sp. strain 1008. +, positive result; +w, weak positive result; -, negative result.

| NO_3_^-^ | Trp | Glu_f_ | ADH | URE | ESC | GEL | PNPG | GLU_A_ | ARA |
| --- | --- | --- | --- | --- | --- | --- | --- | --- | --- |
| +w | - | - | - | - | - | - | - | +w | +w |
| MNE | MAN | NAG | MAL | GNT | CAP | ADI | MLT | CIT | PAC |
| +w | +w | +w | - | + | +w | + | + | +w | - |
