# Supplementary material for: Agronomic efficiency and genome mining analysis of the wheat-biostimulant rhizospheric bacterium Pseudomonas pergaminensis sp. nov. strain 1008T
Source: Front Plant Sci. 2022 Jul 28;13:894985. doi: 10.3389/fpls.2022.894985 (PMC9369656; doi:10.3389/fpls.2022.894985)
Supplement: Supplementary file 6 [file Table_4.docx]

**Supplementary Table 4**. Efficiency of *Pseudomonas* sp. strain 1008 to increase the yield of wheat in field trials. The effect of seed bacterization with Rizofos® containing live cells of strain 1008 was analyzed individually for each of the 26 field assays by means of ANOVA. Each assay involved three replicate plots for untreated seeds (control) and three replicate plots for bacterized seeds (+Rizofos®).
