# Supplementary material for: Agronomic efficiency and genome mining analysis of the wheat-biostimulant rhizospheric bacterium Pseudomonas pergaminensis sp. nov. strain 1008T
Source: Front Plant Sci. 2022 Jul 28;13:894985. doi: 10.3389/fpls.2022.894985 (PMC9369656; doi:10.3389/fpls.2022.894985)
Supplement: Supplementary file 7 [file Table_5.docx]

**Supplementary Table 5**. Genes related to antibiotic resistance in the genome of *Pseudomonas* sp. strain 1008, identified with the Resistance Gene Identifier tool of the Comprehensive Antibiotic Resistance Database (<https://card.mcmaster.ca/analyze/rgi>).
