# Supplementary material for: Agronomic efficiency and genome mining analysis of the wheat-biostimulant rhizospheric bacterium Pseudomonas pergaminensis sp. nov. strain 1008T
Source: Front Plant Sci. 2022 Jul 28;13:894985. doi: 10.3389/fpls.2022.894985 (PMC9369656; doi:10.3389/fpls.2022.894985)
Supplement: Supplementary file 8 [file Table_6.docx]

**Supplementary Table 6**. Summary of CRISPR-Cas loci detected in the genome of *Pseudomonas* sp. strain 1008 with the CRISPRCasFinder (<https://crisprcas.i2bc.paris-saclay.fr/>) and CRISPRMiner2 (<http://www.microbiome-bigdata.com/CRISPRminer2/index/>) tools.
